# Synthesis of CF_3_-Containing Spiro-[Indene-Proline] Derivatives via Rh(III)-Catalyzed C-H Activation/Annulation

**DOI:** 10.3390/molecules28237809

**Published:** 2023-11-27

**Authors:** Alexandra S. Bubnova, Daria V. Vorobyeva, Ivan A. Godovikov, Alexander F. Smol’yakov, Sergey N. Osipov

**Affiliations:** A. N. Nesmeyanov Institute of Organoelement Compounds, Russian Academy of Sciences, 28/1 Vavilova Str., 119334 Moscow, Russia; bubnova.axs@gmail.com (A.S.B.); vorobyeva-daria@yandex.ru (D.V.V.); pr0vider@ineos.ac.ru (I.A.G.); rengenhik@gmail.com (A.F.S.)

**Keywords:** C-H activation, spiro cyclization, rhodium catalysis, fluorinated proline derivatives

## Abstract

An efficient method of accessing new CF_3_-containing spiro-[indene-proline] derivatives has been developed based on a Cp*Rh(III)-catalyzed tandem C-H activation/[3+2]–annulation reaction of 5-aryl-2-(trifluoromethyl)-3,4-dihydro-2*H*-pyrrole-2-carboxylates with alkynes. An important feature of this spiro annulation process is the feasibility of dehydroproline moiety to act as a directing group in the selective activation of the aromatic C-H bond.

## 1. Introduction

**Figure 1:**
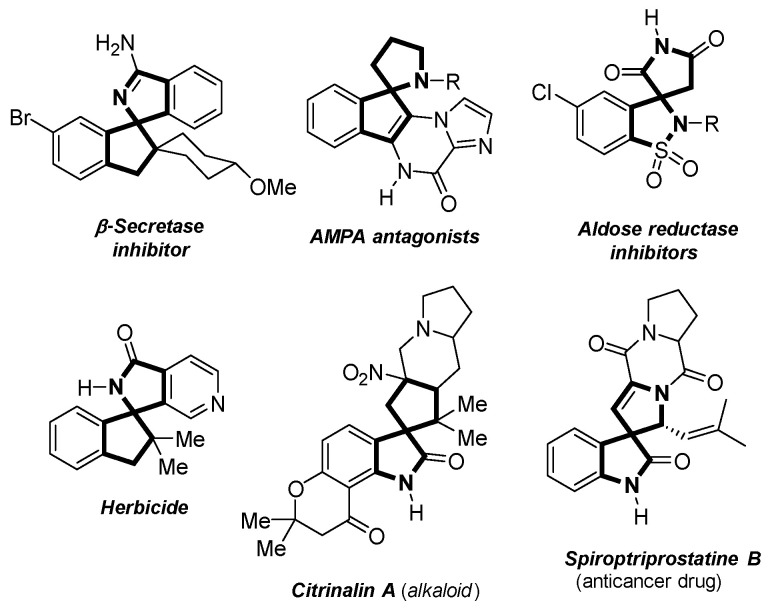
Selected examples of bioactive azaspiro[4,4]nonanes.

Nitrogen spirocyclic compounds constitute an important class of heterocycles with unique characteristics. The introduction of spiro moieties can profoundly alter the physicochemical and biological profiles of the parent compounds due to their high rigidity and unique three-dimensional geometries. In addition, spiralization has often been used as a reliable methodology to create more privileged structures in the drug discovery process [1,2]. Such heterocyclic spiro systems, in particular nitrogen spiro [4.4]nonanes, are widely represented in natural and synthetic biologically relevant molecules exhibiting important pharmacological and pesticidal properties [3,4]. They are able to function as *β*-secretase inhibitors [5], AMPA antagonists [6], aldose reductase inhibitors [7,8], herbicides [9], anticancer agents [10] (Figure 1), etc.

In the past decade, transition-metal-catalyzed, directing group (DG)-assisted C-H functionalization has emerged as one of the most efficient and straightforward strategies for the assembly of many valuable molecules in an atom-efficient and step-economical manner [11,12,13,14]. Following this methodology, the synthesis of various heterocyclic compounds can be readily achieved via a tandem CH-activation–annulation process from DG-equipped aromatic compounds using different alkynes as coupling partners. Herewith, Cp*Rh(III)-systems have been shown the most competent catalysts for these transformations [15,16,17,18,19,20,21,22]. However, the outcome of annulation rigorously depends on directing group architecture as well as the nature and location functional groups presented in the acetylene component. With the recent expansion of the directing group to the cyclic architecture, the synthesis of spiro compounds, including heterocyclic spiro[4,4]nonanes, has become possible [23].

On the top of this, fluorinated compounds have found widespread applications in life and material sciences. The incorporation of fluorine-containing functionalities into potential pharmaceuticals is a well-recognized synthetic tool used to adjust their steric, electronic, and biological properties [24]. In the field of amino acids and peptides, special attention is focused on α-amino acids with fluoromethyl groups in the α-position, owing to their ability to act as selective enzyme inhibitors, while exhibiting a range of interesting biological activities [25,26,27,28]. For these and the above reasons, the development of efficient synthetic approaches to new functionalized azaspiro-[4,4]nonane, in particular α-CF_3_-substituted spiroproline derivatives, is highly desirable.

**Scheme 1:**
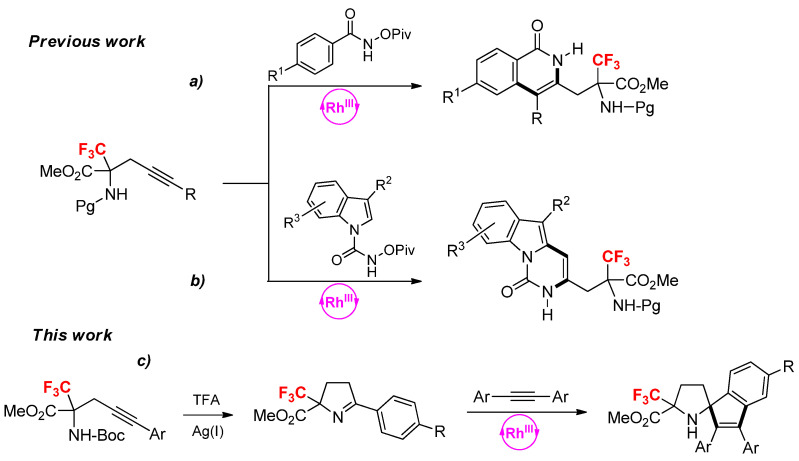
Previous and present work: (**a**) preparation of isoquinolones; (**b**) preparation of pirimidoindolones; (**c**) synthesis of dehydroprolines.

Recently, we have elaborated upon a convenient method for the preparation of novel *α*-CF_3_-substituted *α*-amino acid derivatives decorated with the pharmacophore isoquinolone [29,30] (Scheme 1a) and pirimidoindolone [31] (Scheme 1b) cores via Rh(III)-catalyzed C-H activation/(4+2)-annulation of the aryl hydroxamates and indole carboxamides with orthogonally protected propargyl-containing *α*-amino acid derivatives.

Now, in accordance with our current investigations focused on the development of efficient routes to new representatives of fluorinated α-amino acids and their multifunctional derivatives under metal catalysis [32,33,34], we want to report on a convenient region-selective approach to new CF_3_-containing spiro[indene-prolines]. The method involves the initial transformation of α-arylpropargyl-*α*-amino esters into corresponding 5-aryl-2-(trifluoromethyl)-3,4-dihydro-2*H*-pyrrole-2-carboxylates [35] followed by rhodium(III)-catalyzed C-H activation/[3+2]annulation with internal alkynes (Scheme 1c). The latter reaction, to the best of our knowledge, demonstrates the first example of aromatic C-H bond activation with the assistance of a dehydroproline directing group.

## 2. Results and Discussion

**Scheme 2:**
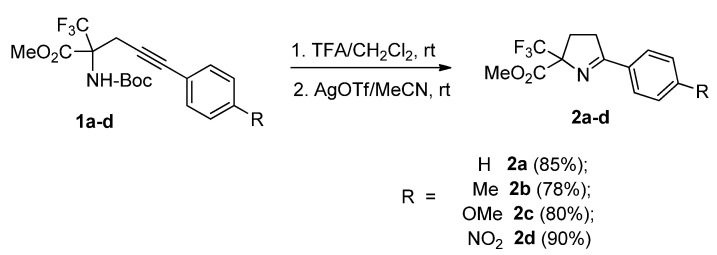
Synthesis of starting dehydroprolines **2**.

The synthesis of starting dehydroprolines **2a-d** was accomplished using a two-step procedure previously developed by us [35] from available arylpropargyl amino esters **1a-d** [36], including acid-mediated Boc-group deprotection followed by the silver(I)-catalyzed intramolecular hydroamination to afford the corresponding dehydroproline derivatives **2a-d** in high yields (Scheme 2).

In order to check the feasibility of the proline moiety of **2** to act as directing group in the*ortho*-metalation of the adjacent phenyl ring, we examined a model reaction between dehydroproline **2a** and tolane **3a** (Table 1).

First, we tested a [Cp*RhCl_2_]_2_/Ag(I) catalytic system that has demonstrated the best activity in many C-H activation reactions of (hetero)arenes with different coupling partners including acetylenes. When 5 mol% of the dimeric rhodium complex was combined with 30 mol % of chloride scavenger AgBF_4_, the desired spiro ring product, the corresponding 2,3-diphenyl-5′-(trifluoromethyl)spiro[indene-1,2′-pyrrolidine]-5′ -carboxylate (**4a**), was obtained in moderate yields (entry 1) after reaction at 80 °C in DCE, along with significant amounts of starting materials. The increase in the amount of silver additive to one equivalent did not improve the conversion of **2a** and the yield of **4a** (entries 2,3). However, the use of copper acetate as the second additive led to a better result (entry 4). The decrease in catalyst loading essentially diminished the yield of **4a** (entry 5). The further variation of additives, solvents (toluene, methanol), reaction temperature, and time revealed the following optimal conditions: the heating of 5-phenyl dehydroproline **2** with 1.1 equiv. of alkyne **3** at 80 °C in DCE in the presence of [Cp*RhCl_2_]_2_ (5 mol%), AgOTf (0.3 eqiuv.), Cu(OAc)_2_ (0.5 equiv.) for 16 h (entry 11). The reaction does not take place in the absence of silver additive (entry 14). Iridium- and cobalt-based complexes have proved to be absolutely inactive in the process (entries 15–16).

**Scheme 3:**
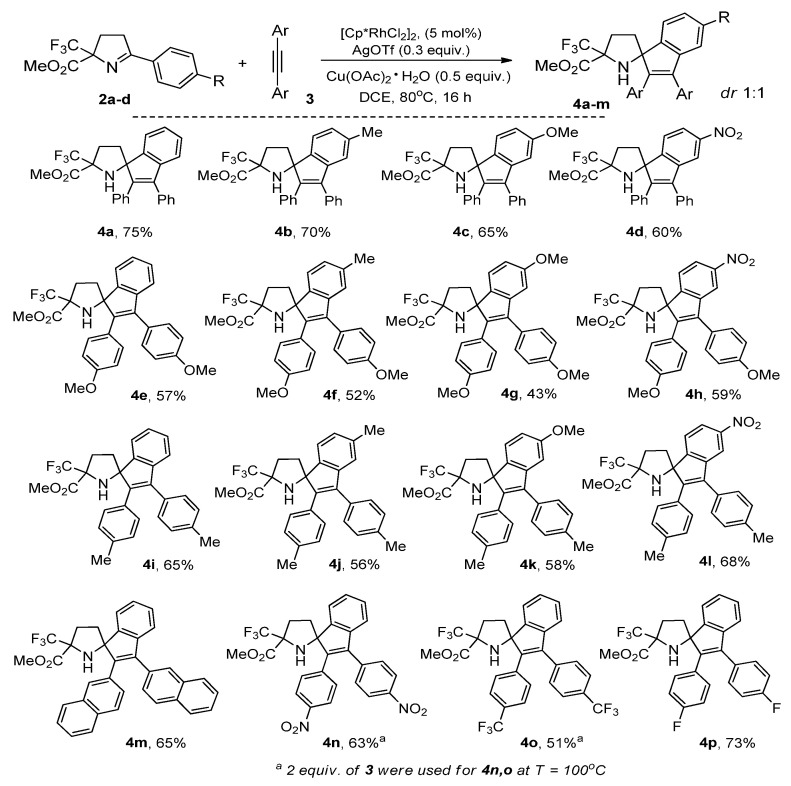
Synthesis of spiro prolines **4a–p**.

In the identified conditions, 5-aryl dehydroprolinates **2a-d** bearing different substituents in the phenyl ring were involved in C-H activation/[3+2]annulation with tolane derivatives **3**. As a result, a series of the corresponding CF_3_-containing spiro-[indene-prolinates] **4a-p** were synthesized in acceptable yields (Scheme 3). The nature of the substituents in both coupling components did not significantly affect the outcome of the reaction in most investigated cases. The exception was found only for the compounds **4n** and **4o**; thus, two-fold excess of alkyne component **3** and higher temperature (100 °C) were required to achieve the full conversion of starting dehydroproline **2a** for the same period of time.

**Figure 2:**
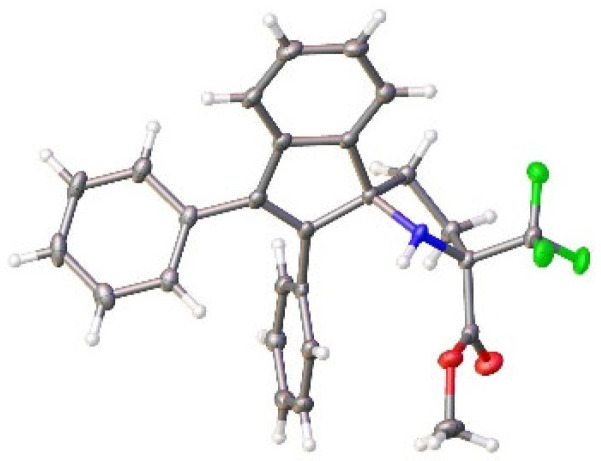
X-ray structure of **4a** (CCDC 2301938).

All synthesized compounds isolated in analytically pure form via flash chromatography were fully characterized by means of standard physicochemical methods (see Supplementary Materials). In addition, a single crystal of good quality was obtained from **4a** for X-ray analysis (Figure 2).

**Scheme 4:**
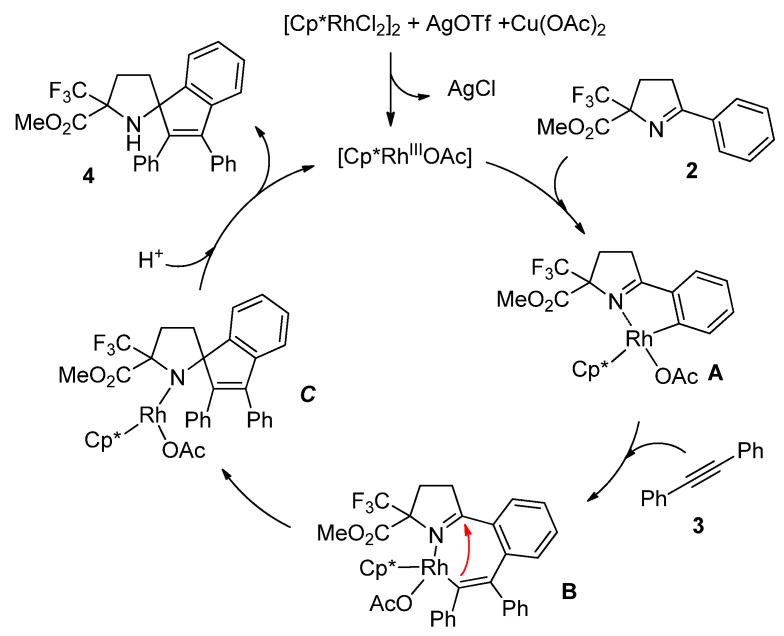
Proposed pathway of spiro cyclization.

Based on the literature precedents [23,37,38,39] and the results obtained above, the mechanism of the transformation outlined in Scheme 4 is proposed. Initially, the rhodium dimer complex [Cp*RhCl_2_]_2_ is easily converted into the catalytically active Cp*Rh^III^(OAc) species via dissociation and consecutive ligand exchange. Precomplexation to the directing dehydropirrolidine moiety is followed by the cleavage of *ortho*-C-H bond of phenyl ring to form rhodacyle ***A***. Then, the selective insertion of the alkyne triple bond into the C-Rh bond provides a seven-membered rhodacycle intermediate ***B***. Within intermediate ***B***, the addition of the vinyl C-Rh bond to the C=C double bond of the pyrrolidine ring leads to the intermediate **C**. Finally, the cleavage of the N-Rh bond of ***C*** through protonolysis produces **4,** and regenerates the active species for new catalytic cycle.

## 3. Materials and Methods

### 3.1. General Information

All solvents used in reactions were freshly distilled from appropriate drying agents before use. All other reagents were distilled as necessary. The corresponding starting dehydroprolines were easily synthesized via the previously described protocol [35,36]. Analytical TLC was performed with Merck silica gel 60 F 254 plates; visualization was accomplished with UV light or spraying with Ce(SO_4_)_2_ solution in 5% H_2_SO_4_. Chromatography was carried out using Merck silica gel (Kieselgel 60, 0.063–0.200 mm) and petroleum ether/ethyl acetate and petroleum ether/dichloromethane as an eluent. The NMR spectra were obtained with Bruker AV-300, AV-400, AV-500 and Inova-400 spectrometers operating at 300, 400, and 500 MHz, respectively, for ^1^H (TMS reference), at 101 and 126 for ^13^C {^1^H}, and at 282 and 376 MHz for ^19^F (CCl_3_F reference). Analytical data (C, H, N content) were obtained with a Carlo Erba model 1106 microanalyzer. High-resolution mass spectra were recorded on a Bruker Daltonics micrOTOF-Q II device (electrospray ionization).

### 3.2. General Procedure for the Synthesis of 5-Aryl Dehydroprolines ***2a**-**d***

To a solution of compound **1** [36] (0.3 g, 0.81 mmol, 1 equiv.) in dry CH_2_Cl_2_ (5 mL), TFA (2 mL) was added. The resulting mixture was stirred for 3–4 h at room temperature. The solvent and the excess of acid were removed under reduced pressure and the residue was dissolved in water (5 mL) and neutralized with sodium hydrogen carbonate until the pH reached 7. The product was extracted with ethyl acetate (2 × 10 mL), and the organic layer was dried over MgSO_4_. After removal of the solvent, the residue was dissolved in dry acetonitrile (3 mL), AgOTf (0.01 g, 0.04 mmol, 0.05 equiv.) was added, and the mixture was stirred for 4–5 h at room temperature. The solvent was removed under reduced pressure, and the corresponding product **2** was isolated via column chromatography on silica gel (eluent petroleum ether/ethyl acetate = 15:1). The spectral characteristics of the obtained compounds correspond to the literature data [35].

*Methyl 5-phenyl-2-(trifluoromethyl)-3,4-dihydro-2H-pyrrole-2-carboxylate* **2a**.

Yield 85% as a white solid. ^1^H NMR (300 MHz, CDCl_3_): *δ* 7.98 (d, *J* = 7.1 Hz, 2H, Ar), 7.60–7.48 (m, 3H, Ar), 3.89 (s, 3H, OCH_3_), 3.29 (t, *J* = 7.9 Hz, 2H, CH_2_), 2.72–2.62 (m, 1H, CH_2_), 2.58–2.48 (m, 1H, CH_2_). ^19^F NMR (282 MHz, CDCl_3_): *δ* −74.76 (s, 3F, CF_3_).

*Methyl 5-p-tolyl-2-(trifluoromethyl)-3,4-dihydro-2H-pyrrole-2-carboxylate* **2b**.

Yield 78% as a white solid. ^1^H NMR (300 MHz, CDCl_3_): *δ* 7.84 (d, *J* = 8.1 Hz, 2H, Ar), 7.26 (d, *J* = 8.1 Hz, 2H, Ar), 3.85 (s, 3H, OCH_3_), 3.22 (t, *J* = 7.9 Hz, 2H, CH_2_), 2.66–2.56 (m, 1H, CH_2_), 2.52–2.46 (m, 1H, CH_2_), 2.42 (s, 3H, CH_3_). ^19^F NMR (282 MHz, CDCl_3_): *δ* −74.79 (s, 3F, CF_3_).

*Methyl 5-(4-methoxyphenyl)-2-(trifluoromethyl)-3,4-dihydro-2H-pyrrole-2-carboxylate* **2c**.

Yield 80% as a white solid. ^1^H NMR (300 MHz, CDCl_3_): *δ* 7.90 (d, *J* = 8.8 Hz, 2H, Ar), 6.95 (d, *J* = 8.8 Hz, 2H, Ar), 3.88 (s, 3H, OCH_3_), 3.85 (s, 3H, OCH_3_), 3.20 (t, *J* = 7.9 Hz, 2H, CH_2_), 2.65–2.55 (m, 1H, CH_2_), 2.51–2.41 (m, 1H, CH_2_). ^19^F NMR (282 MHz, CDCl_3_): *δ* −74.82 (s, 3F, CF_3_).

*Methyl 5-(4-nitrophenyl)-2-(trifluoromethyl)-3,4-dihydro-2H-pyrrole-2-carboxylate* (**2d**).

Yield 90% as a yellow solid. ^1^H NMR (300 MHz, CDCl_3_): *δ* 8.32 (d, *J* = 8.8 Hz, 2H, Ar), 8.12 (d, *J* = 8.8 Hz, 2H, Ar), 3.88 (s, 3H, OCH_3_), 3.28 (t, *J* = 7.8 Hz, 2H, CH_2_), 2.74–2.64 (m, 1H, CH_2_), 2.60–2.50 (m, 1H, CH_2_). ^19^F NMR (282 MHz, CDCl_3_): *δ* −74.60 (s, 3F, CF_3_).

### 3.3. General Procedure for the C-H Activation/Annulation of 5-Aryl Dehydroprolines with Tolane Derivatives. Synthesis of the Compounds ***4a**-**m***

A 10 mL Schlenk tube equipped with a magnetic stirring bar was charged with a corresponding 5-aryl dehydroproline (0.1 g, 0.37 mmol, 1.0 equiv.), DCE (3 mL), a corresponding tolane derivative (0.07 g, 0.41 mmol, 1.1 equiv.), [Cp^*^RhCl_2_]_2_ (11.4 mg, 18 µmol, 5 mol%), AgOTf (28.4 mg, 0.11 mmol, 30 mol%), and Cu(OAc)_2_∙H_2_O (0.07 g, 0.37 mmol, 1.0 equiv.) under air. The reaction mixture was stirred at 80 °C for 16 h until the completion of the reaction, as monitored via TLC and ^19^F NMR. The heterogeneous mixture was passed through a short layer of celite, which was additionally washed with a CH_2_Cl_2_. After removal of the solvent, the residue was purified via column chromatography on silica gel (gradient elution petroleum ether/dichloromethane = 5:1, eluent petroleum ether/ethyl acetate = 10:1) to give the desired product.

*Methyl 2,3-diphenyl-5′-(trifluoromethyl)spiro[indene-1,2′-pyrrolidine]-5′-carboxylate* **4a**.

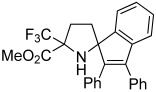



Yield: 75% as a white solid. M.p. 168 °C. ^1^H NMR (500 MHz, CDCl_3_): *δ* 7.61 (d, *J* = 7.05 Hz, 1H, Ar), 7.35–7.21 (m, 11H, Ar), 7.17–7.15 (m, 2H, Ar), 3.58 (s, 3H, OCH_3_), 3.10 (s, 1H, NH), 2.62–2.56 (m, 1H, CH_2_), 2.41–2.34 (m, 2H, CH_2_), 2.00–1.93 (m, 1H, CH_2_). ^13^C{^1^H} NMR (126 MHz, CDCl_3_): *δ* 169.6, 150.6, 148.4, 142.0, 139.3, 135.7, 134.1, 130.2, 129.2, 128.1, 128.0, 127.8, 127.4, 127.3, 126.8, 125.3 (q, *J* = 285.8 Hz, CF_3_), 122.5, 120.7, 77.6, 72.1 (q, *J* = 28.2 Hz, >C<), 53.0, 35.3, 30.5. ^19^F NMR (376 MHz, CDCl_3_): *δ* −77.33 (s, 3F, CF_3_). Elemental analysis calcd (%) for C_27_H_22_F_3_NO_2_: C, 72.15; H, 4.93; N, 3.12; found: C, 72.11; H, 5.04; N, 3.11. HRMS (ESI): calcd for C_27_H_23_F_3_NO_2_ [M+H]+: 450.1680; found: 477.1676.

*Methyl 5-methyl-2,3-diphenyl-5′-(trifluoromethyl)spiro[indene-1,2′-pyrrolidine]-5′-carboxylate* **4b**.

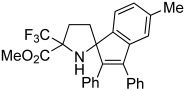



Yield: 70% as a white solid. M.p. 169–171 °C. ^1^H NMR (500 MHz, CDCl_3_): *δ* 7.52 (d, *J* = 7.5 Hz, 1H, Ar), 7.33–7.23 (m, 8H, Ar), 7.19–7.16 (m, 3H, Ar), 7.10 (s, 1H, Ar), 3.59 (s, 3H, OCH_3_), 3.11 (s, 1H, NH), 2.62–2.56 (m, 1H, CH_2_), 2.42–2.35 (m, 2H, CH_2_, 3H, CH_3_,), 2.02–1.95 (m, 1H, CH_2_). ^13^C{^1^H} NMR (126 MHz, CDCl_3_): *δ* 169.7, 148.7, 147.7, 142.3, 139.3, 137.7, 135.8, 134.3, 130.2, 129.2, 128.1, 127.4, 127.3, 127.2, 125.4 (q, *J* = 285.8 Hz, CF_3_), 122.3, 121.5, 77.4, 72.1 (q, *J* = 28.2 Hz, >C<), 53.0, 35.4, 30.6, 21.5. ^19^F NMR (376 MHz, CDCl_3_): *δ* −77.29 (s, 3F, CF_3_). Elemental analysis calcd (%) for C_28_H_24_F_3_NO_2_: C, 72.56; H, 5.22; N, 3.02; found: C, 72.67; H, 5.34; N, 2.91.

*Methyl 5-methoxy-2,3-diphenyl-5′-(trifluoromethyl)spiro[indene-1,2′-pyrrolidine]-5′-carboxylate* **4c**.

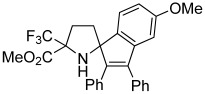



Yield: 65% as a white solid. M.p. 143–145 °C. ^1^H NMR (500 MHz, CDCl_3_): *δ* 7.52 (d, *J* = 8.1 Hz, 1H, Ar), 7.30–7.23 (m, 8H, Ar), 7.18–7.17 (m, 2H, Ar), 6.88–6.85 (m, 2H, Ar), 3.83 (s, 3H, OCH_3_), 3.59 (s, 3H, OCH_3_), 3.10 (s, 1H, NH), 2.60–2.56 (m, 1H, CH_2_), 2.40–2.34 (m, 2H, CH_2_), 2.00–1.93 (m, 1H, CH_2_). ^13^C{^1^H} NMR (126 MHz, CDCl_3_): *δ* 169.6, 160.0, 149.7, 143.6, 142.5, 138.9, 135.7, 134.0, 130.2, 129.2, 128.1, 127.4, 127.3, 125.4 (q, *J* = 286.0 Hz, CF_3_), 123.1, 111.5, 107.3, 77.1, 72.0 (q, *J* = 28.2 Hz, >C<), 55.6, 53.0, 35.4, 30.5. ^19^F NMR (282 MHz, CDCl_3_): *δ* −77.29 (s, 3F, CF_3_). Elemental analysis calcd (%) for C_28_H_24_F_3_NO_3_: C, 70.14; H, 5.05; N, 2.92; found: C, 70.07; H, 5.29; N, 2.84.

*Methyl 5-nitro-2,3-diphenyl-5′-(trifluoromethyl)spiro[indene-1,2′-pyrrolidine]-5′-carboxylate* **4d**.

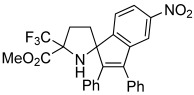



Yield: 60% as a yellow solid. M.p. 188 °C. ^1^H NMR (500 MHz, CDCl_3_): *δ* 8.21 (dd, *J* = 8.2, 2.05 Hz, 1H, Ar), 8.06 (d, *J* = 2.0 Hz, 1H, Ar), 7.71 (d, *J* = 8.2 Hz, 1H, Ar), 7.32–7.28 (m, 6H, Ar), 7.25–7.23 (m, 2H, Ar), 7.17–7.15 (m, 2H, Ar), 3.59 (s, 3H, OCH_3_), 3.16 (s, 1H, NH), 2.63–2.57 (m, 1H, CH_2_), 2.41–2.36 (m, 2H, CH_2_), 1.98–1.91 (m, 1H, CH_2_). ^13^C{^1^H} NMR (126 MHz, CDCl_3_): *δ* 169.2, 157.7, 150.8, 148.5, 143.6, 137.9, 134.6, 132.7, 129.9, 128.9, 128.5, 128.4, 128.0, 127.9, 125.2 (q, *J* = 285.6 Hz, CF_3_), 123.0, 122.6, 115.5, 77.4, 72.1 (q, *J* = 28.4 Hz, >C<), 53.2, 35.4, 30.3. ^19^F NMR (282 MHz, CDCl_3_): *δ* −77.38 (s, 3F, CF_3_). Elemental analysis calcd (%) for C_27_H_21_F_3_N_2_O_4_: C, 65.58; H, 4.28; N, 5.67; found: C, 65.74; H, 4.29; N, 5.41.

*Methyl 2,3-bis(4-methoxyphenyl)-5′-(trifluoromethyl)spiro[indene-1,2′-pyrrolidine]-5′-carboxylate* **4e**.

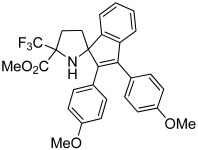



Yield: 57% as a yellow solid. M.p. 146–148 °C. ^1^H NMR (500 MHz, CDCl_3_): *δ* 7.60 (d, *J* = 6.5 Hz, 1H Ar,), 7.33–7.27 (m, 3H, Ar), 7.21–7.18 (m, 2H, Ar), 7.09–7.06 (m, 2H, Ar), 6.85–6.80 (m, 4H, Ar), 3.81 (s, 3H, OCH_3_), 3.79 (s, 3H, OCH_3_), 3.61 (s, 3H, OCH_3_), 3.07 (s, 1H, NH), 2.60–2.53 (m, 1H, CH_2_), 2.42–2.37 (m, 1H, CH_2_), 2.35–2.30 (m, 1H, CH_2_), 2.06–2.00 (m, 1H, CH_2_). ^13^C{^1^H} NMR (126 MHz, CDCl_3_): *δ* 169.7, 158.8, 158.6, 150.7, 147.3, 142.2, 138.8, 131.4, 130.4, 127.9, 127.7, 126.6, 126.5, 125.4 (q, *J* = 285.7 Hz, CF_3_), 122.5, 120.6, 113.7, 113.6, 77.4, 72.1 (q, *J* = 28.3 Hz, >C<), 55.2, 55.1, 53.0, 35.3, 30.5. ^19^F NMR (282 MHz, CDCl_3_): *δ* −77.20 (s, 3F, CF_3_). Elemental analysis calcd (%) for C_29_H_26_F_3_NO_4_: C, 68.36; H, 5.14; N, 2.75; found: C, 68.51; H, 5.24; N, 2.91.

*Methyl 2,3-bis(4-methoxyphenyl)-5-methyl-5*′*-(trifluoromethyl)spiro[indene-1,2′-pyrrolidine]-5′-carboxylate* **4f**.

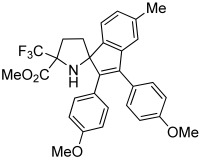



Yield: 52% as a yellow solid. M.p. 180–182 °C. ^1^H NMR (400 MHz, CDCl_3_): *δ* 7.42 (d, *J* = 7.7 Hz, 1H, Ar), 7.14 (d, *J* = 8.6 Hz, 2H, Ar), 7.08 (d, *J* = 7.4 Hz, 1H, Ar), 7.03 (s, 2H, Ar), 7.00 (s, 1H, Ar), 6.78 (d, *J* = 8.6 Hz, 4H, Ar), 3.76 (s, 3H, OCH_3_), 3.75 (s, 3H, OCH_3_), 3.56 (s, 3H, OCH_3_), 2.99 (s, 1H, NH), 2.52–2.44 (m, 1H, CH_2_), 2.36–2.23 (m, 5H, CH_3_, CH_2_), 2.00–1.92 (m, 1H, CH_2_). ^13^C{^1^H} NMR (101 MHz, CDCl_3_): *δ* 169.7, 158.8, 158.5, 147.8, 147.6, 142.4, 138.8, 137.5, 131.3, 130.3, 127.9, 127.1, 126.6, 125.3 (q, *J* = 286.7 Hz, CF_3_), 122.1, 121.3, 113.6, 113.5, 77.1, 72.0 (q, *J* = 28.3 Hz, >C<), 55.1, 55.0, 52.9, 35.3, 30.5, 21.5. ^19^F NMR (282 MHz, CDCl_3_): *δ* −77.17 (s, 3F, CF_3_). Elemental analysis calcd (%) for C_30_H_28_F_3_NO_4_: C, 68.82; H, 5.39; N, 2.68; found: C, 68.69; H, 5.64; N, 2.54.

*Methyl 5-methoxy-2,3-bis(4-methoxyphenyl)-5′-(trifluoromethyl)spiro[indene-1,2′-pyrrolidine]-5′-carboxylate* **4g**.

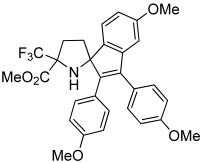



Yield: 43% as a yellow solid. M.p. 154–156 °C. ^1^H NMR (400 MHz, CDCl_3_): *δ* 7.43 (d, *J* = 8.9 Hz, 1H, Ar), 7.13 (d, *J* = 8.8 Hz, 2H, Ar), 7.02 (d, *J* = 8.6 Hz, 2H, Ar), 6.79–6.75 (m, 6H, Ar), 3.78 (s, 3H, OCH_3_), 3.76 (s, 3H, OCH_3_), 3.75 (s, 3H, OCH_3_), 3.56 (s, 3H, OCH_3_), 2.99 (s, 1H, NH), 2.51–2.43 (m, 1H, CH_2_), 2.35–2.22 (m, 2H, CH_2_), 1.99–1.91 (m, 1H, CH_2_). ^13^C{^1^H} NMR (101 MHz, CDCl_3_): *δ* 169.6, 159.9, 158.8, 158.6, 148.6, 143.8, 142.6, 138.5, 132.8, 131.3, 130.3, 130.0, 127.9, 126.4, 125.3 (q, *J* = 286.5 Hz, CF_3_), 122.9, 113.6, 113.5, 111.2, 107.2, 76.8, 71.9 (q, *J* = 28.3 Hz, >C<), 55.6, 55.1, 55.0, 52.9, 35.3, 30.5. ^19^F NMR (282 MHz, CDCl_3_): *δ* −77.19 (s, 3F, CF_3_). Elemental analysis calcd (%) for C_30_H_28_F_3_NO_5_: C, 66.78; H, 5.23; N, 2.60; found: C, 66.48; H, 5.41; N, 2.63.

*Methyl 2,3-bis(4-methoxyphenyl)-5-nitro-5′-(trifluoromethyl)spiro[indene-1,2′-pyrrolidine]-5′-carboxylate* **4h**.

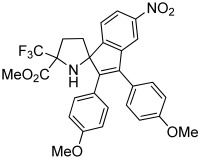



Yield: 59% as a yellow solid. M.p.118–120 °C. ^1^H NMR (400 MHz, CDCl_3_): *δ* 8.15 (dd, *J* = 8.2, 2.1 Hz, 1H, Ar), 8.02 (d, *J* = 2.0 Hz, 1H, Ar), 7.65 (d, *J* = 8.2 Hz, 1H, Ar), 7.13 (d, *J* = 8.8 Hz, 2H, Ar), 7.02 (d, *J* = 8.7 Hz, 2H, Ar), 6.81 (d, *J* = 8.8 Hz, 4H, Ar), 3.77 (s, 3H, OCH_3_), 3.77 (s, 3H, OCH_3_), 3.59 (s, 1H, OCH_3_), 3.07 (s, 1H, NH), 2.56–2.48 (m, 1H, CH_2_), 2.38–2.26 (m, 2H, CH_2_), 2.00–1.91 (m, 1H, CH_2_). ^13^C{^1^H} NMR (101 MHz, CDCl_3_): *δ* 169.3, 159.2, 159.1, 157.8, 149.7, 148.4, 143.8, 137.5, 131.0, 130.1, 126.7, 125.2 (q, *J* = 286.3 Hz, CF_3_), 125.1, 122.9, 122.3, 115.3, 113.9, 113.8, 77.1, 72.1 (q, *J* = 28.3 Hz, >C<), 55.2, 53.1, 35.3, 30.3. ^19^F NMR (282 MHz, CDCl_3_): *δ* −77.29 (s, 3F, CF_3_). Elemental analysis calcd (%) for C_29_H_25_F_3_N_2_O_6_: C, 62.81; H, 4.54; N, 5.05; found: C, 63.11; H, 4.86; N, 4.97.

*Methyl 2,3-di-p-tolyl-5′-(trifluoromethyl)spiro[indene-1,2′-pyrrolidine]-5′-carboxylate* **4i**.

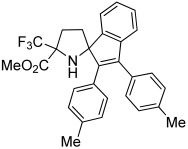



Yield: 65% as a white solid. M.p. 159–160 °C. ^1^H NMR (500 MHz, CDCl_3_): *δ* 7.60 (d, *J* = 6.8 Hz, 1H, Ar), 7.33–7.26 (m, 3H, Ar), 7.16 (d, *J* = 8.0 Hz, 2H, Ar), 7.11–7.07 (m, 4H, Ar), 7.04 (d, *J* = 8.0 Hz, 2H, Ar), 3.58 (s, 3H, OCH_3_), 3.07 (s, 1H, NH), 2.59–2.53 (m, 1H, CH_2_), 2.40–2.36 (m, 2H, CH_2_), 2.35 (s, 3H, CH_3_), 2.32 (s, 3H, CH_3_), 2.03–1.97 (m, 1H, CH_2_). ^13^C{^1^H} NMR (126 MHz, CDCl_3_): *δ* 169.6, 150.7, 147.9, 142.2, 138.9, 136.8, 132.6, 131.2, 130.0, 129.0, 128.9, 128.8, 127.7, 126.6, 125.4 (q, *J* = 285.4 Hz, CF_3_), 122.4, 120.6, 77.5, 72.1 (q, *J* = 28.1 Hz, >C<), 52.9, 35.3, 30.5, 21.3, 21.2. ^19^F NMR (282 MHz, CDCl_3_): *δ* -77.30 (s, 3F, CF_3_). Elemental analysis calcd (%) for C_29_H_26_F_3_NO_2_: C, 72.94; H, 5.49; N, 2.93; found: C, 72.74; H, 5.65; N, 2.88.

*Methyl 5-methyl-2,3-di-p-tolyl-5′-(trifluoromethyl)spiro[indene-1,2′-pyrrolidine]-5′-carboxylate* **4j**.

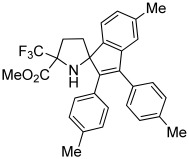



Yield: 56% as a white solid. M.p. 189–190 °C. ^1^H NMR (500 MHz, CDCl_3_): *δ* 7.47 (d, *J* = 7.5 Hz, 1H, Ar), 7.15 (d, *J* = 8.1 Hz, 2H, Ar), 7.12 (d, *J* = 7.6 Hz, 1H, Ar), 7.10–7.07 (m, 5H, Ar), 7.03 (d, *J* = 8.0 Hz, 2H, Ar), 3.57 (s, 3H, OCH_3_), 3.04 (s, 1H, NH), 2.56–2.47 (m, 1H, CH_2_), 2.39–2.36 (m, 1H, CH_2_), 2.37 (s, 3H, CH_3_), 2.34 (s, 3H, CH_3_), 2.33 (s, 3H, CH_3_), 2.33–2.29 (m, 1H, CH_2_), 2.02–1.95 (m, 1H, CH_2_). ^13^C{^1^H} NMR (126 MHz, CDCl_3_): *δ* 169.6, 148.2, 147.7, 142.4, 138.9, 137.5, 136.7, 132.7, 131.3, 130.0, 129.0, 128.8, 127.1, 125.4 (q, *J* = 285.4 Hz, CF_3_), 122.1, 121.4, 77.2, 72.1 (q, *J* = 28.1 Hz, >C<), 52.8, 35.3, 30.5, 21.5, 21.3, 21.2. ^19^F NMR (282 MHz, CDCl_3_): *δ* −77.28 (s, 3F, CF_3_). Elemental analysis calcd (%) for C_30_H_28_F_3_NO_2_: C, 73.30; H, 5.74; N, 2.85; found: C, 73.33; H, 5.93; N, 2.88.

*Methyl 5-metoxy-2,3-di-p-tolyl-5′-(trifluoromethyl)spiro[indene-1,2′-pyrrolidine]-5′-carboxylate* **4k**.

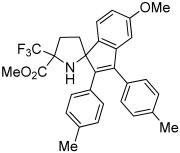



Yield: 58% as a white solid. M.p. 183–184 °C. ^1^H NMR (500 MHz, CDCl_3_): *δ* 7.48 (d, *J* = 8.2 Hz, 1H, Ar), 7.15 (d, *J* = 8.1 Hz, 2H, Ar), 7.10–7.07 (m, 4H, Ar), 7.03 (d, *J* = 8.0 Hz, 2H, Ar), 6.85–6.83 (m, 2H, Ar), 3.81 (s, 3H, OCH_3_), 3.57 (s, 3H, OCH_3_), 3.04 (s, 1H, NH), 2.55–2.47 (m, 1H, CH_2_), 2.38–2.33 (m, 1H, CH_2_, 3H, CH_3_), 2.32–2.29 (m, 3H, CH_3_, 1H, CH_2_), 2.02–1.95 (m, 1H, CH_2_). ^13^C{^1^H} NMR (126 MHz, CDCl_3_): *δ* 169.6, 159.9, 149.2, 143.7, 142.6, 138.6, 136.8, 132.6, 131.1, 129.9, 129.0, 128.8, 128.8, 125.4 (q, *J* = 285.3 Hz, CF_3_), 122.9, 111.3, 107.2, 77.0, 71.9 (q, *J* = 28.1 Hz, >C<), 55.6, 52.8, 35.2, 30.5, 21.3, 21.2. ^19^F NMR (282 MHz, CDCl_3_): *δ* −77.31 (s, 3F, CF_3_). Elemental analysis calcd (%) for C_30_H_28_F_3_NO_3_: C, 70.99; H, 5.56; N, 2.76; found: C, 70.74; H, 5.62; N, 2.81.

*Methyl 5-nitro-2,3-di-p-tolyl-5′-(trifluoromethyl)spiro[indene-1,2′-pyrrolidine]-5′-carboxylate* **4l**.

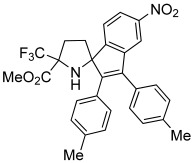



Yield: 68% as a yellow solid. M.p. 171–172 °C. ^1^H NMR (500 MHz, CDCl_3_): *δ* 8.19 (dd, *J* = 8.2, 2.1 Hz, 1H, Ar), 8.05 (d, *J* = 2.1 Hz, 1H, Ar), 7.69 (d, *J* = 8.5 Hz, 1H, Ar), 7.15–7.11 (m, 6H, Ar), 7.03 (d, *J* = 8.0 Hz, 2H, Ar), 3.59 (s, 3H, OCH_3_), 3.12 (s, 1H, NH), 2.60–2.54 (m, 1H, CH_2_), 2.40–2.35 (m, 2H, CH_2_), 2.35 (s, 3H, CH_3_), 2.34 (s, 3H, CH_3_), 2.01–1.95 (m, 1H, CH_2_). ^13^C{^1^H} NMR (126 MHz, CDCl_3_): *δ* 169.2, 157.8, 150.3, 148.4, 143.8, 137.6, 137.5, 131.5, 129.8, 129.7, 129.2, 129.0, 128.8, 125.2 (q, *J* = 285.2 Hz, CF_3_), 122.9, 122.3, 115.4, 77.3, 72.1 (q, *J* = 28.6 Hz, >C<), 53.0, 35.3, 30.3, 21.3, 21.2. ^19^F NMR (282 MHz, CDCl_3_): *δ* −77.40 (s, 3F, CF_3_). Elemental analysis calcd (%) for C_29_H_25_F_3_N_2_O_4_: C, 66.66; H, 4.82; N, 5.36; found: C, 66.51; H, 4.94; N, 5.41.

*Methyl 2,3-di(naphthalen-2-yl)-5′-(trifluoromethyl)spiro[indene-1,2′-pyrrolidine]-5′-carboxylate* **4m**.

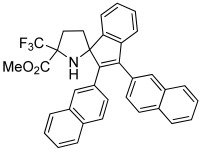



Yield: 65% as a white solid. M.p. 184 °C. ^1^H NMR (500 MHz, CDCl_3_): *δ* 7.88 (s, 1H, Ar), 7.83–7.79 (m, 2H, Ar), 7.77–7.72 (m, 4H, Ar), 7.69–7.65 (m, 2H, Ar), 7.50–7.46 (m, 2H, Ar), 7.45–7.42 (m, 2H, Ar), 7.40–7.32 (m, 4H, Ar), 7.31–7.29 (m, 1H, Ar), 3.24 (s, 1H, NH), 3.15 (s, 3H, OCH_3_), 2.71–2.65 (m, 1H, CH_2_), 2.50–2.46 (m, 1H, CH_2_), 2.38–2.33 (m, 1H, CH_2_), 1.95–1.88 (m, 1H, CH_2_). ^13^C{^1^H} NMR (126 MHz, CDCl_3_): *δ* 169.6, 150.7, 148.8, 142.1, 139.4, 133.2, 133.1, 133.0, 132.5, 132.4, 131.7, 129.4, 128.2, 128.1, 128.1, 128.0, 127.9, 127.8, 127.7, 127.6, 127.6, 127.3, 126.9, 126.2, 126.1, 126.0, 125.4 (q, *J* = 286.0 Hz, CF_3_), 122.7, 120.8, 78.0, 72.1 (q, *J* = 28.0 Hz, >C<), 52.7, 35.7, 30.5. ^19^F NMR (282 MHz, CDCl_3_): *δ* −77.31 (s, 3F, CF_3_). Elemental analysis calcd (%) for C_35_H_26_F_3_NO_2_: C, 76.49; H, 4.77; N, 2.55; found: C, 76.41; H, 5.17; N, 2.50.

*Methyl 2,3-bis(4-nitrophenyl)-5′-(trifluoromethyl)spiro[indene-1,2′-pyrrolidine]-5′-carboxylate* **4n**.

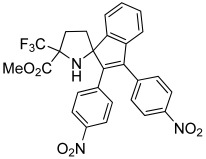



Yield: 63% as a yellow solid. M.p. 223–224 °C. ^1^H NMR (500 MHz, CDCl_3_): *δ* 8.16 (dd, *J* = 8.7, 3.2 Hz, 4H, Ar), 7.64 (d, *J* = 7.4 Hz, 1H, Ar), 7.41–7.33 (m, 6H, Ar), 7.19 (d, *J* = 7.4 Hz, 1H, Ar), 3.65 (s, 3H, OCH_3_), 3.08 (s, 1H, NH), 2.68–2.62 (m, 1H, CH_2_), 2.45–2.40 (m, 1H, CH_2_), 2.31–2.29 (m, 1H, CH_2_), 1.87–1.81 (m, 1H, CH_2_). ^13^C{^1^H} NMR (126 MHz, CDCl_3_): *δ* 169.6, 149.9, 148.6, 147.5, 147.2, 142.3, 140.3, 140.2, 138.9, 131.1, 129.9, 128.4, 128.0, 125.1 (q, *J* = 285.4 Hz, CF_3_), 123.8, 123.5, 122.9, 120.7, 78.0, 71.9 (q, *J* = 28.4 Hz, >C<), 53.3, 35.5, 30.4. ^19^F NMR (282 MHz, CDCl_3_): *δ* −76.89 (s, 3F, CF_3_). Elemental analysis calcd (%) for C_27_H_20_F_3_N_3_O_6_: C, 60.11; H, 3.74; N, 7.79; found: C, 60.24; H, 3.77; N, 7.72.

*Methyl 5′-(trifluoromethyl)-2,3-bis(4-(trifluoromethyl)phenyl)spiro[indene-1,2′-pyrrolidine]-5′-carboxylate* **4o**.

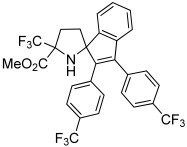



Yield: 51% as a yellow solid. M.p. 144–146 °C. ^1^H NMR (300 MHz, CDCl_3_): *δ* 7.63–7.54 (m, 5H, Ar), 7.40–7.28 (m, 6H, Ar), 7.20 (d, *J* = 6.8 Hz, 1H, Ar), 3.56 (s, 3H, OCH_3_), 3.07 (s, 1H, NH), 2.66–2.58 (m, 1H, CH_2_), 2.44–2.30 (m, 2H, CH_2_), 1.88–1.84 (m, 1H, CH_2_). ^13^C{^1^H} NMR (126 MHz, CDCl_3_): *δ* 169.7, 150.2, 148.3, 140.9, 139.2, 138.9, 137.4, 130.6, 129.7 (q, *J* = 32.8 Hz), 129.4, 128.2, 127.6, 125.4 (q, *J* = 3.7 Hz, >C<), 125.2 (q, *J* = 3.7 Hz, >C<), 125.2 (q, *J* = 285.9 Hz, CF_3_), 124.0 (q, *J* = 272.8 Hz, CF_3_), 123.9 (q, *J* = 272.4 Hz, CF_3_), 122.8, 120.7, 77.9, 71.9 (q, *J* = 28.4 Hz, >C<), 53.0, 35.5, 30.4. ^19^F NMR (282 MHz, CDCl_3_): *δ* −62.61 (s, 3F, CF_3_), -62.62 (s, 3F, CF_3_), −77.27 (s, 3F, CF_3_). Elemental analysis calcd (%) for C_29_H_20_F_9_NO_2_: C, 59.49; H, 3.44; N, 2.39; found: C, 59.35; H, 3.58; N, 2.55.

*Methyl 2,3-bis(4-fluorophenyl)-5′-(trifluoromethyl)spiro[indene-1,2′-pyrrolidine]-5′-carboxylate* **4p**.

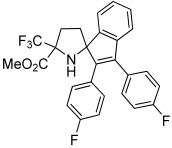



Yield: 73% as a yellow solid. M.p. 155–157 °C. ^1^H NMR (300 MHz, CDCl_3_): *δ* 7.60 (d, *J* = 6.6 Hz, 1H, Ar), 7.34–7.28 (m, 2H, Ar), 7.22–7.17 (m, 3H, Ar), 7.15–7.10 (m, 2H, Ar), 7.03–6.94 (m, 4H, Ar), 3.62 (s, 3H, OCH_3_), 3.05 (s, 1H, NH), 2.64–2.53 (m, 1H, CH_2_), 2.44–2.27 (m, 2H, CH_2_), 2.00–1.89 (m, 1H, CH_2_). ^13^C{^1^H} NMR (126 MHz, CDCl_3_): *δ* 169.7, 162.2 (d, *J* = 248.2 Hz), 161.9 (d, *J* = 246.9), 150.4, 147.5, 141.6, 138.9, 131.9 (d, *J* = 7.6 Hz), 131.4 (d, *J* = 3.8 Hz), 130.8 (d, *J* = 7.6 Hz), 129.8 (d, *J* = 3.8 Hz), 127.9, 127.1, 125.3 (q, *J* = 285.9 Hz, CF_3_), 122.6, 120.5, 115.4 (d, *J* = 3.7 Hz), 115.2 (d, *J* = 5.0 Hz), 77.4, 72.0 (q, *J* = 28.3 Hz, >C<), 53.1, 35.3, 30.5. ^19^F NMR (282 MHz, CDCl_3_): *δ* −77.16 (s, 3F, CF_3_), −113.98 (s, 1F, F), −114.08 (s, 1F, F). Elemental analysis calcd (%) for C_27_H_20_F_5_NO_2_: C, 66.80; H, 4.15; N, 2.89; found: C, 67.03; H, 4.23; N, 3.01.

## 4. Conclusions

In summary, we have presented a convenient and highly efficient method of accessing CF_3_-containing spiro-[indene-proline] derivatives from readily available precursors under mild catalytic conditions. An important feature of the Cp*Rh(III)-catalyzed tandem C-H activation/[3+2]–annulation of 5-aryl-2-(trifluoromethyl)-3,4-dihydro-2*H*-pyrrole- 2-carboxylates with acetylenes is the feasibility of the dehydroproline moiety to function as a directing group in this spiro annulation process. As a result, the developed strategy opens the door to a novel series of α-trifluoromethyl-substituted spiro-proline derivatives in good yields.

## Figures and Tables

**Table 1:**
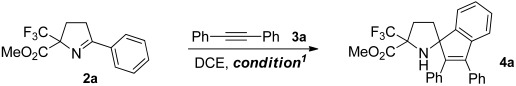
Optimization of [3+2]-annulation of 5-phenyl dehydroproline **2a** with acetylene **3a** ^1^.

Entry	Catalyst (mol%)	Ag Additive (Equiv.)	Other Additive (Equiv.)	Yield ^2^ (%)
1	[Cp*RhCl_2_]_2_ (5)	AgBF_4_ (0.3)	-	41(28 ^3^)
2	[Cp*RhCl_2_]_2_ (5)	AgBF_4_ (1)	-	39
3	[Cp*RhCl_2_]_2_ (5)	AgOTf (1)	-	43
4	[Cp*RhCl_2_]_2_ (5)	AgOTf (1)	Cu(OAc)_2_∙H_2_O (1)	71
5	[Cp*RhCl_2_]_2_ (2)	AgOTf (1)	Cu(OAc)_2_∙H_2_O (1)	42
6	[Cp*RhCl_2_]_2_ (5)	AgOTf (1)	Cu(OAc)_2_∙H_2_O (2)	63
7	[Cp*RhCl_2_]_2_ (5)	AgOAc (0.3)	Cu(OAc)_2_∙H_2_O (1)	NR
8	[Cp*RhCl_2_]_2_ (5)	AgOTf (0.3)	Cu(OAc)_2_∙H_2_O (1)	80
9	[Cp*RhCl_2_]_2_ (5)	AgBF_4_ (0.3)	Cu(OAc)_2_∙H_2_O (1)	77
10	[Cp*RhCl_2_]_2_ (5)	AgSbF_6_ (0.3)	Cu(OAc)_2_∙H_2_O (1)	79
**11**	[Cp*RhCl_2_]_2_ (5)	**AgOTf (0.3)**	**Cu(OAc)_2_∙H_2_O (0.5)**	**88(75 ^3^)**
12	[Cp*RhCl_2_]_2_ (5)	AgOTf (0.3)	CsOAc (2)	19
13	[Cp*RhCl_2_]_2_ (5)	AgOTf (0.3)	Zn(OAc)_2_∙H_2_O (2)	56
14	[Cp*RhCl_2_]_2_ (5)	-	Cu(OAc)_2_∙H_2_O (1)	NR
15	[Cp*IrCl_2_]_2_ (7)	AgOTf (1)	Cu(OAc)_2_∙H_2_O (1)	NR
16	[Cp*CoI_2_]_2_ (10)	AgOTf (1)	Cu(OAc)_2_∙H_2_O (1)	NR

^1^ *Reagents and conditions*: dehydroproline **2a** (0.2 mmol), acetylene **3a** (0.22 mmol), DCE (2 mL), 80 °C, 16 h; ^2^ Determined via ^19^F NMR spectroscopy; ^3^ Isolated yield.

## Data Availability

Data are contained within the article and Supplementary Materials.

## Supplementary Materials

The following supporting information can be downloaded at: https://www.mdpi.com/article/10.3390/molecules28237809/s1. The following are available online: copies of ^1^H and ^13^C NMR spectra for all novel compounds.
