# Supplementary material for: Synthesis of CF3-Containing Spiro-[Indene-Proline] Derivatives via Rh(III)-Catalyzed C-H Activation/Annulation
Source: Molecules. 2023 Nov 27;28(23):7809. doi: 10.3390/molecules28237809 (PMC10708072; doi:10.3390/molecules28237809)
Supplement: Supplementary file 1 [file molecules-28-07809-s001.zip › molecules-2711769-supplementary.pdf]

Table of contents

1. ^{1}H, ^{19}F and ^{13}C NMR Spectra of Compounds S2-S49
2. HPLC-HRMS data for **4a** S50

^{1}H NMR, ^{19}F NMR and ^{13}C NMR Spectra.

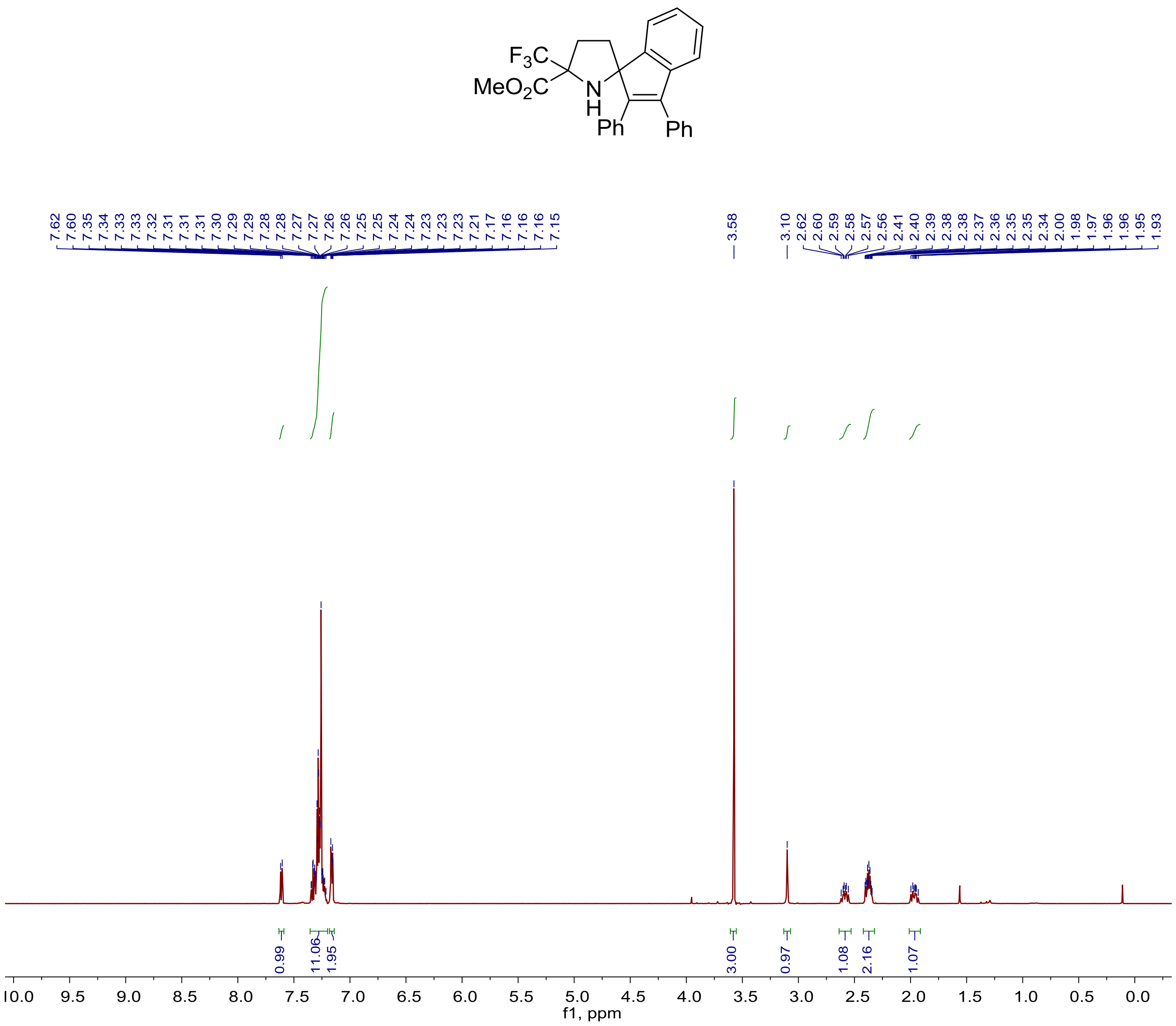

^{1}H spectrum of **4a** in $CDCl_3$

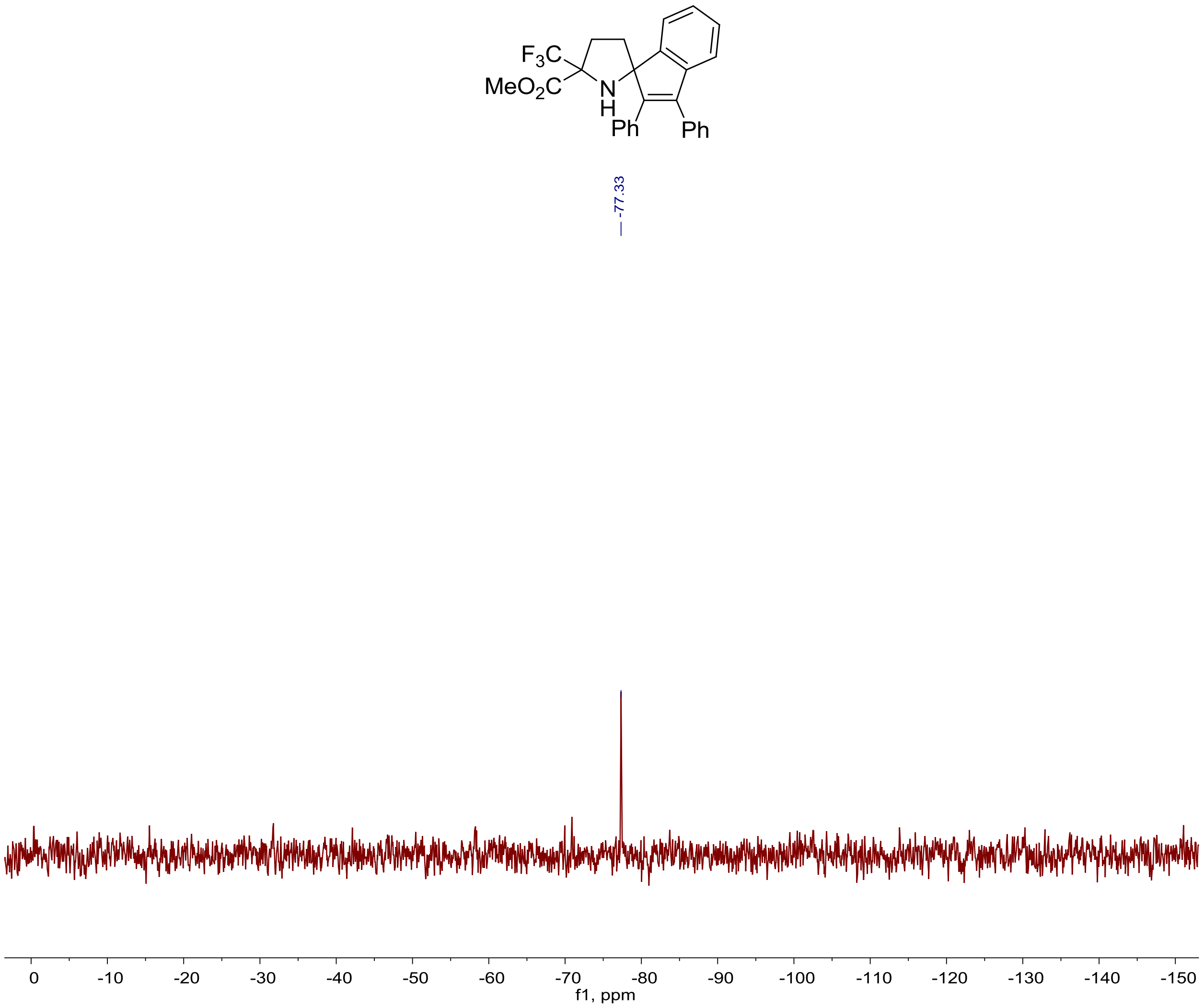

^{19}F spectrum of **4a** in $CDCl_3$

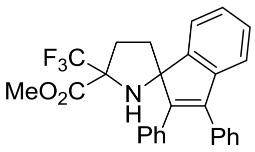

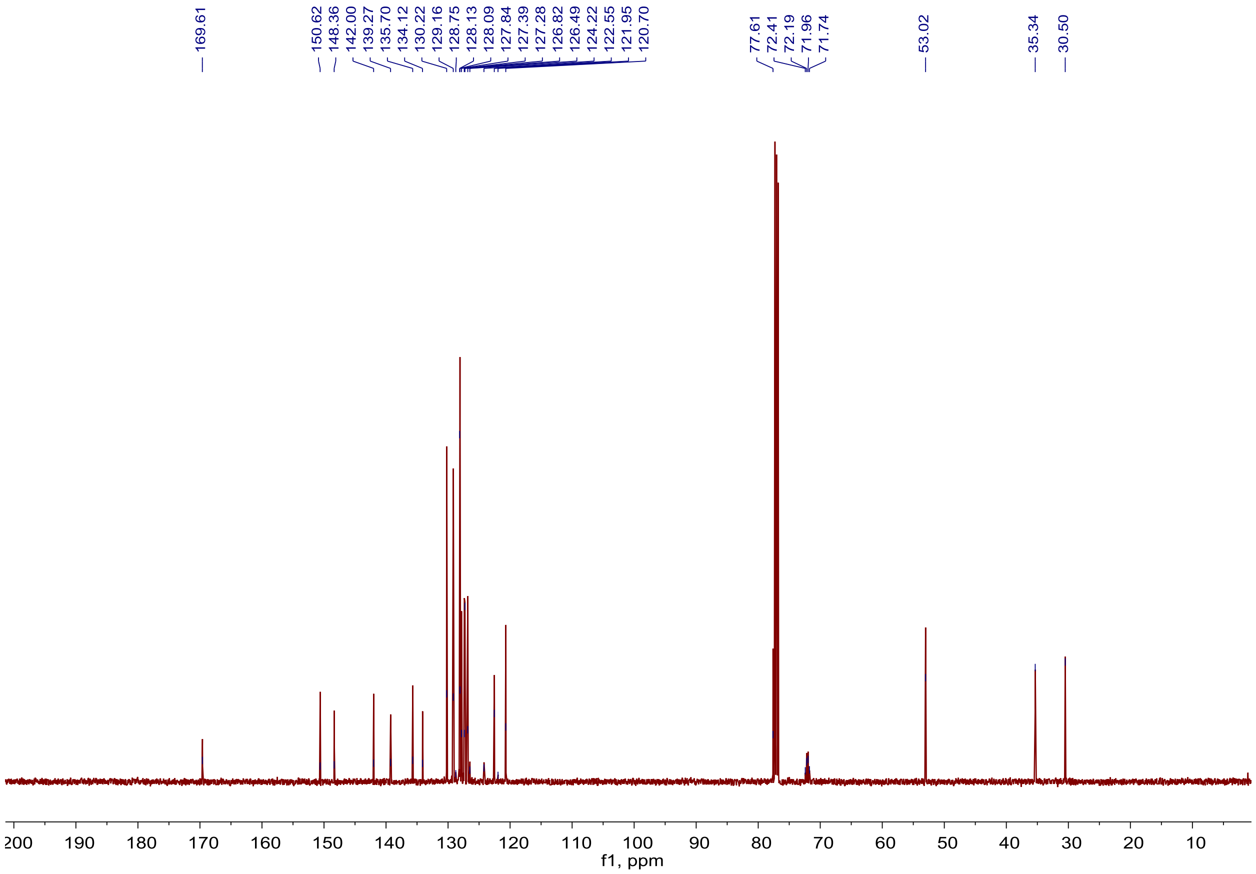

^{13}C spectrum of **4a** in $CDCl_3$

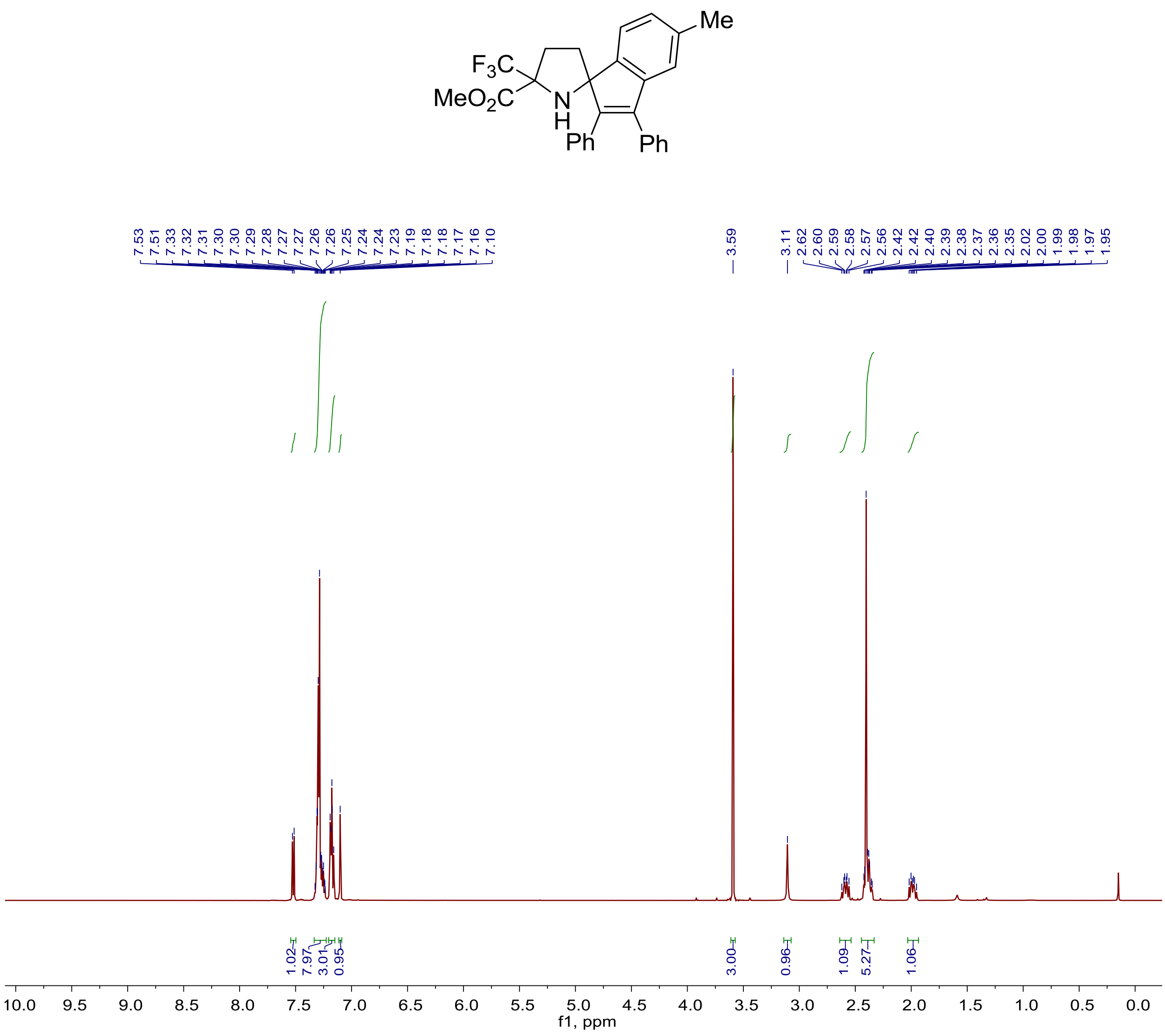

^{1}H spectrum of **4b** in $CDCl_3$

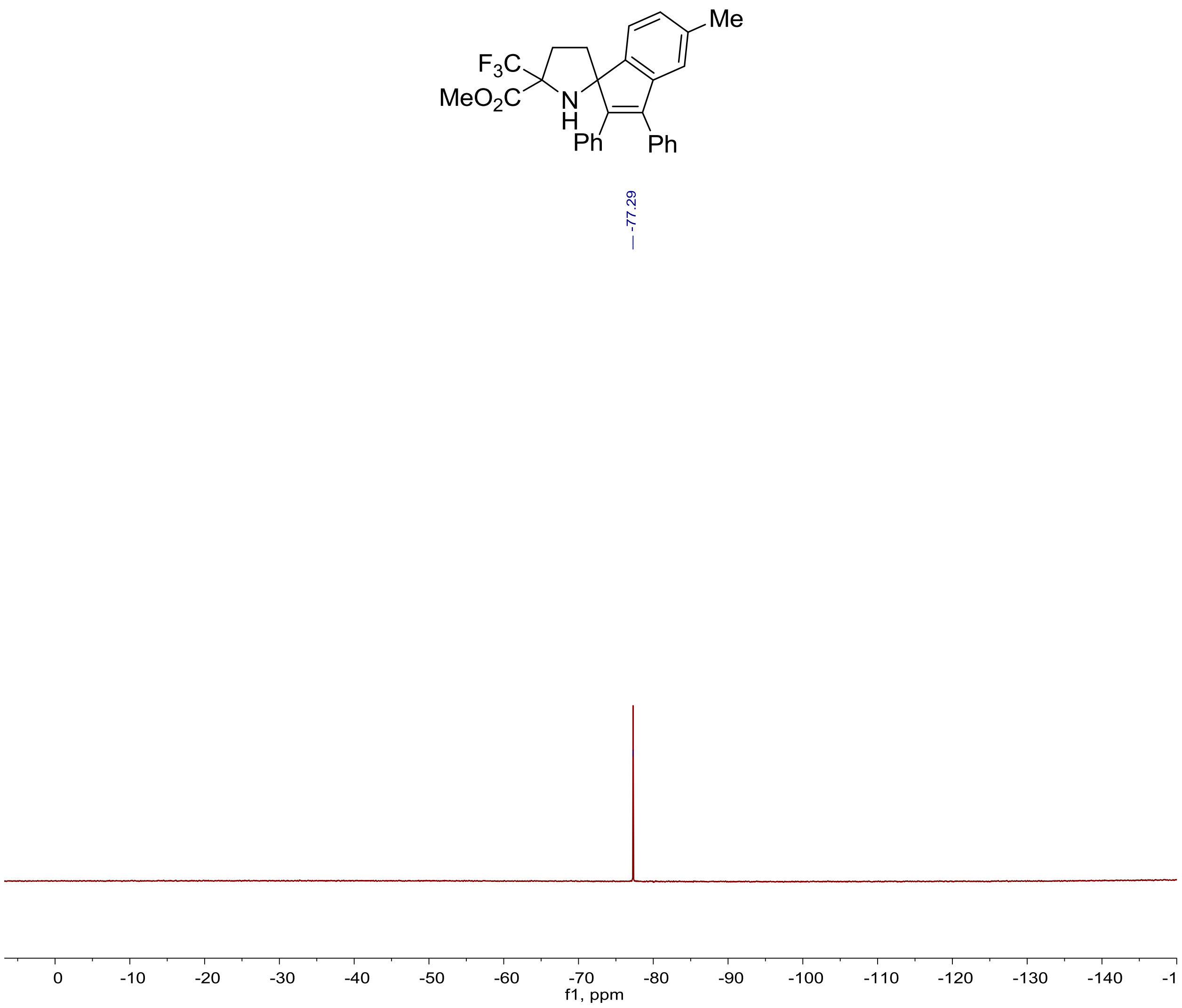

^{19}F spectrum of **4b** in $CDCl_3$

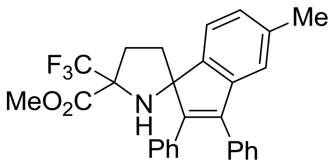

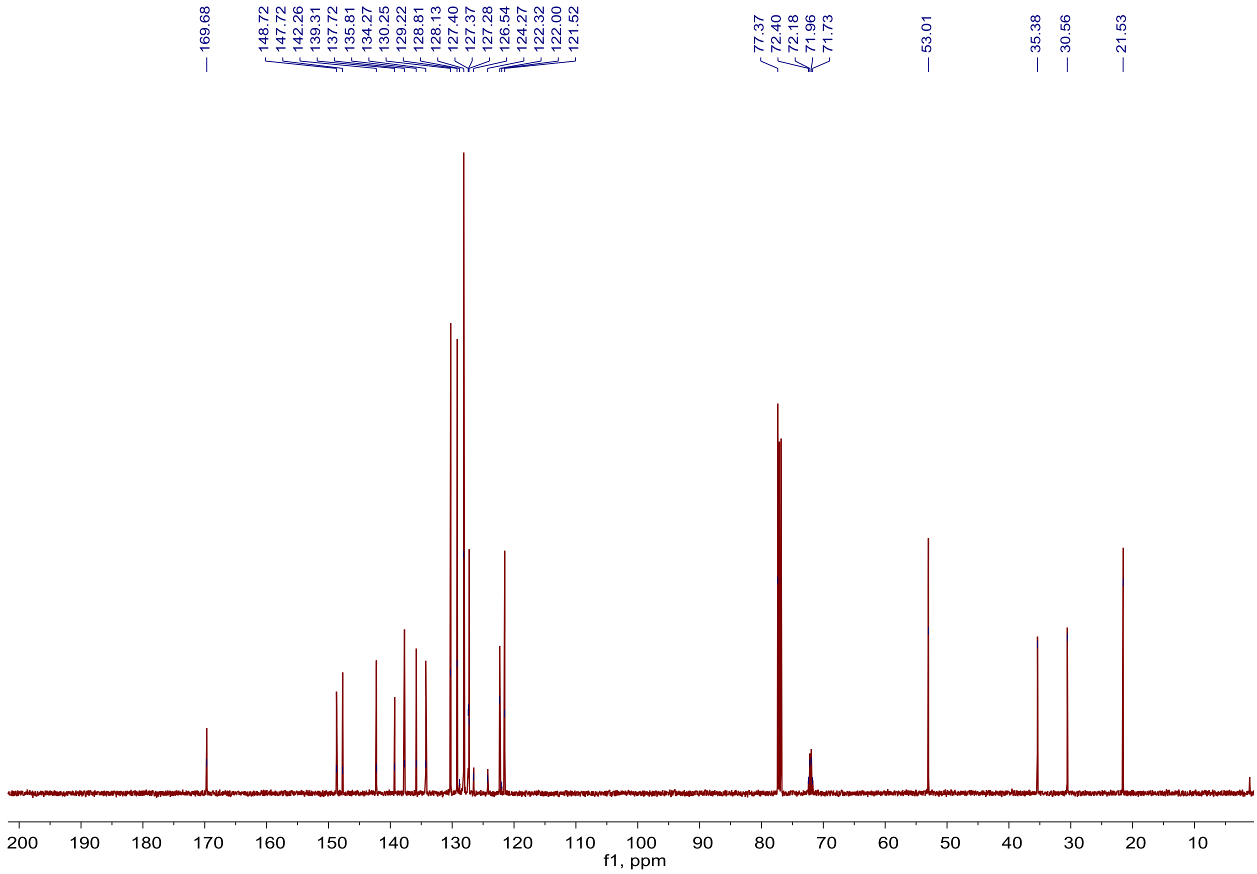

^{13}C spectrum of **4b** in $CDCl_3$

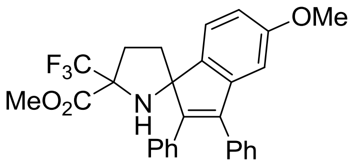

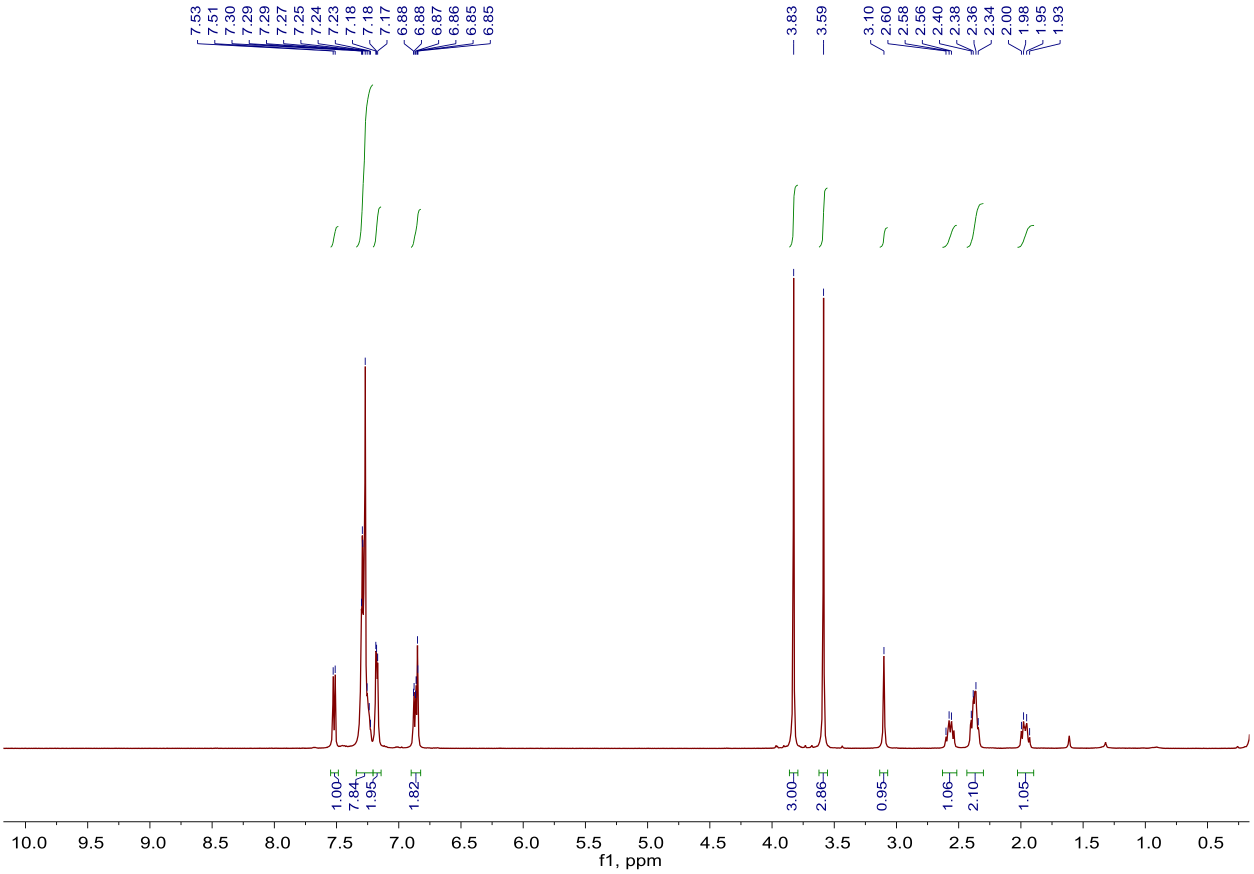

^{1}H spectrum of **4c** in $CDCl_3$

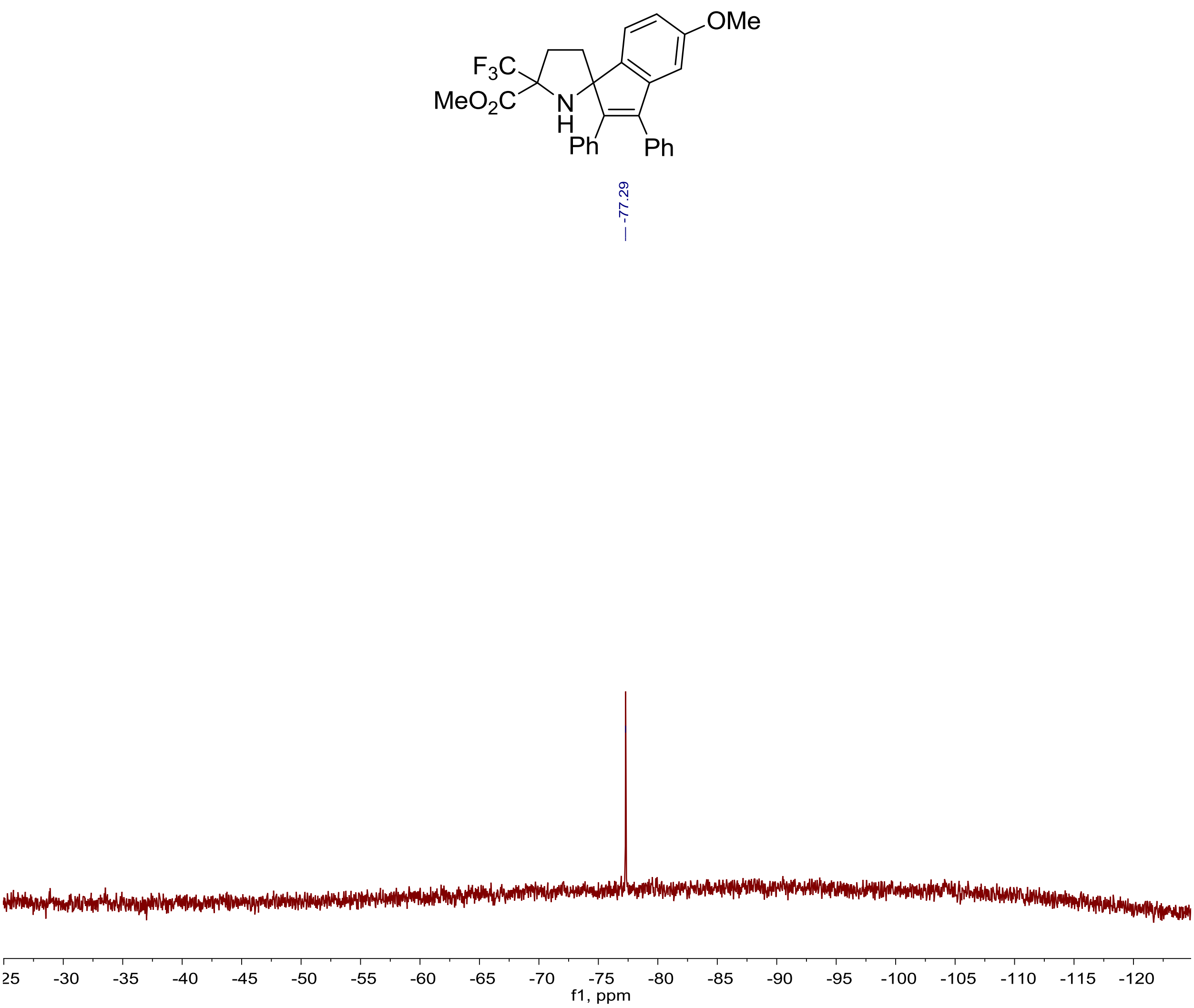

^{19}F spectrum of **4c** in $CDCl_3$

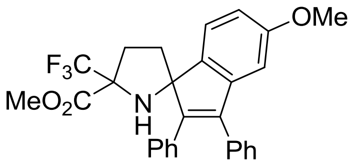

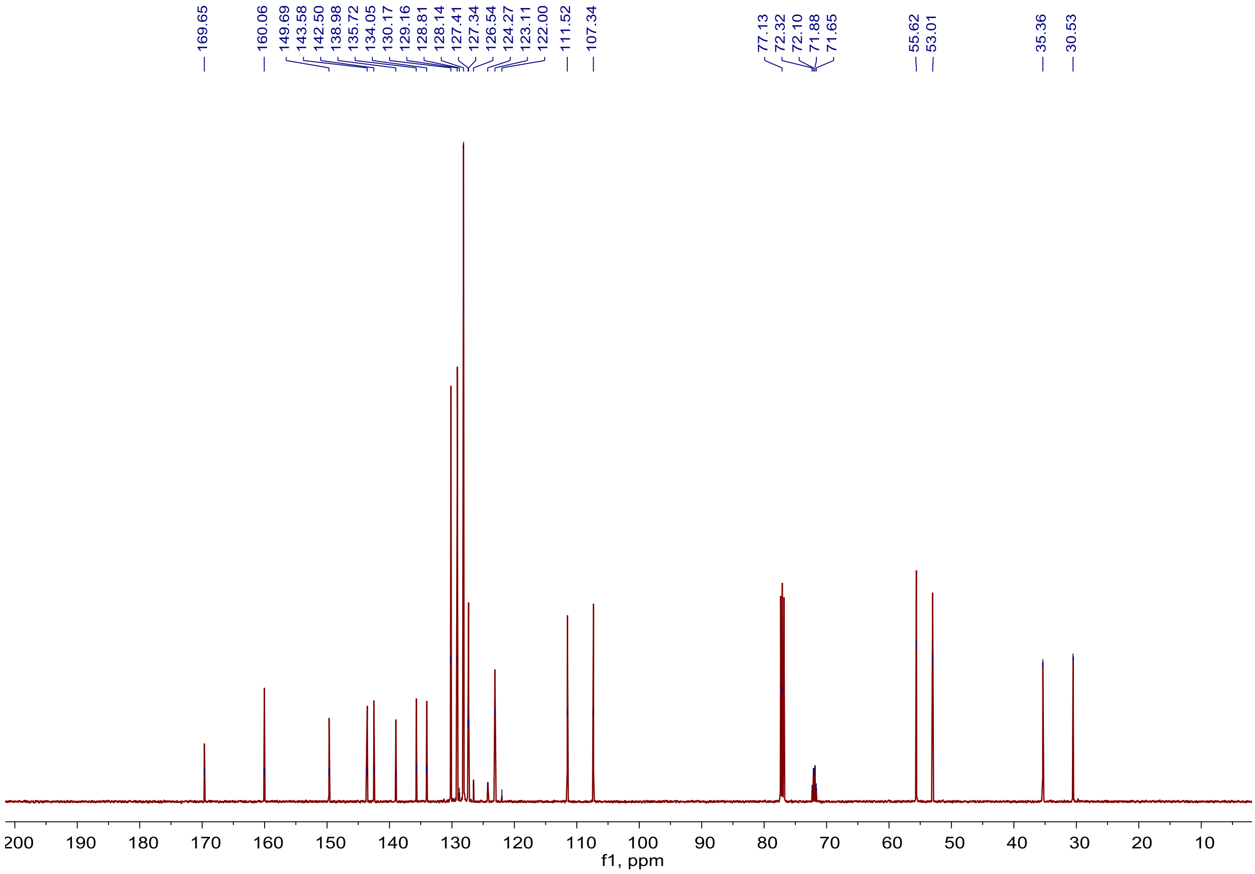

^{13}C spectrum of **4c** in $CDCl_3$

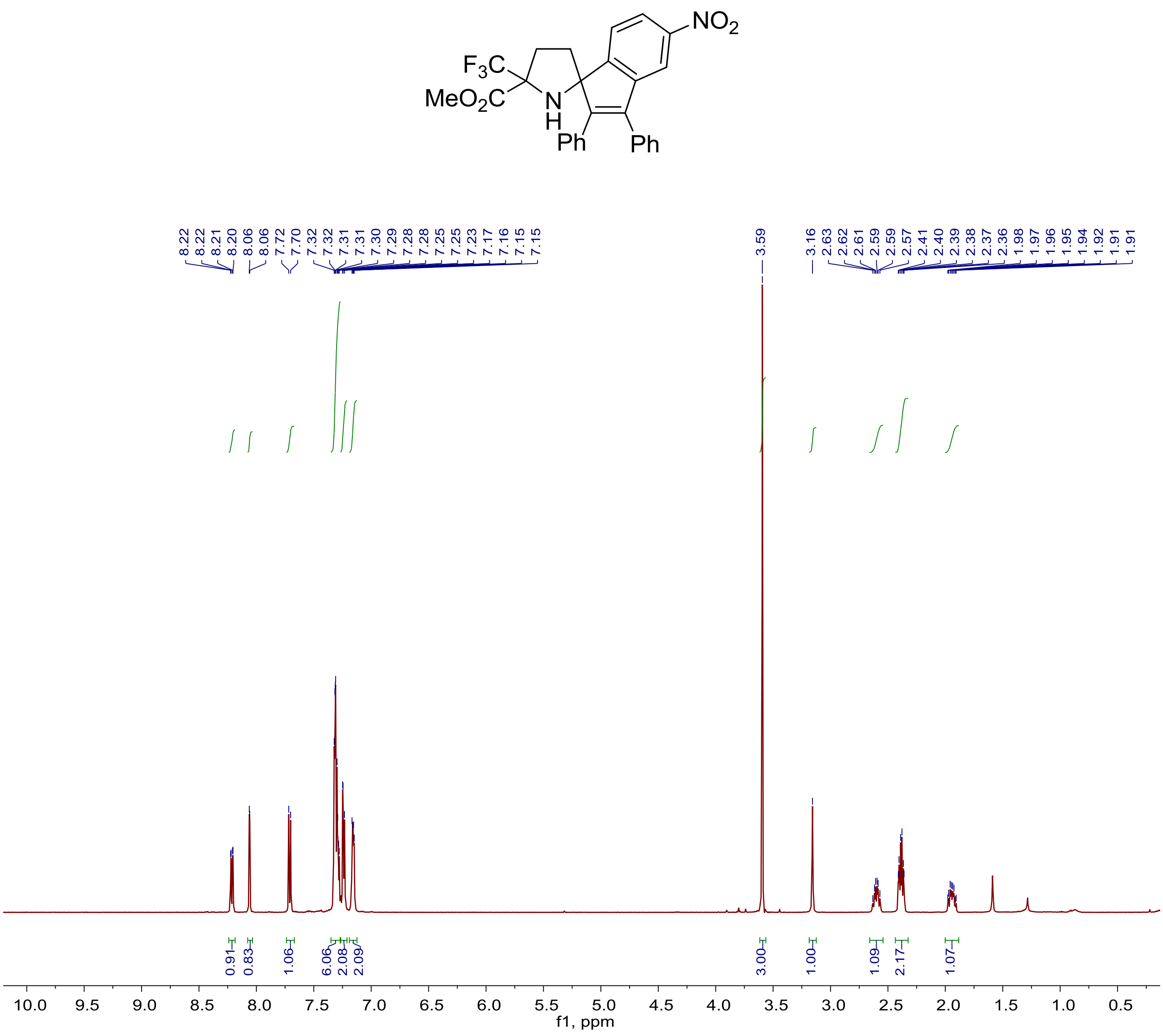

^{1}H spectrum of **4d** in $CDCl_3$

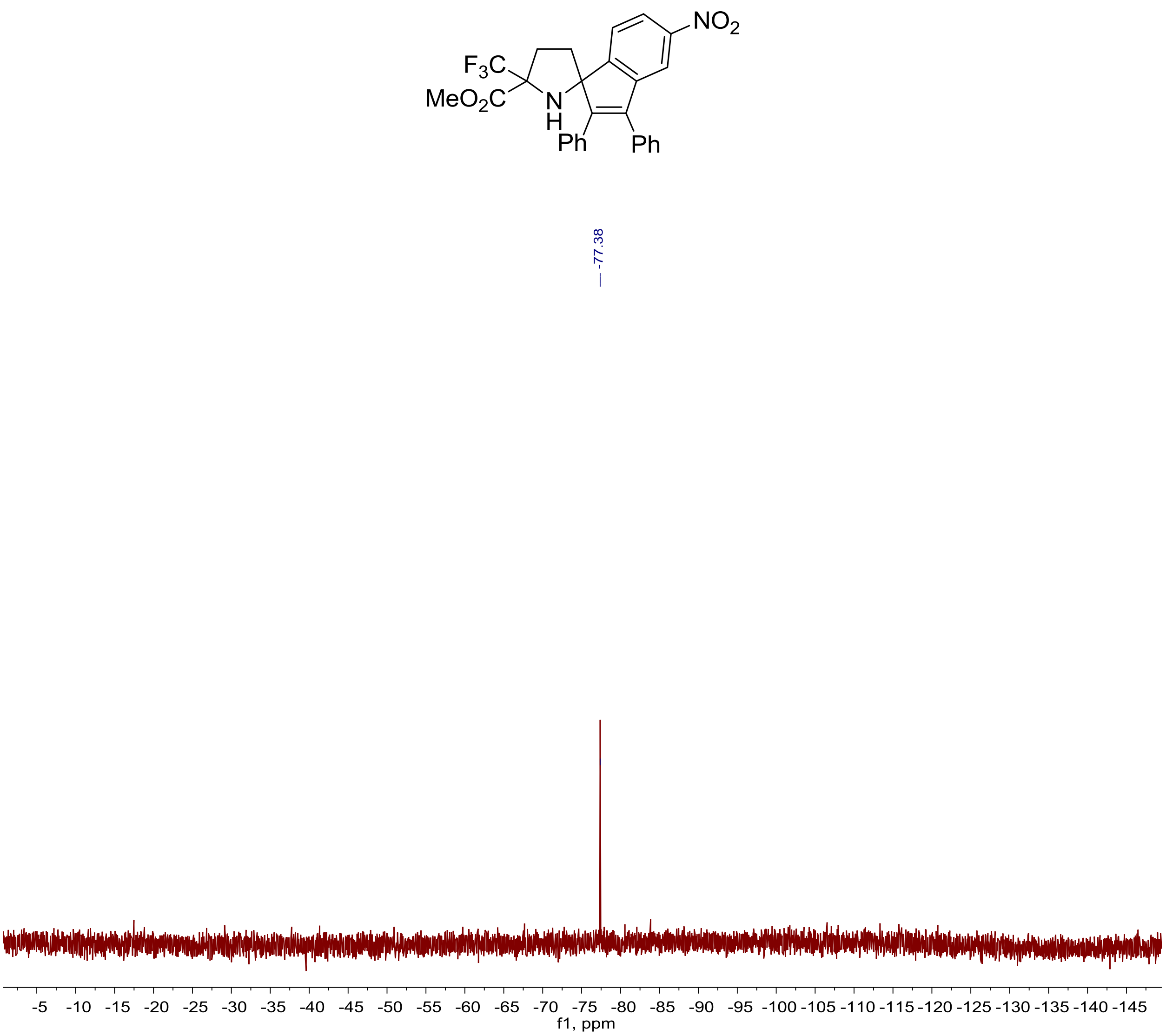

^{19}F spectrum of **4d** in $CDCl_3$

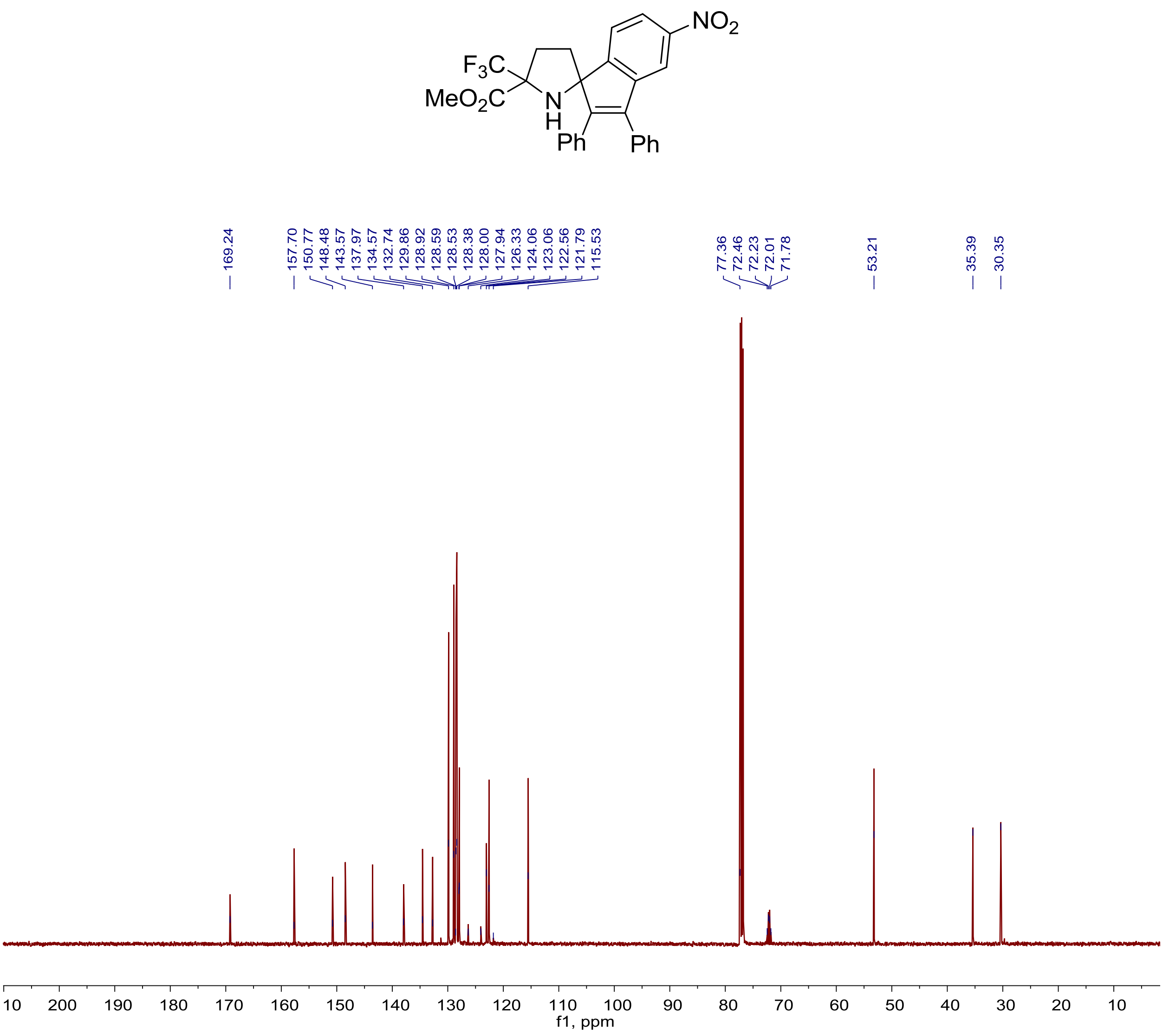

^{13}C spectrum of **4d** in $CDCl_3$

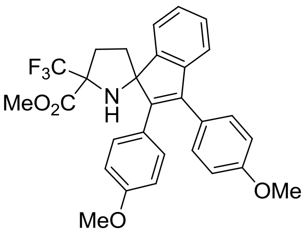

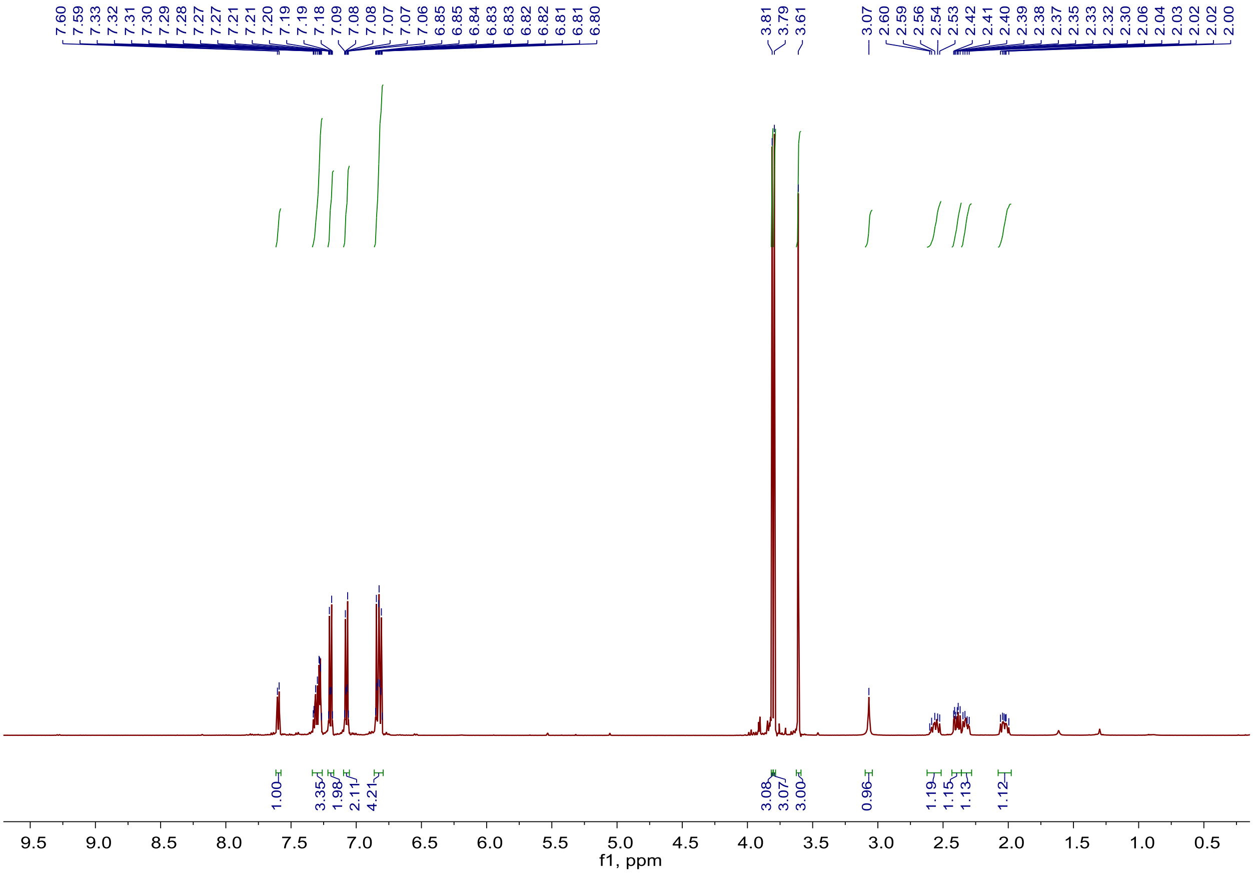

^{1}H spectrum of **4e** in $CDCl_3$

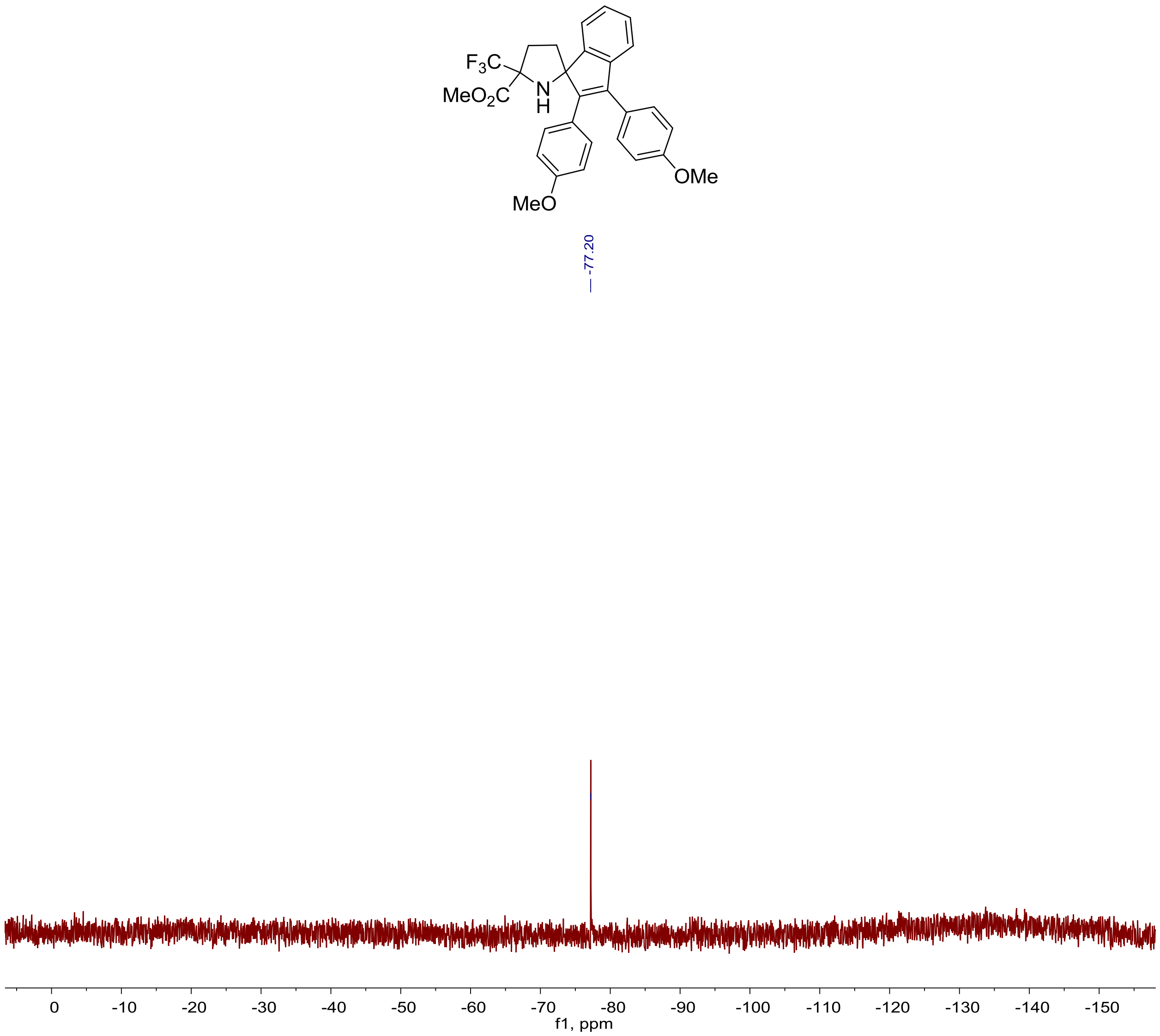

^{19}F spectrum of **4e** in $CDCl_3$

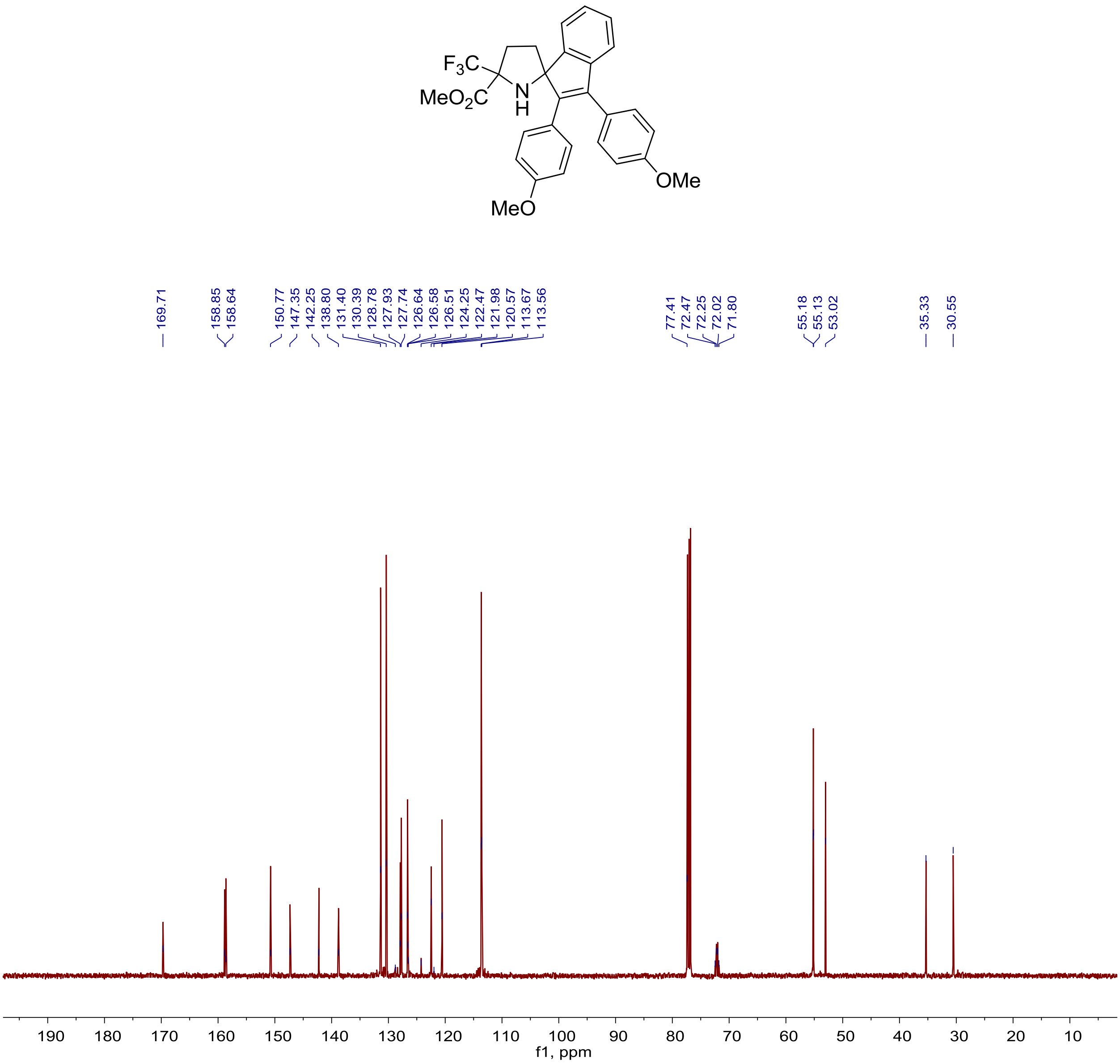

^{13}C spectrum of **4e** in $CDCl_3$

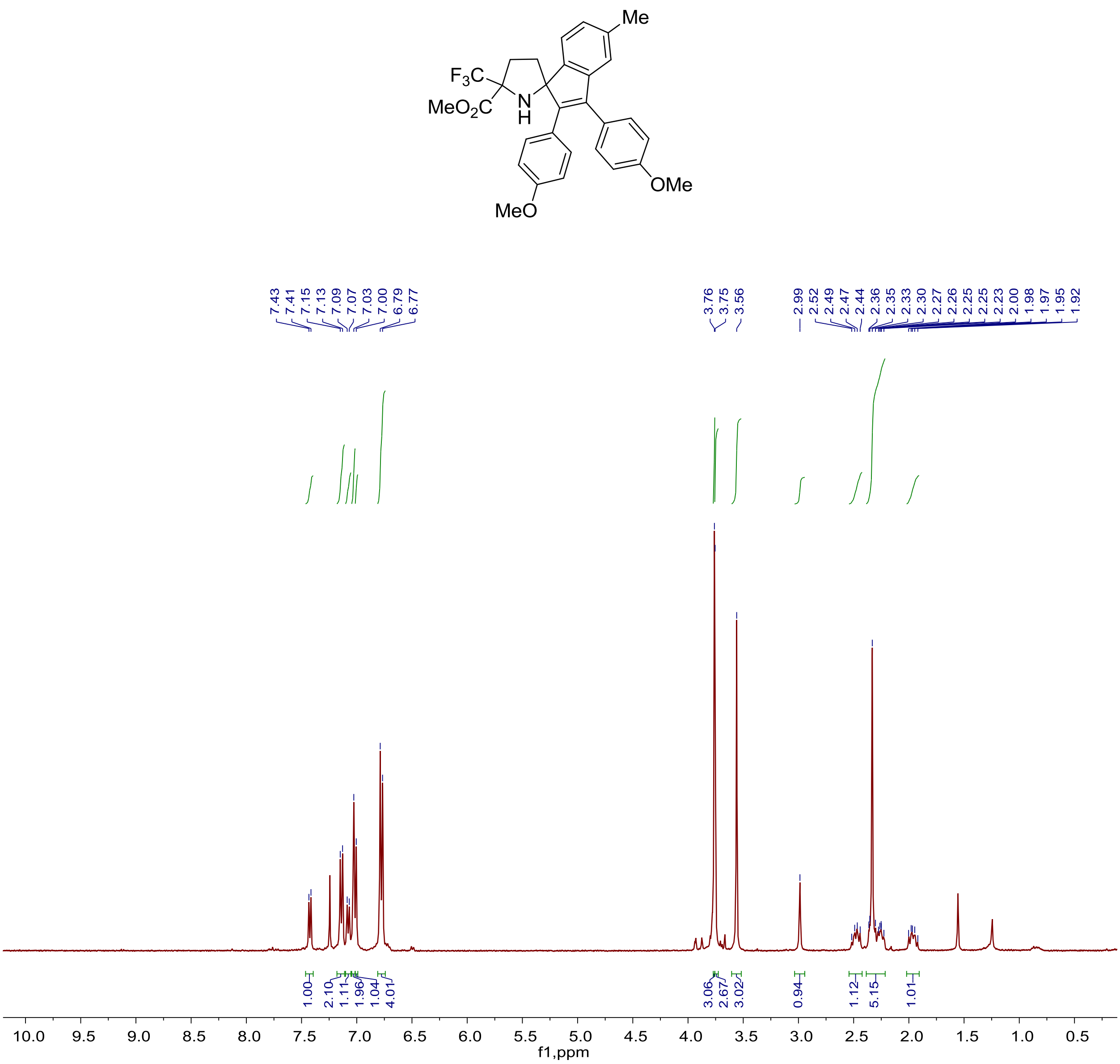

^{1}H spectrum of **4f** in $CDCl_3$

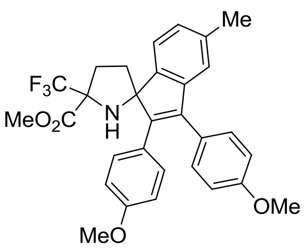

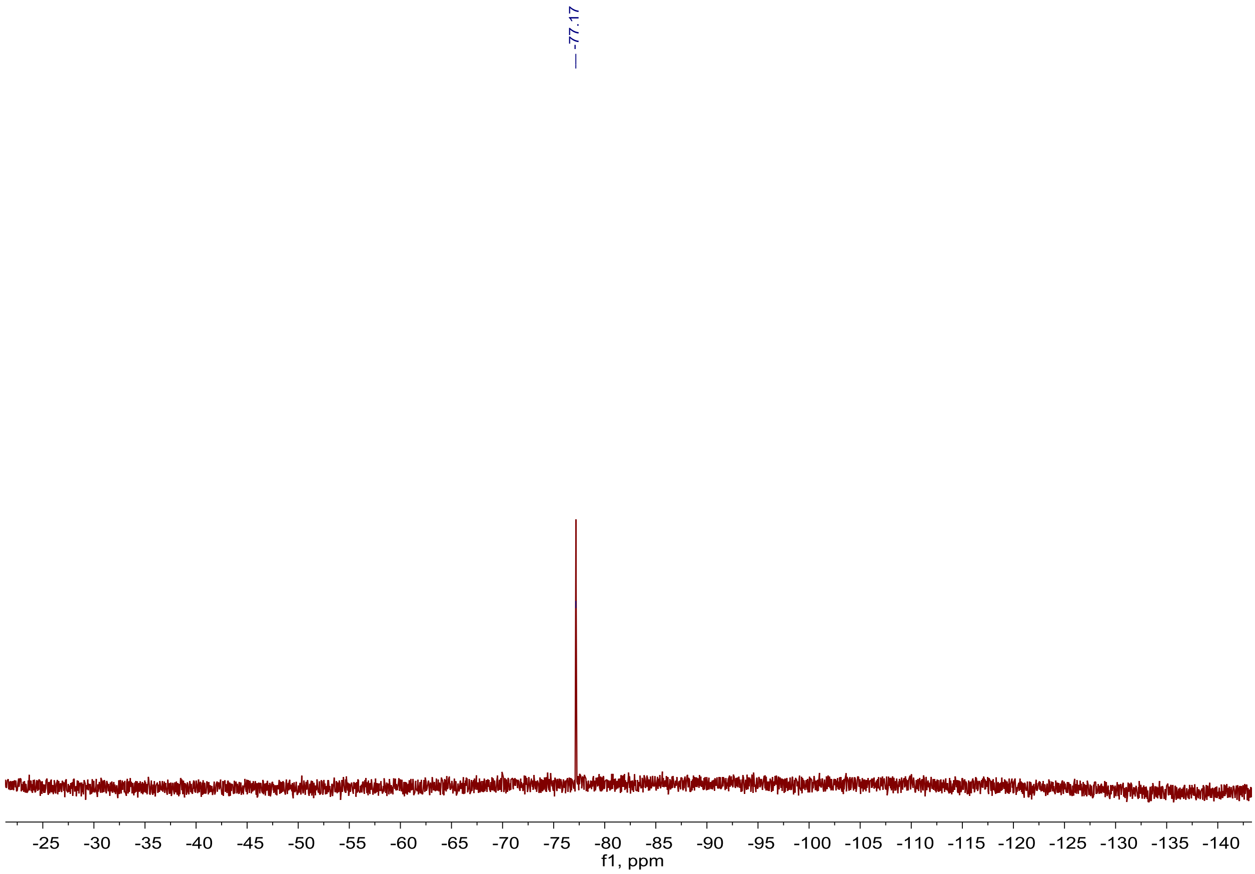

^{19}F spectrum of **4f** in $CDCl_3$

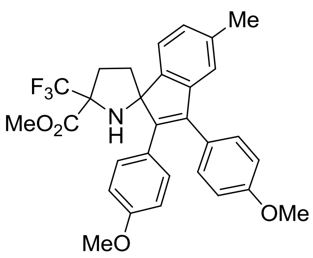

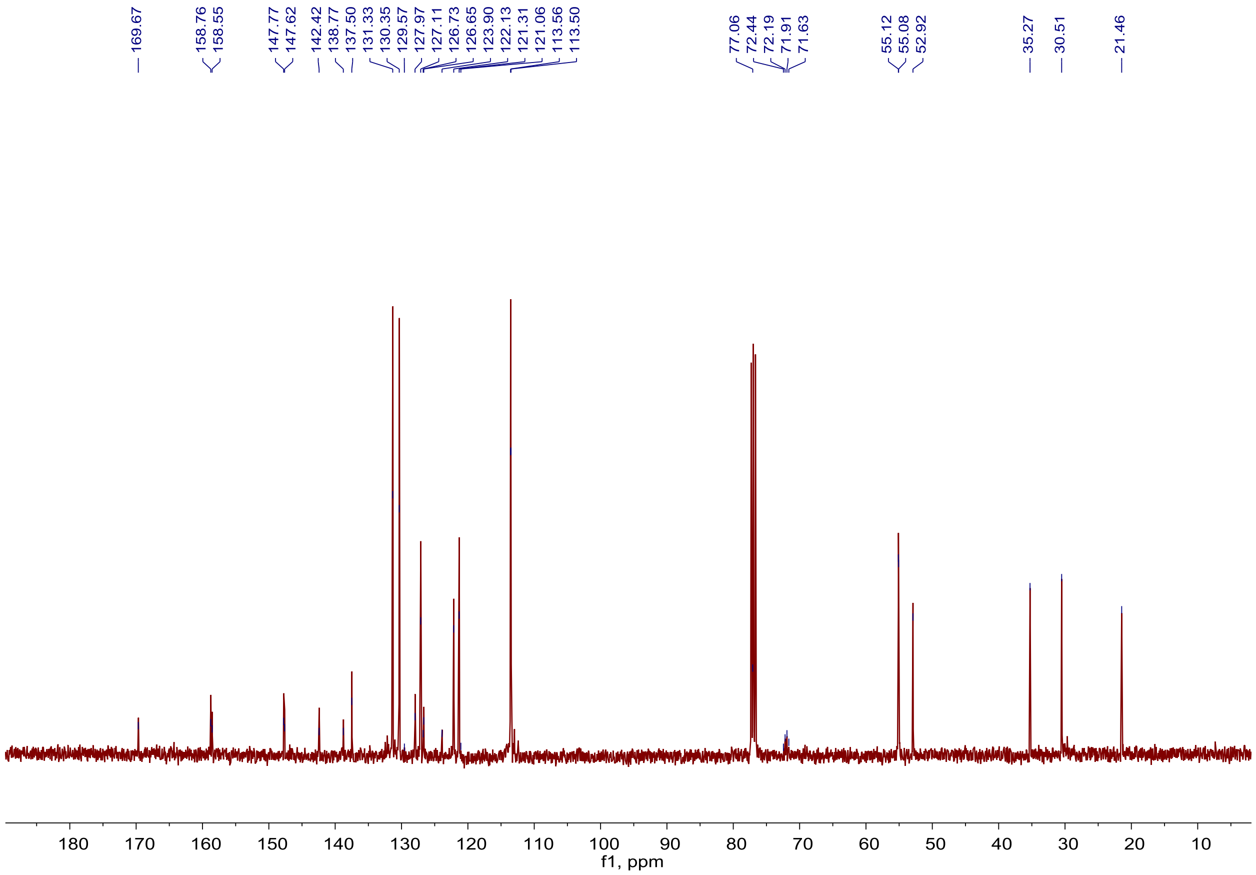

^{13}C spectrum of **4f** in $CDCl_3$

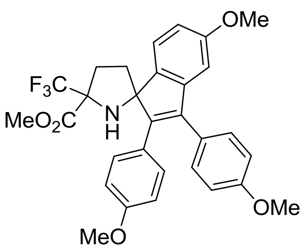

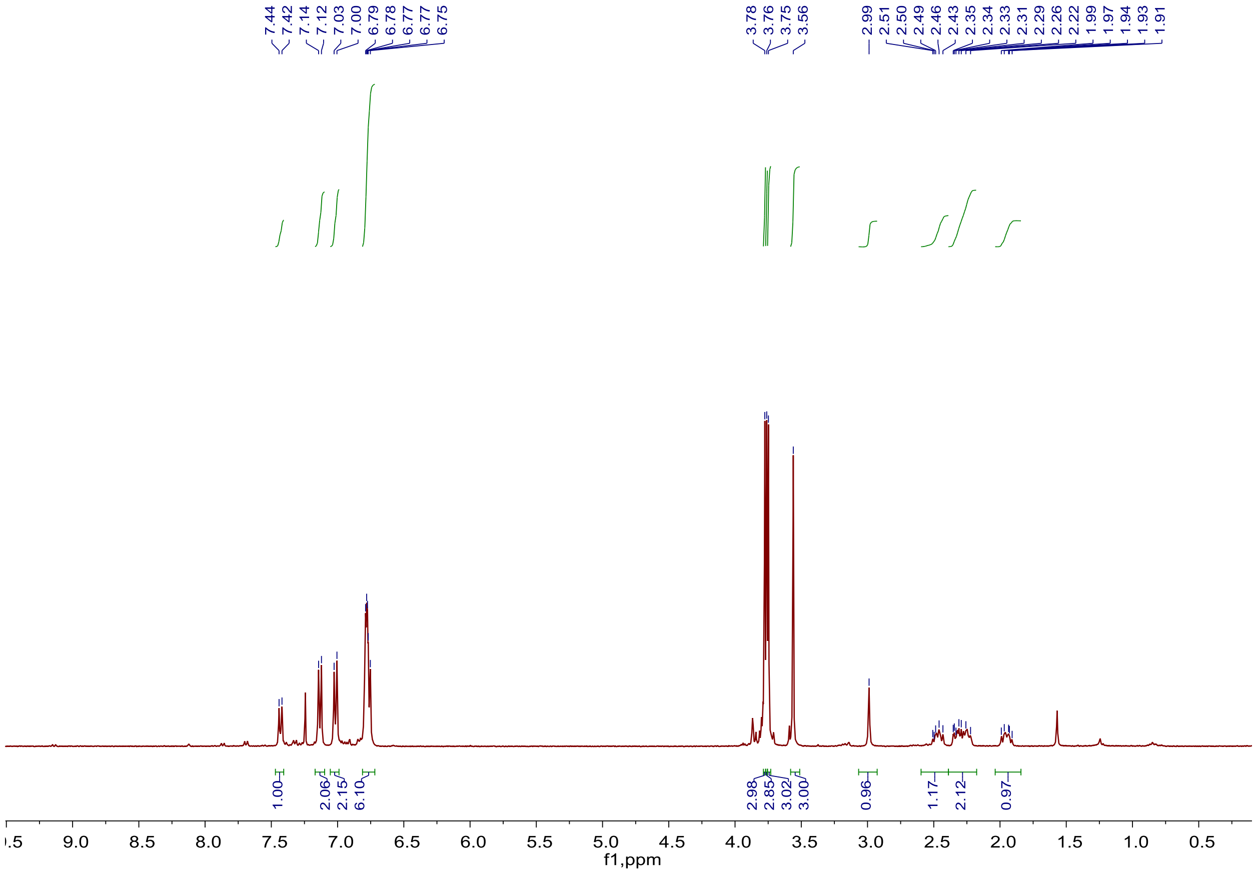

^{1}H spectrum of **4g** in $CDCl_3$

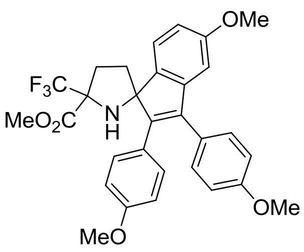

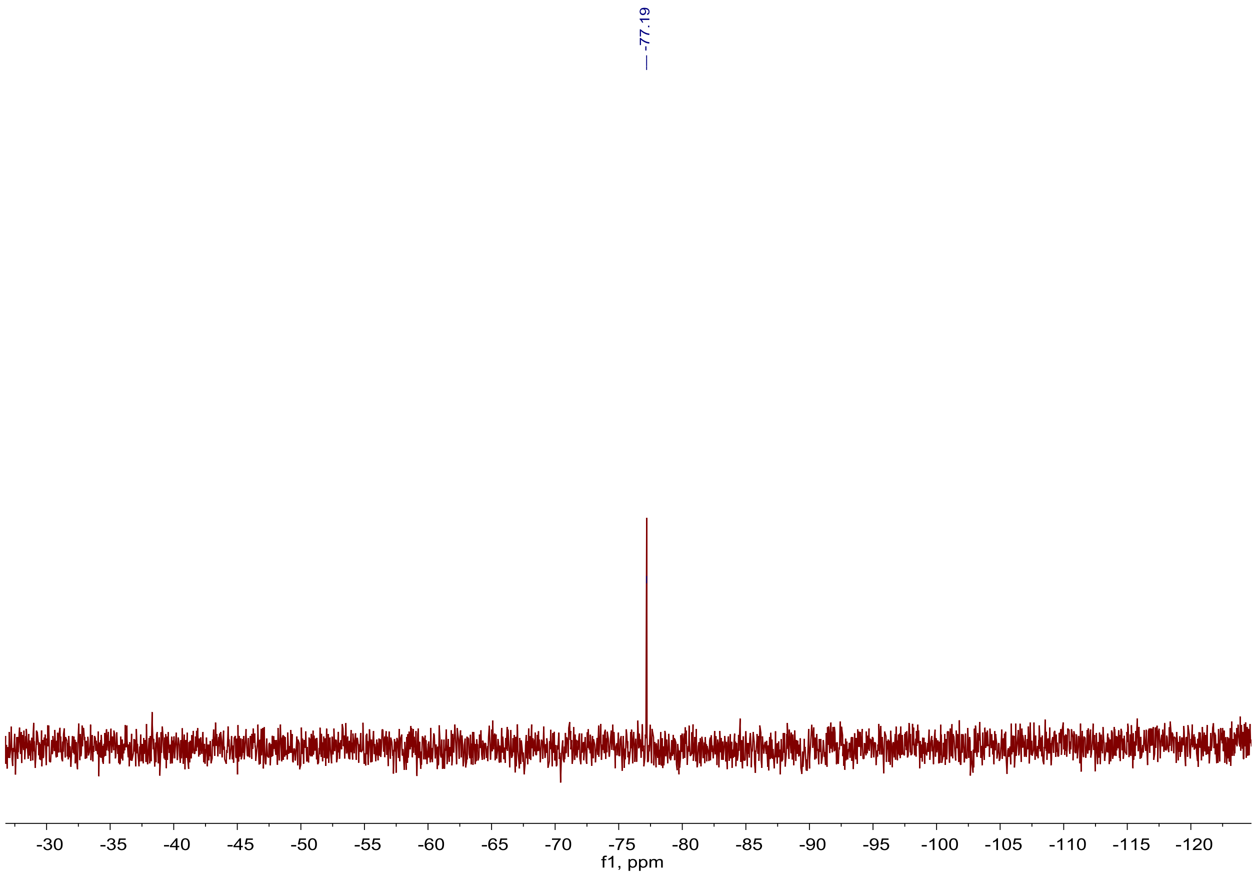

^{19}F spectrum of **4g** in $CDCl_3$

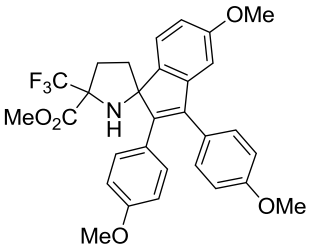

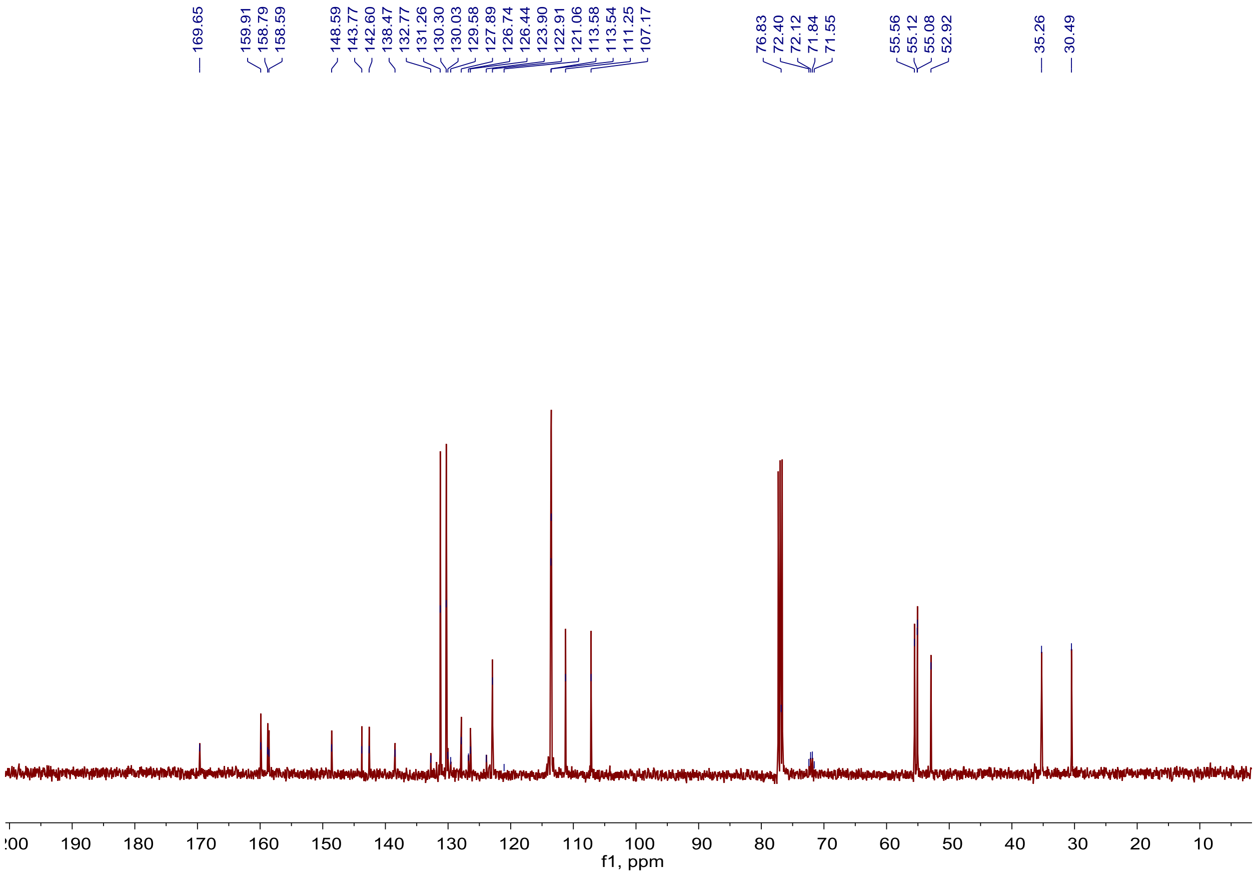

^{13}C spectrum of **4g** in $CDCl_3$

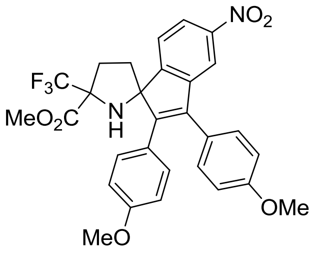

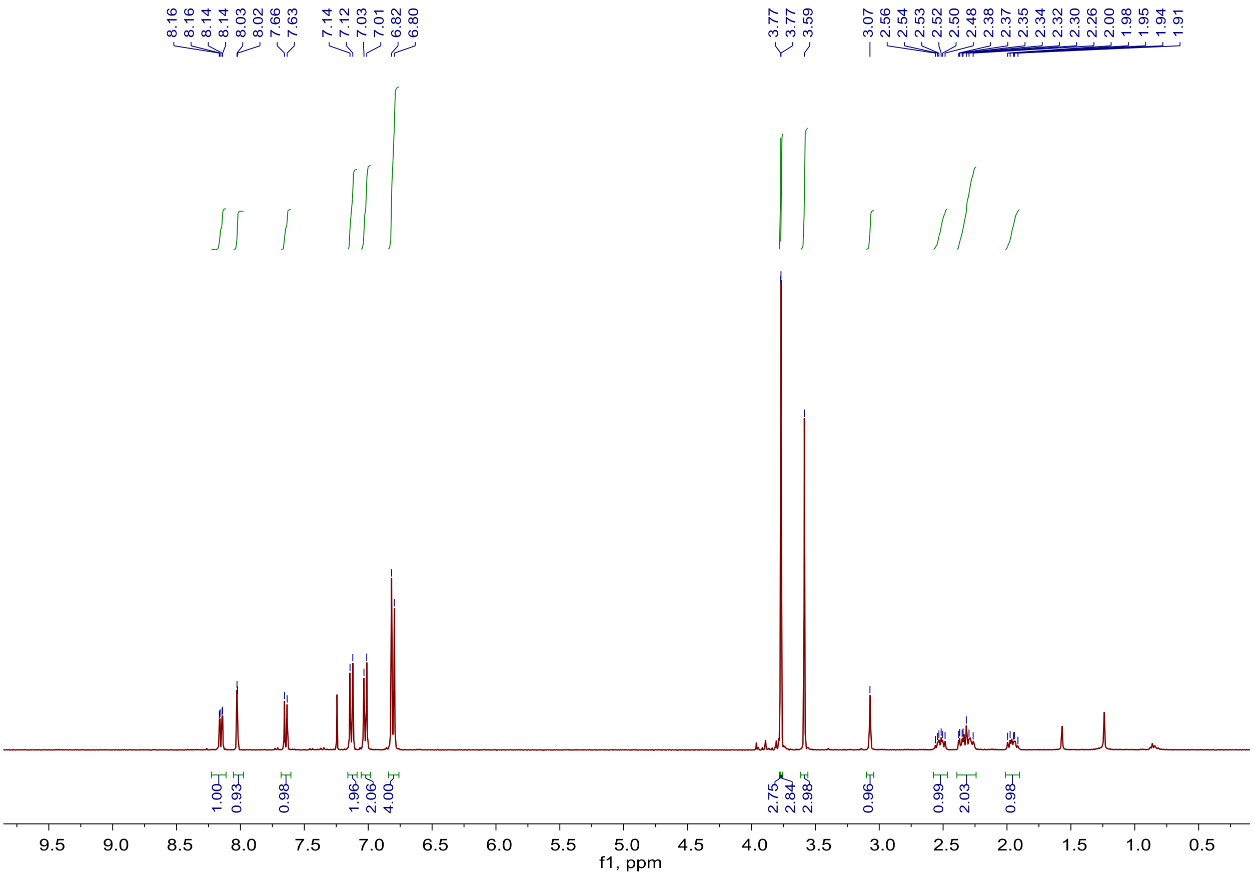

^{1}H spectrum of **4h** in $CDCl_3$

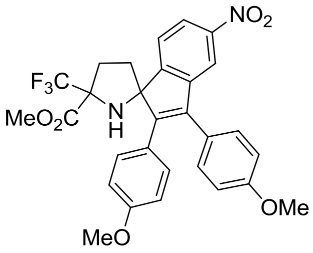

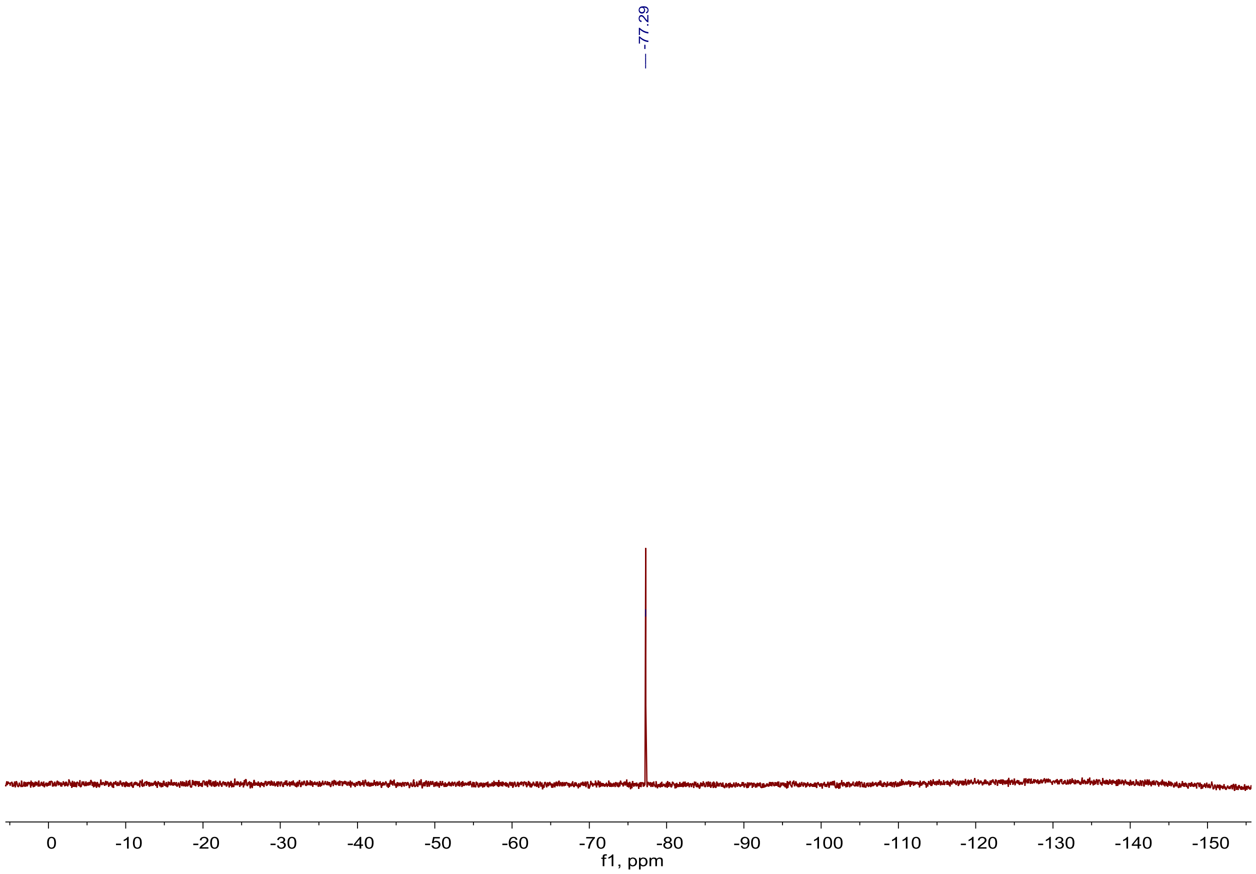

^{19}F spectrum of **4h** in $CDCl_3$

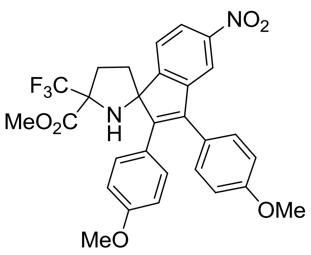

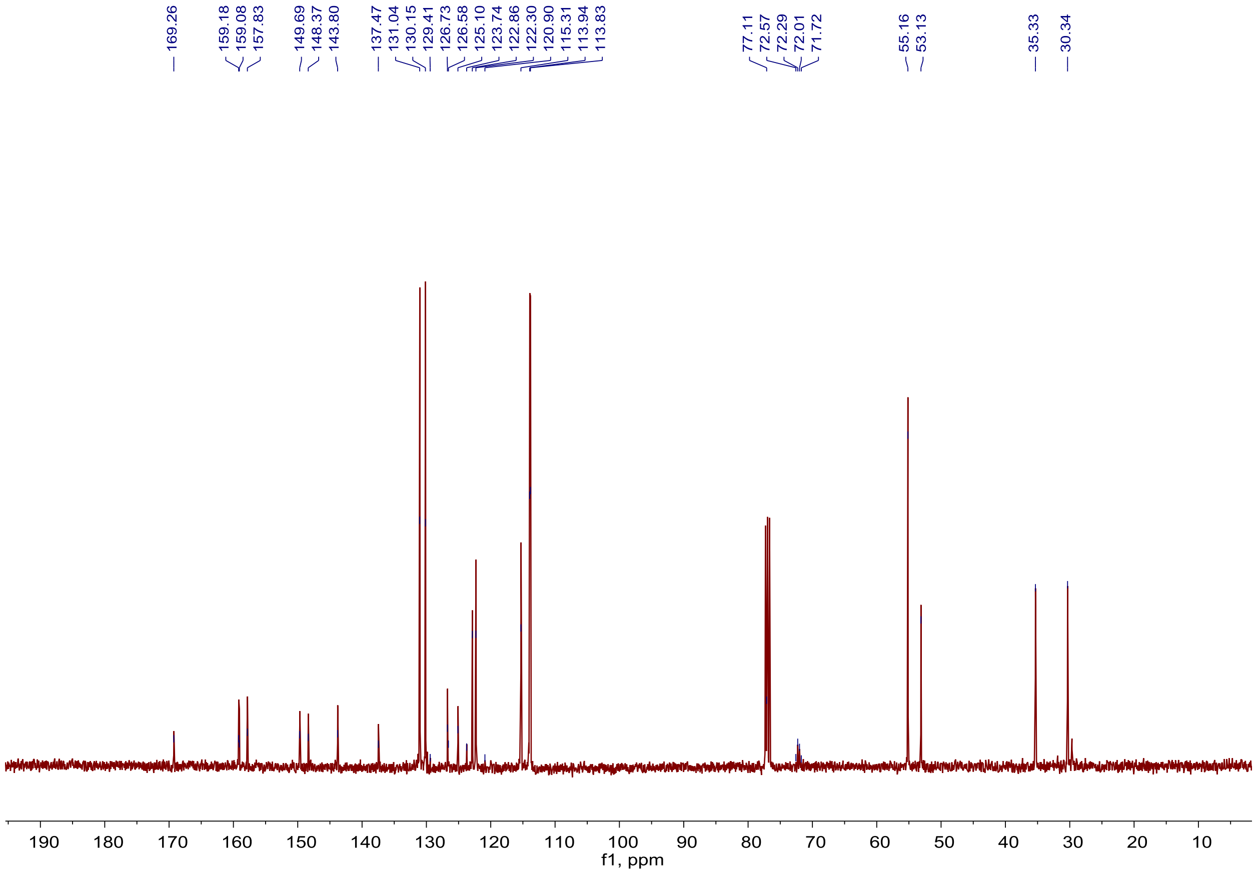

^{13}C spectrum of **4h** in $CDCl_3$

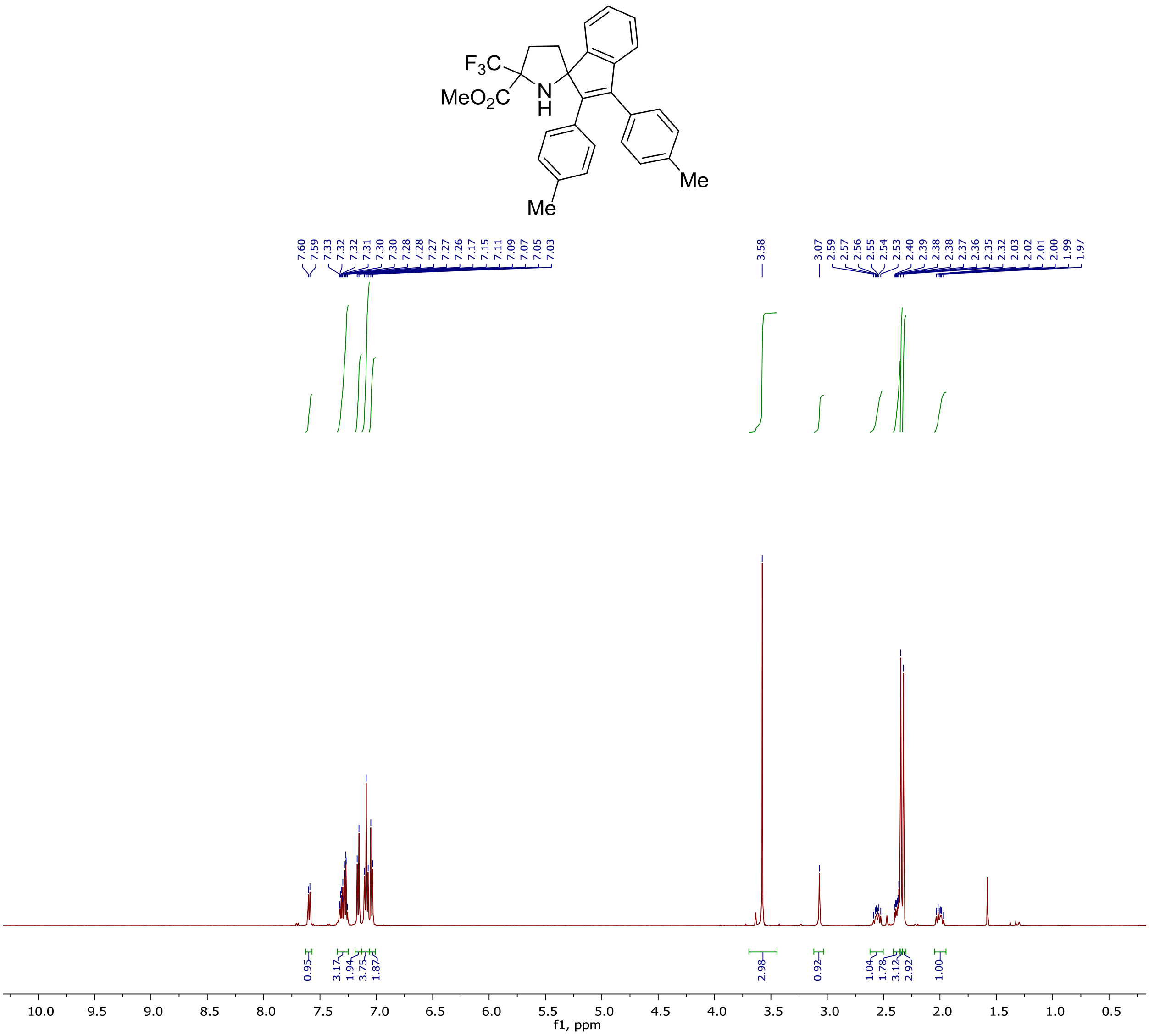

^{1}H spectrum of **4i** in $CDCl_3$

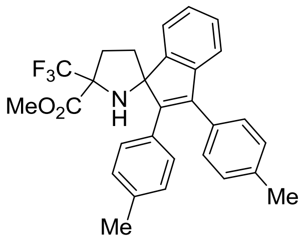

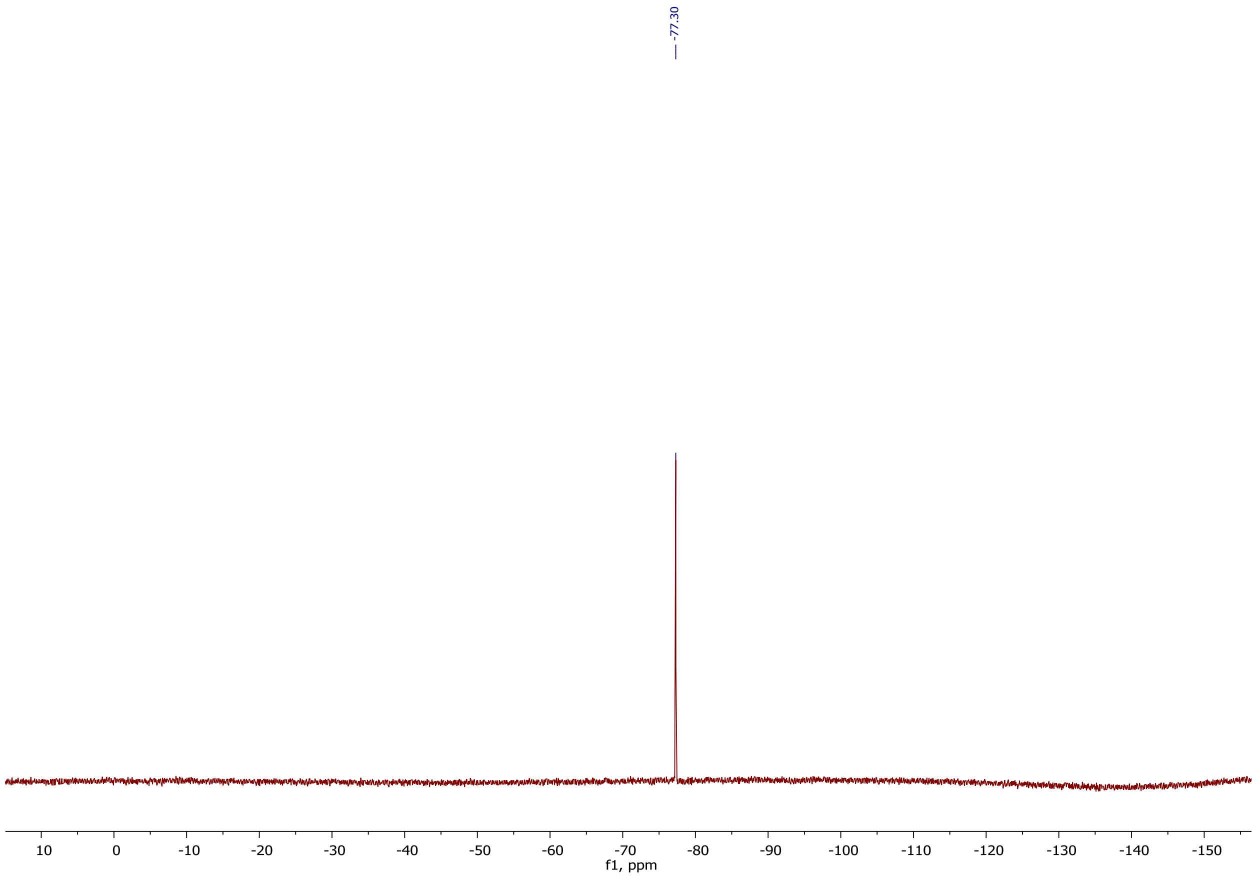

^{19}F spectrum of **4i** in $CDCl_3$

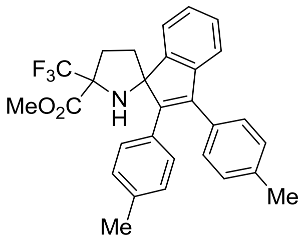

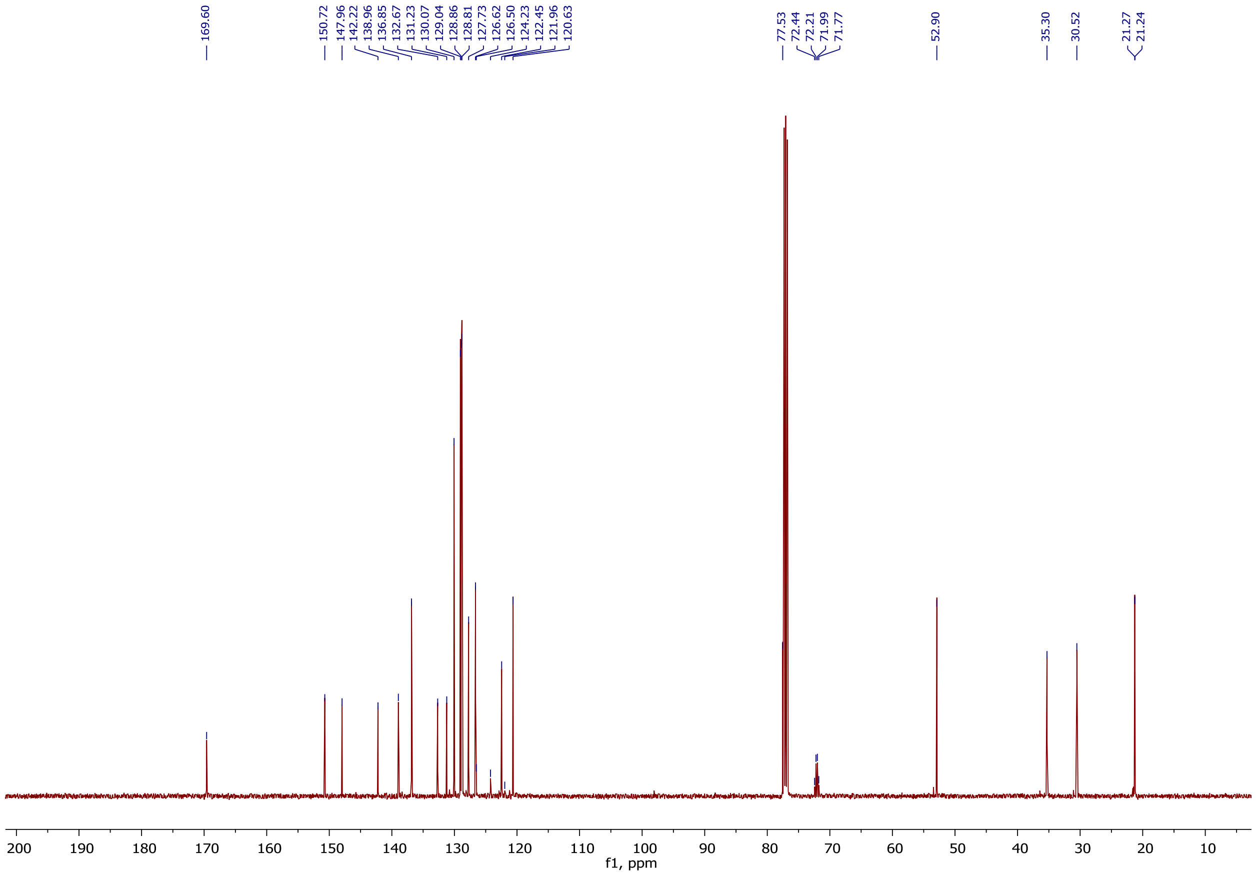

^{13}C spectrum of **4i** in $CDCl_3$

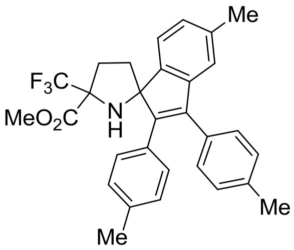

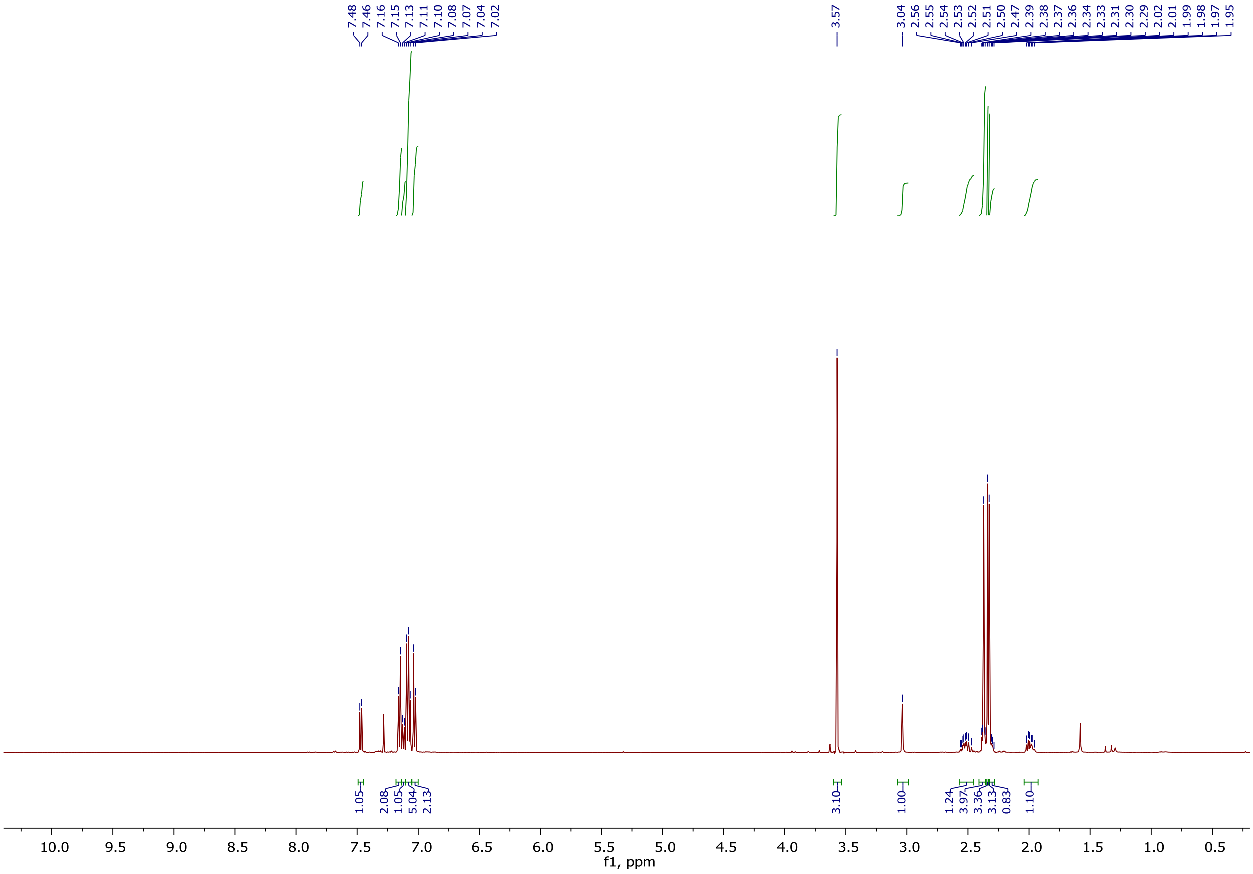

^{1}H spectrum of **4j** in $CDCl_3$

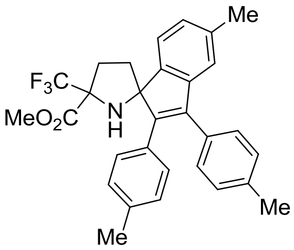

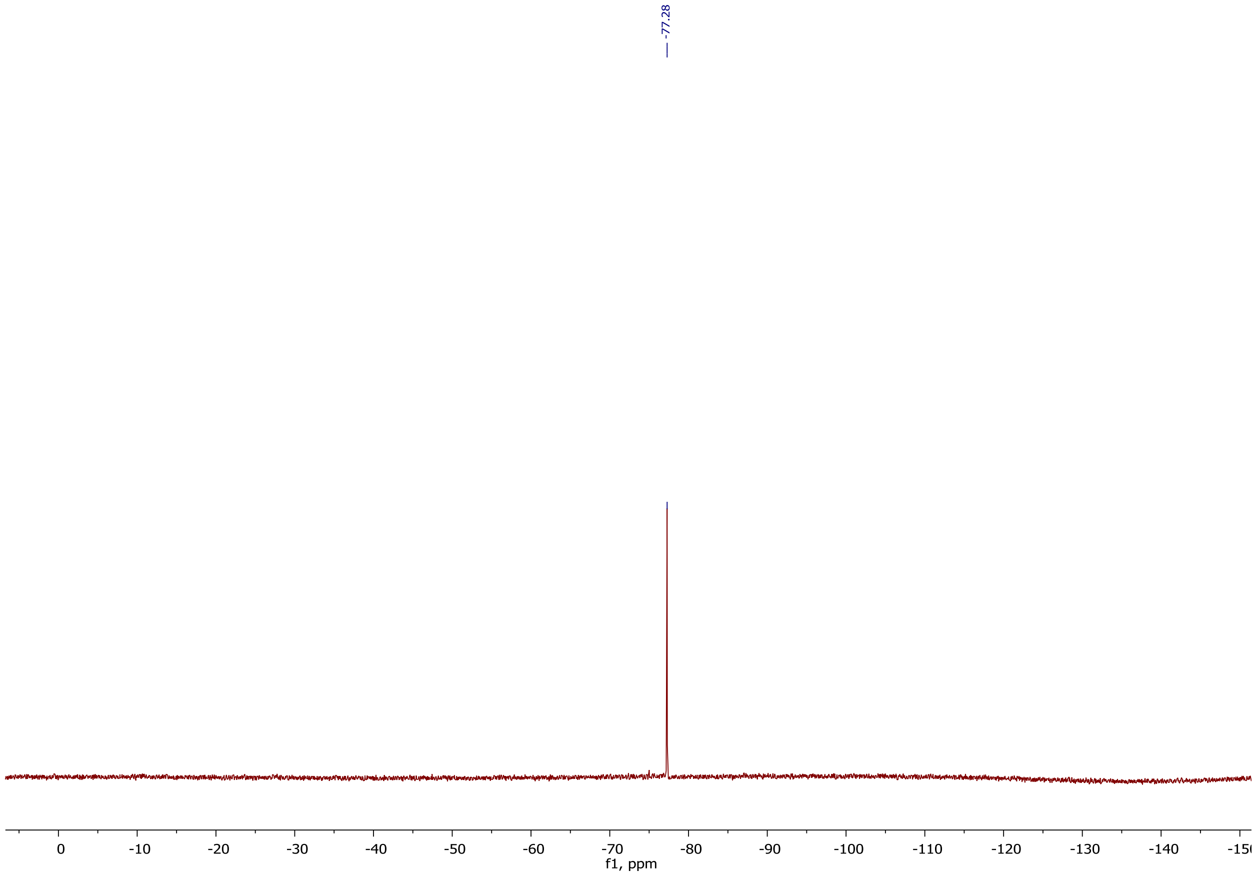

^{19}F spectrum of **4j** in $CDCl_3$

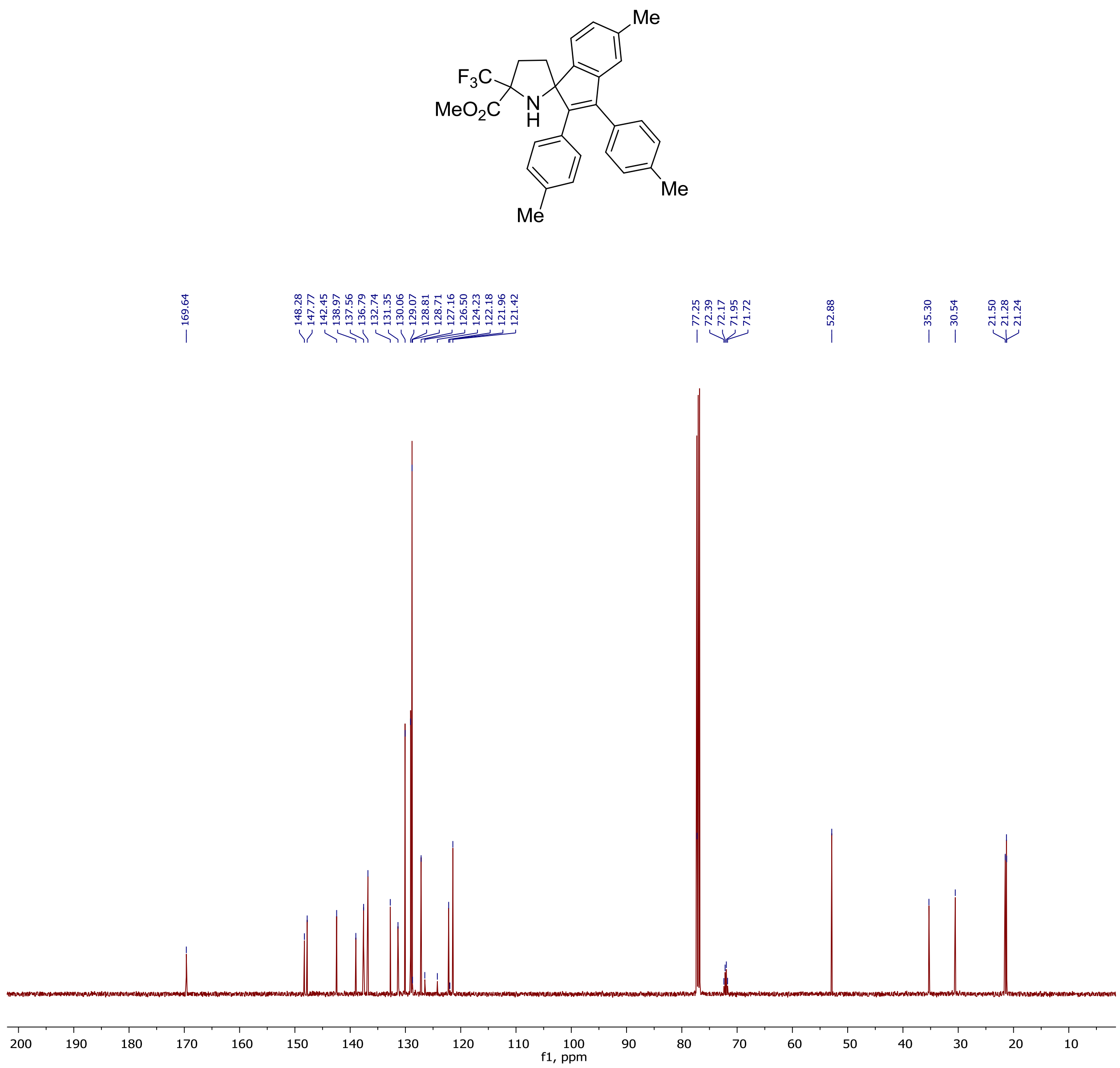

^{13}C spectrum of **4j** in $CDCl_3$

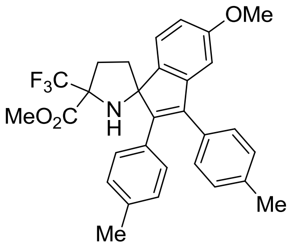

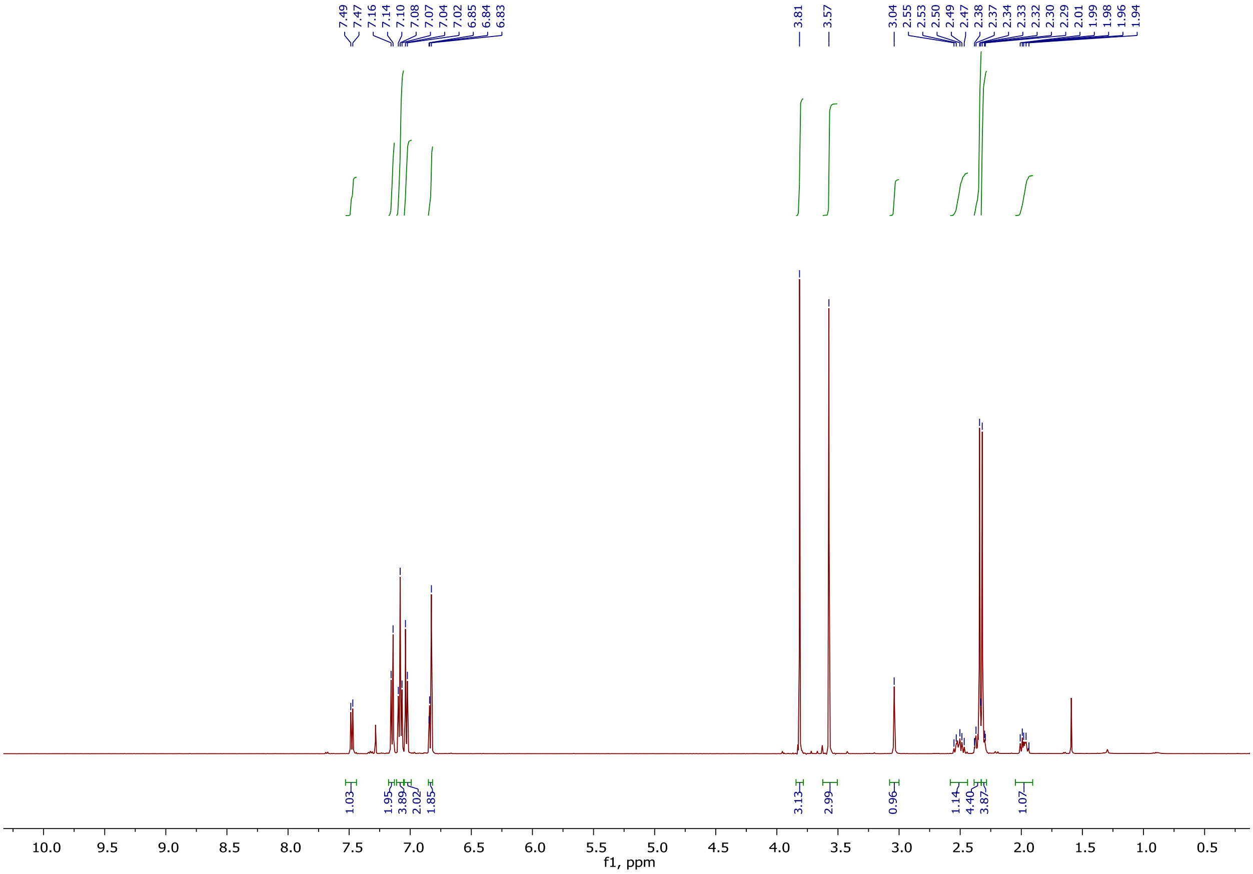

^{1}H spectrum of **4k** in $CDCl_3$

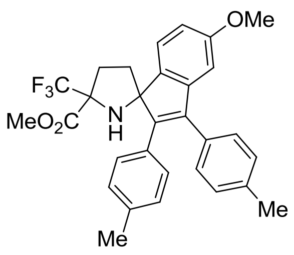

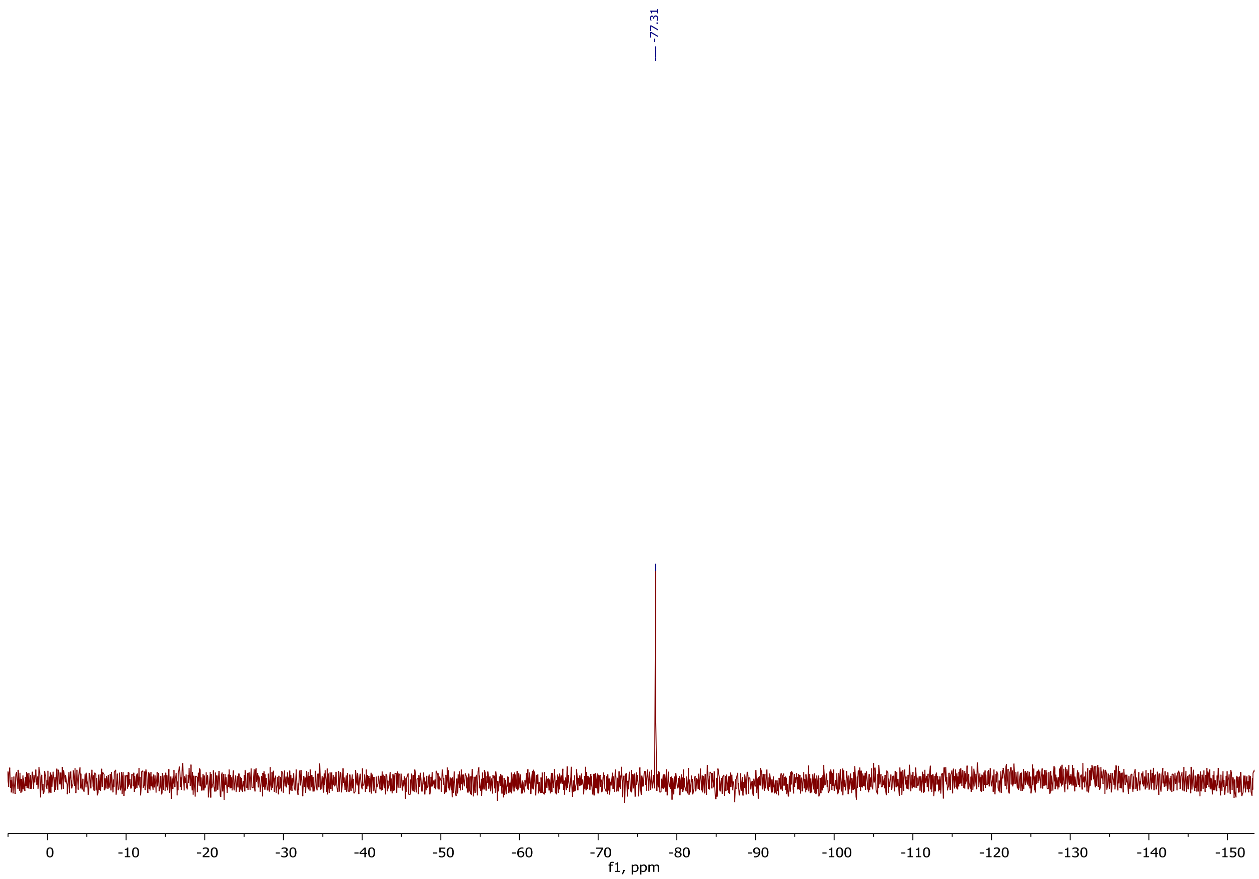

^{19}F spectrum of **4k** in $CDCl_3$

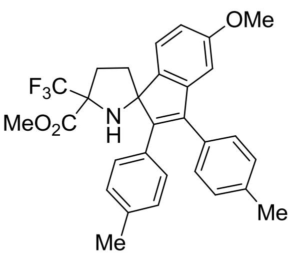

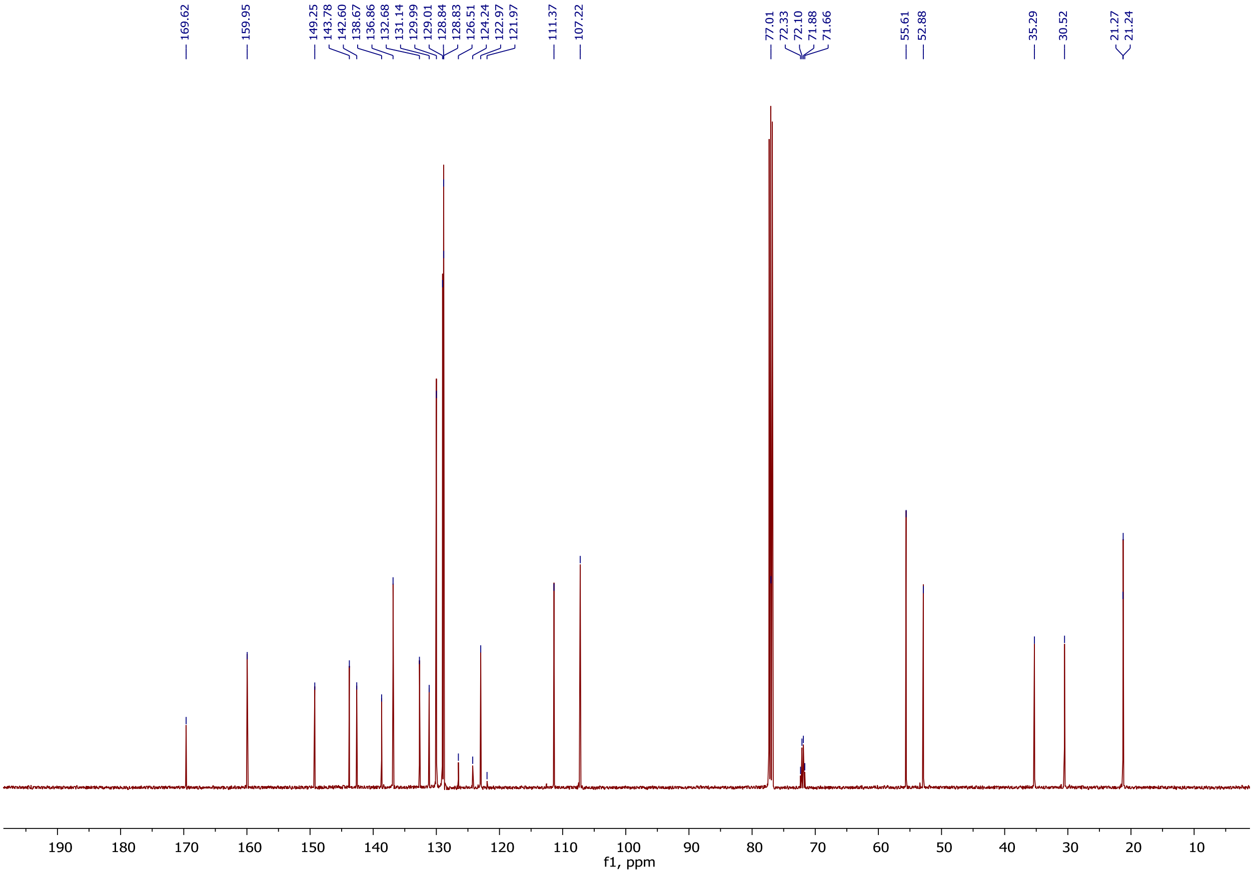

^{13}C spectrum of **4k** in $CDCl_3$

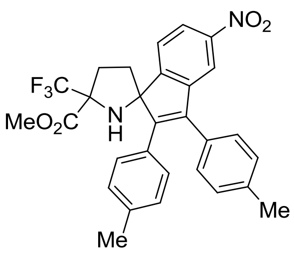

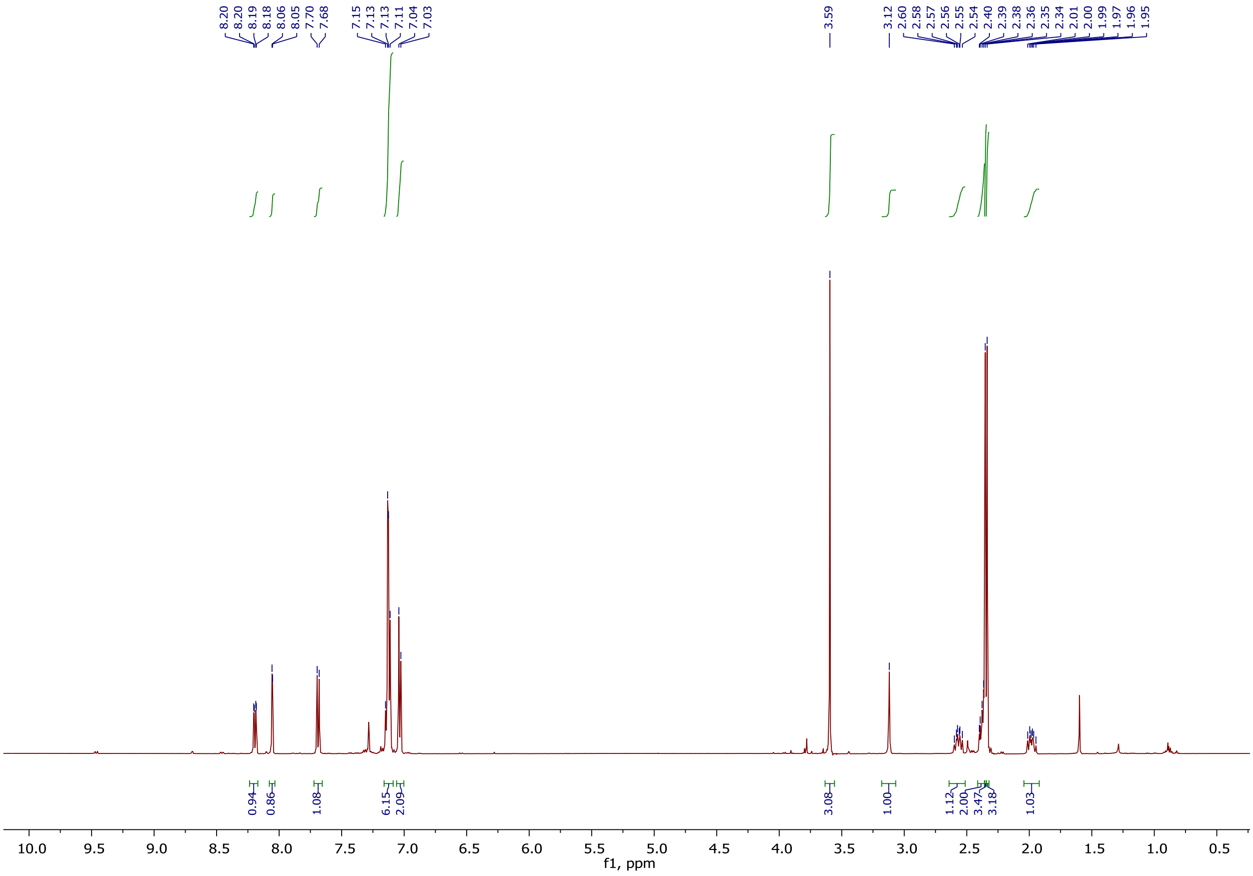

^{1}H spectrum of **4l** in $CDCl_3$

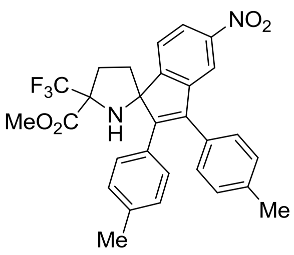

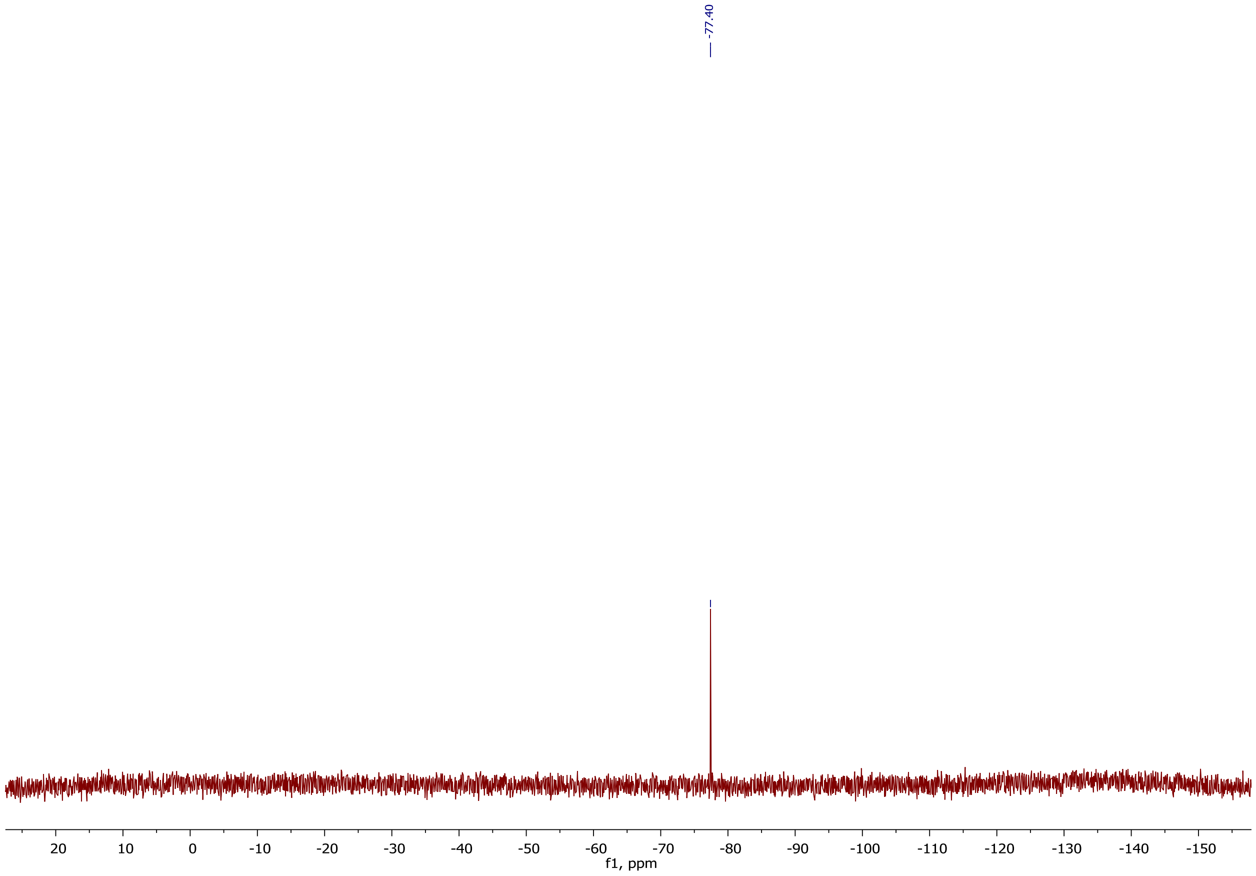

^{19}F spectrum of **4l** in $CDCl_3$

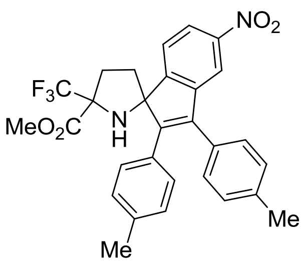

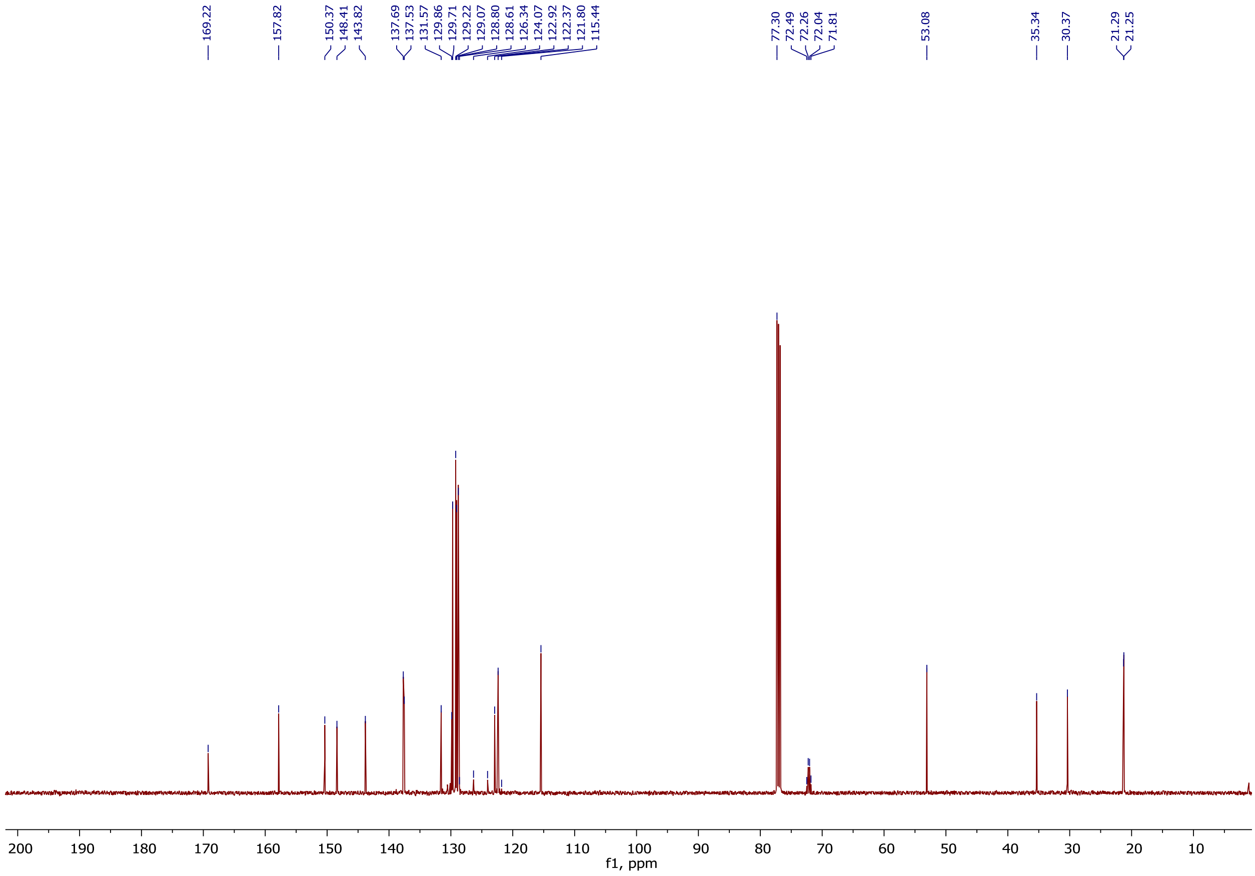

^{13}C spectrum of **4l** in $CDCl_3$

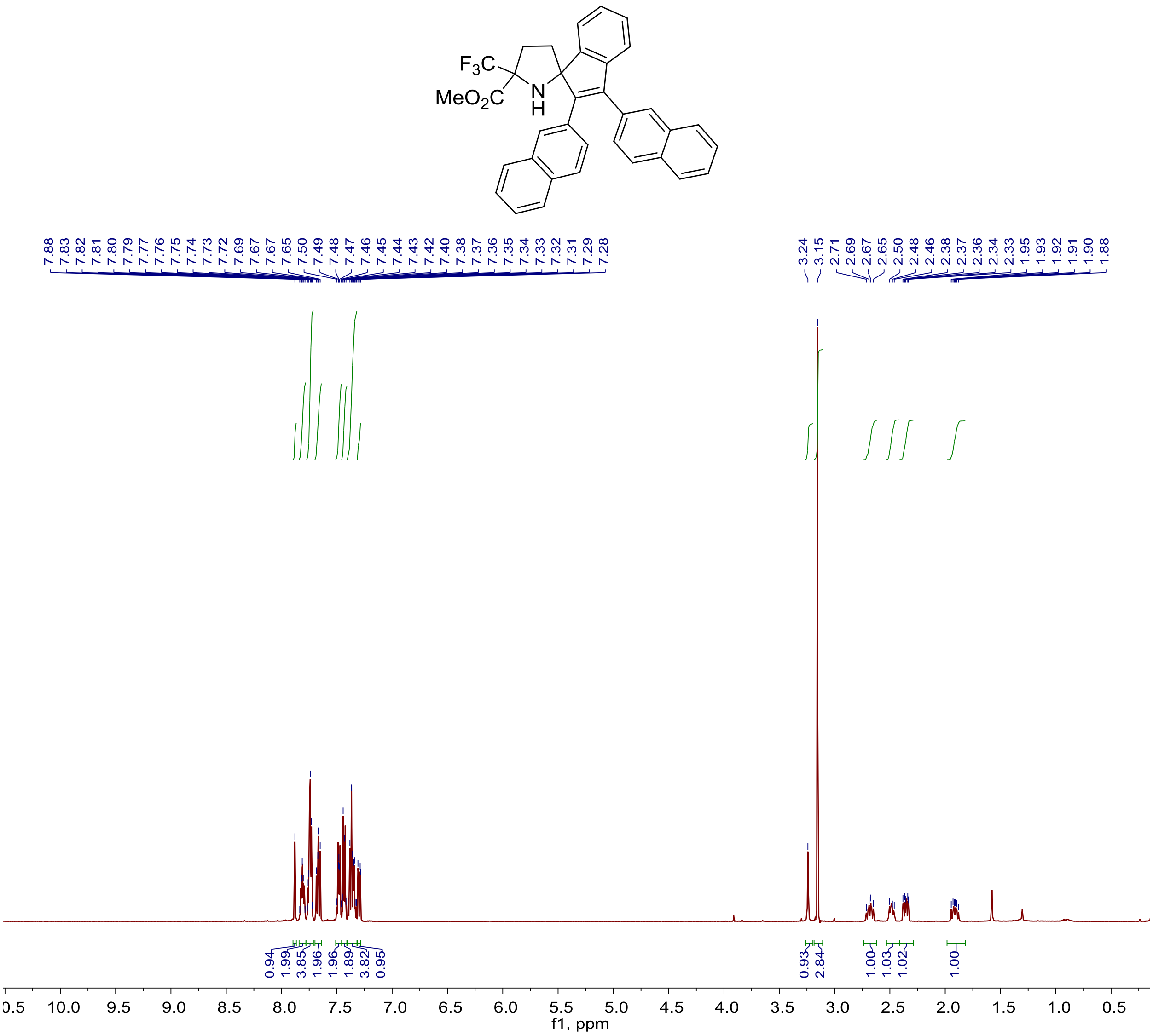

^{1}H spectrum of **4m** in $CDCl_3$

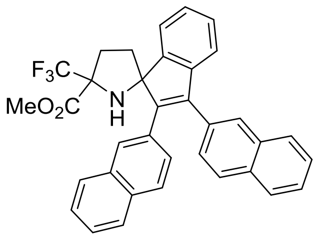

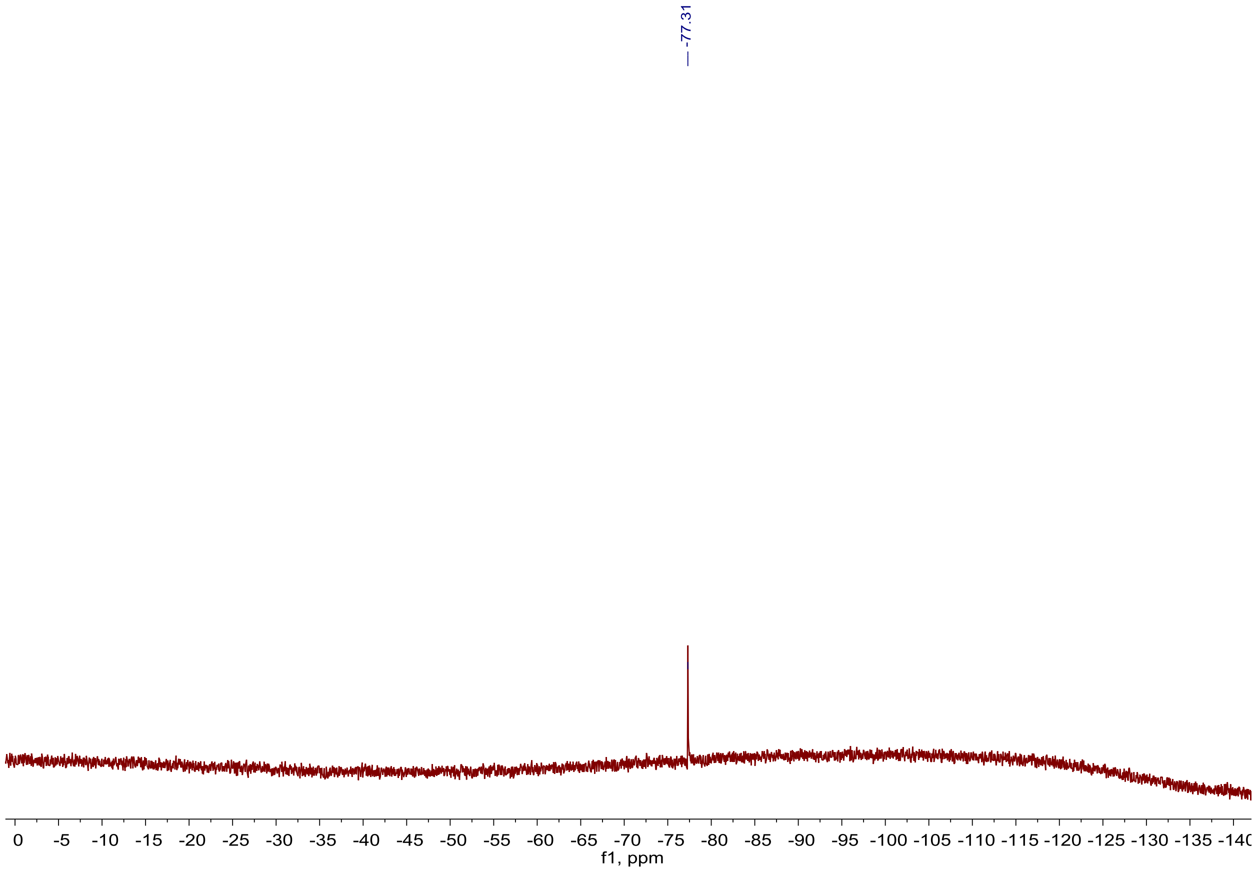

^{19}F spectrum of **4m** in $CDCl_3$

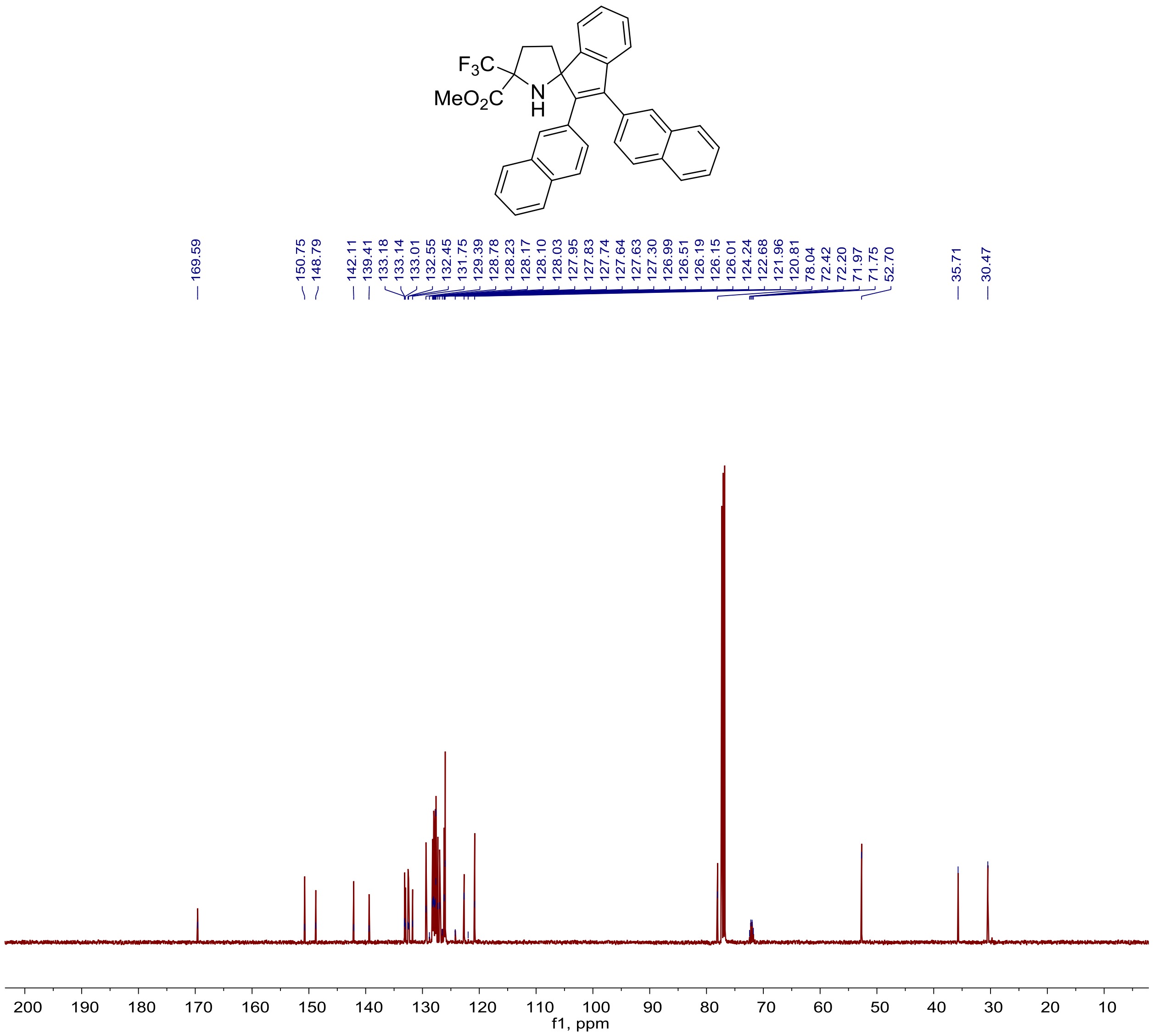

^{13}C spectrum of **4m** in $CDCl_3$

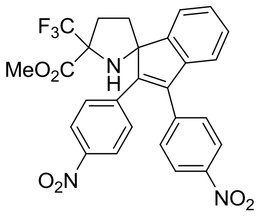

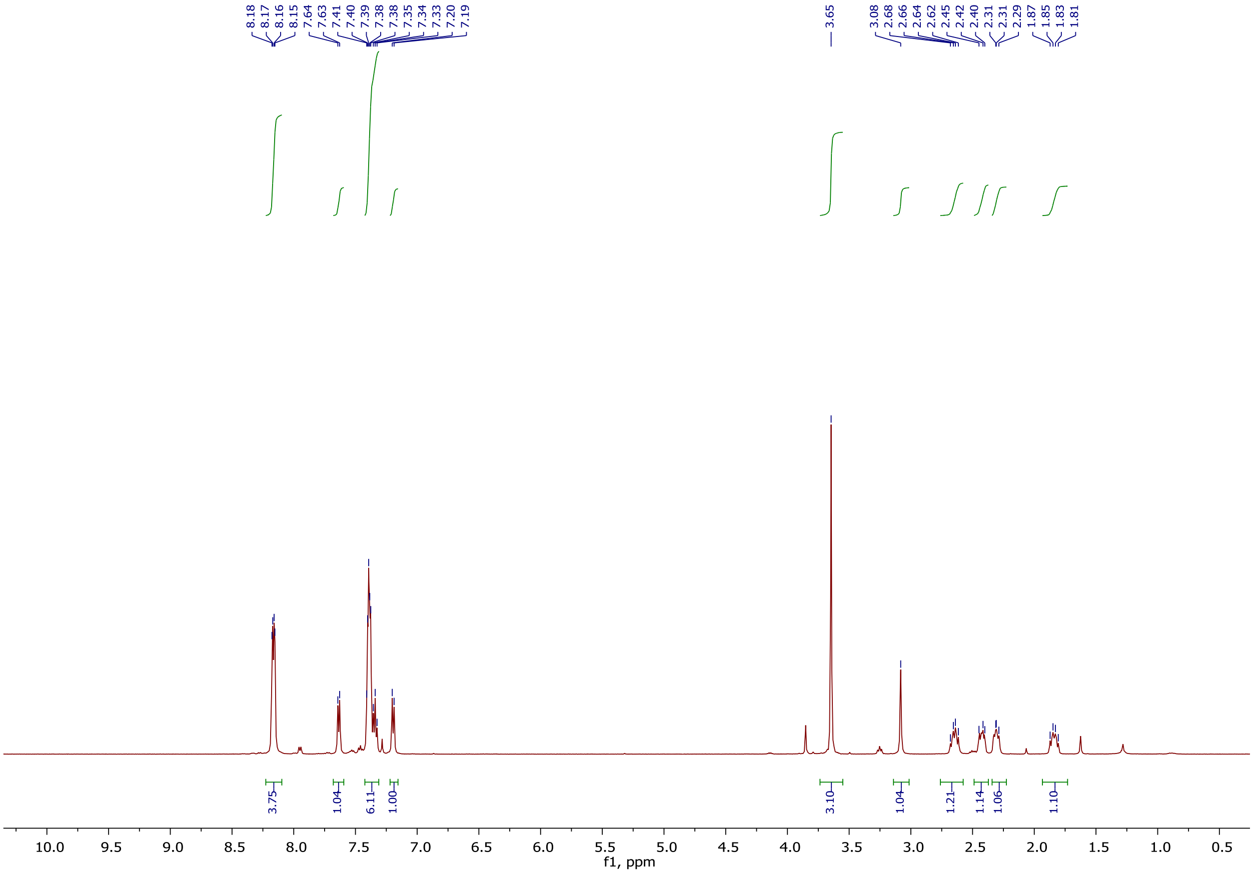

^{1}H spectrum of **4n** in $CDCl_3$

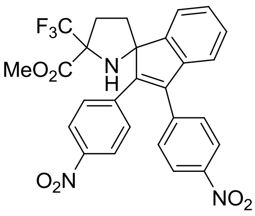

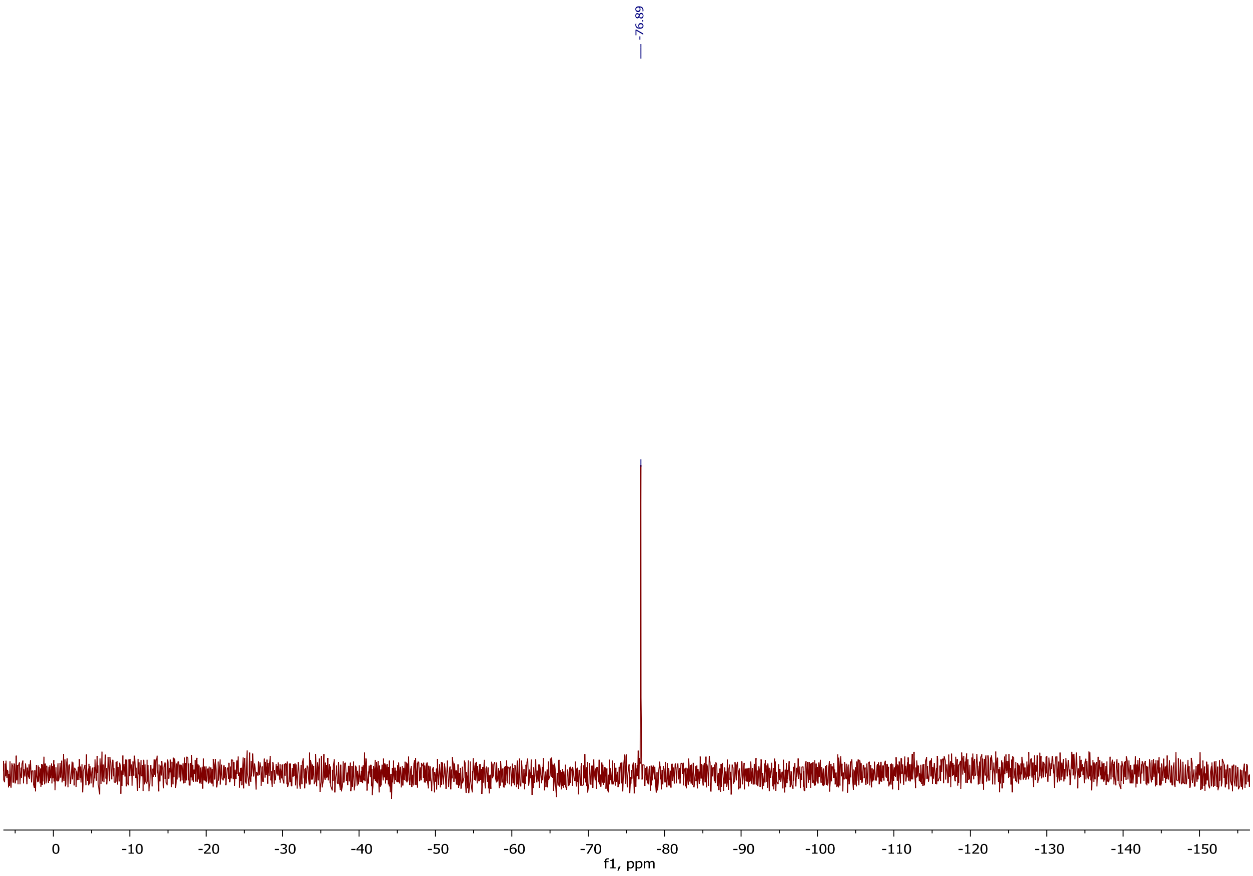

^{19}F spectrum of **4n** in $CDCl_3$

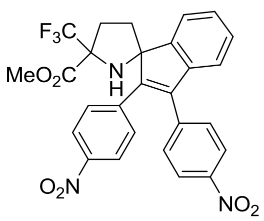

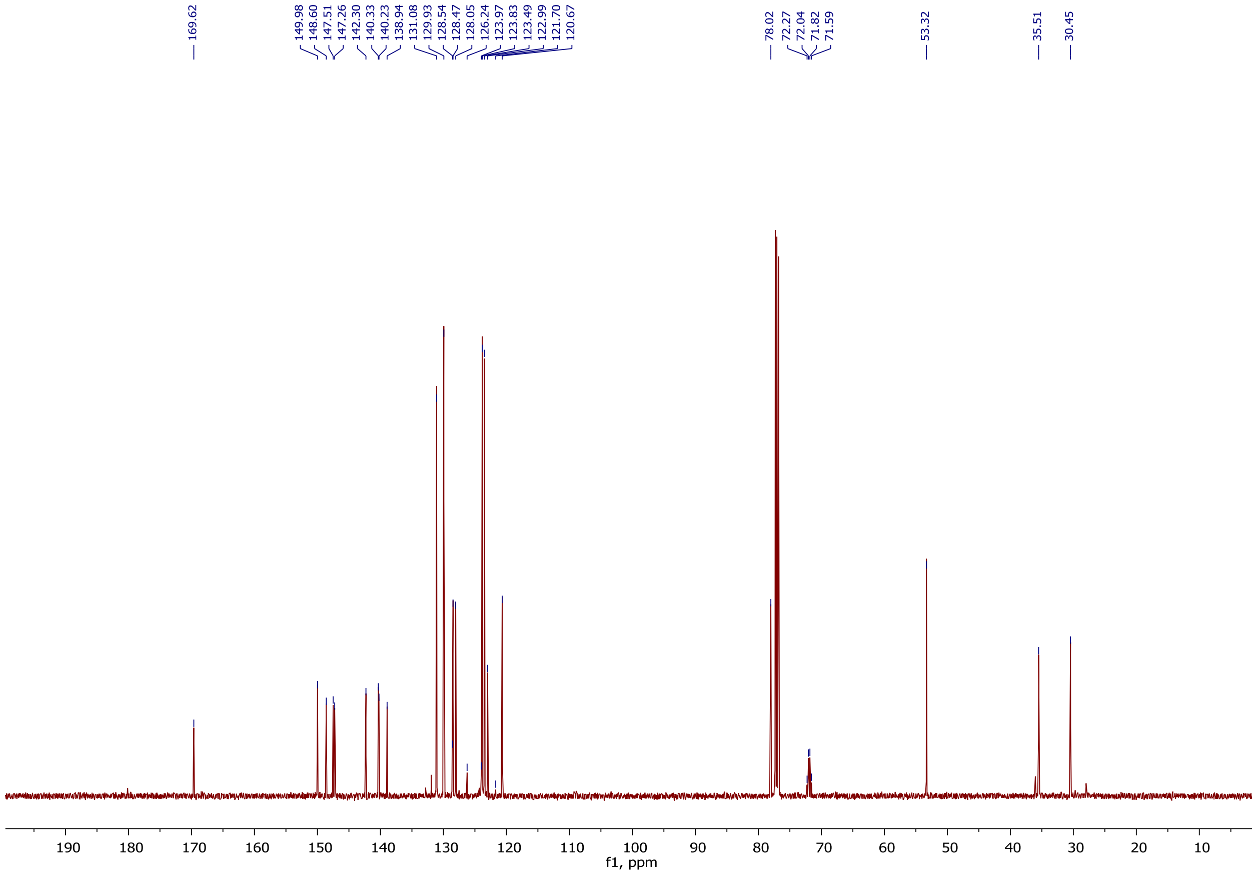

^{13}C spectrum of **4n** in $CDCl_3$

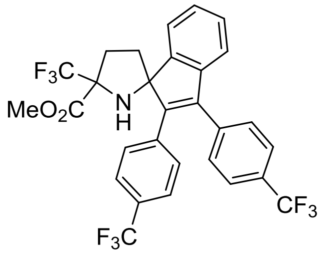

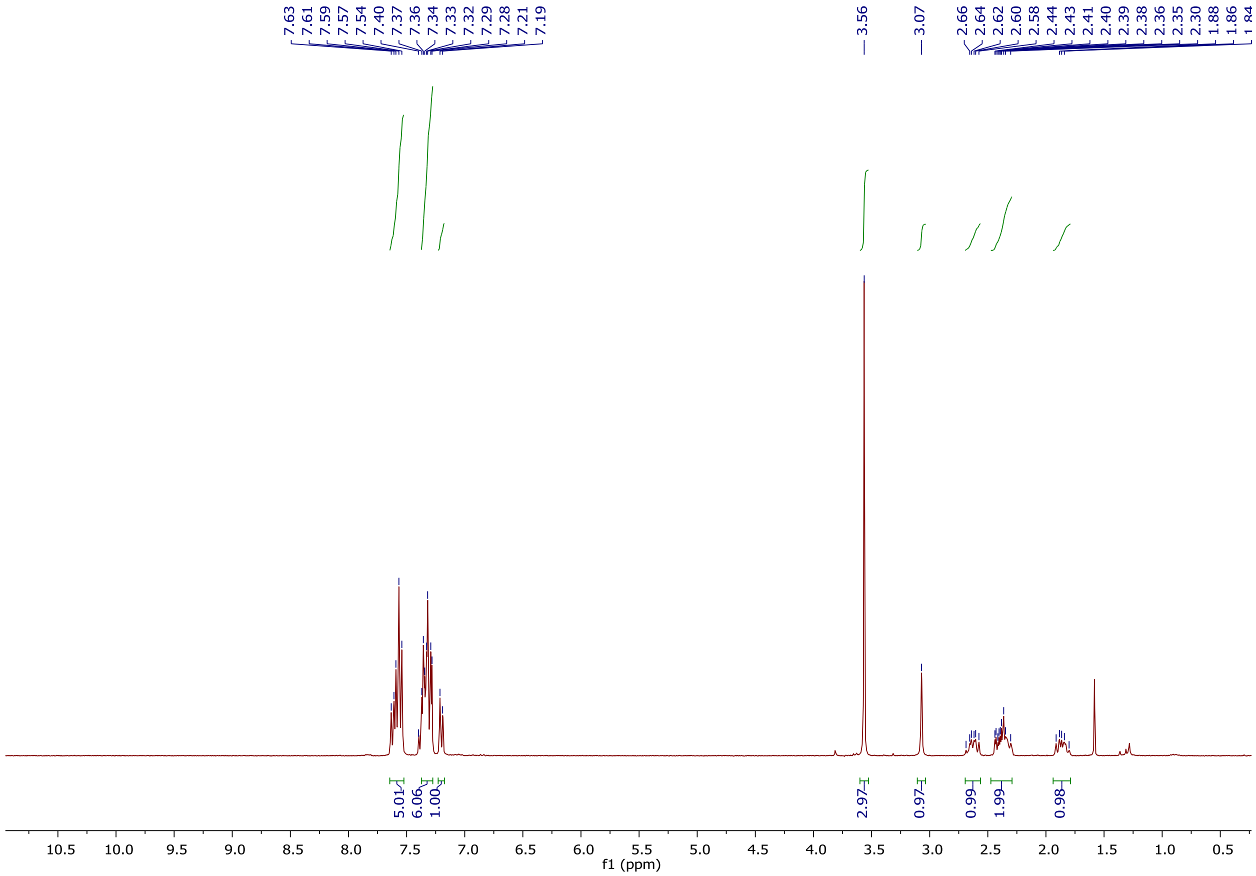

^{1}H spectrum of **4o** in $CDCl_3$

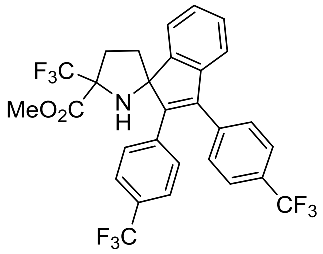

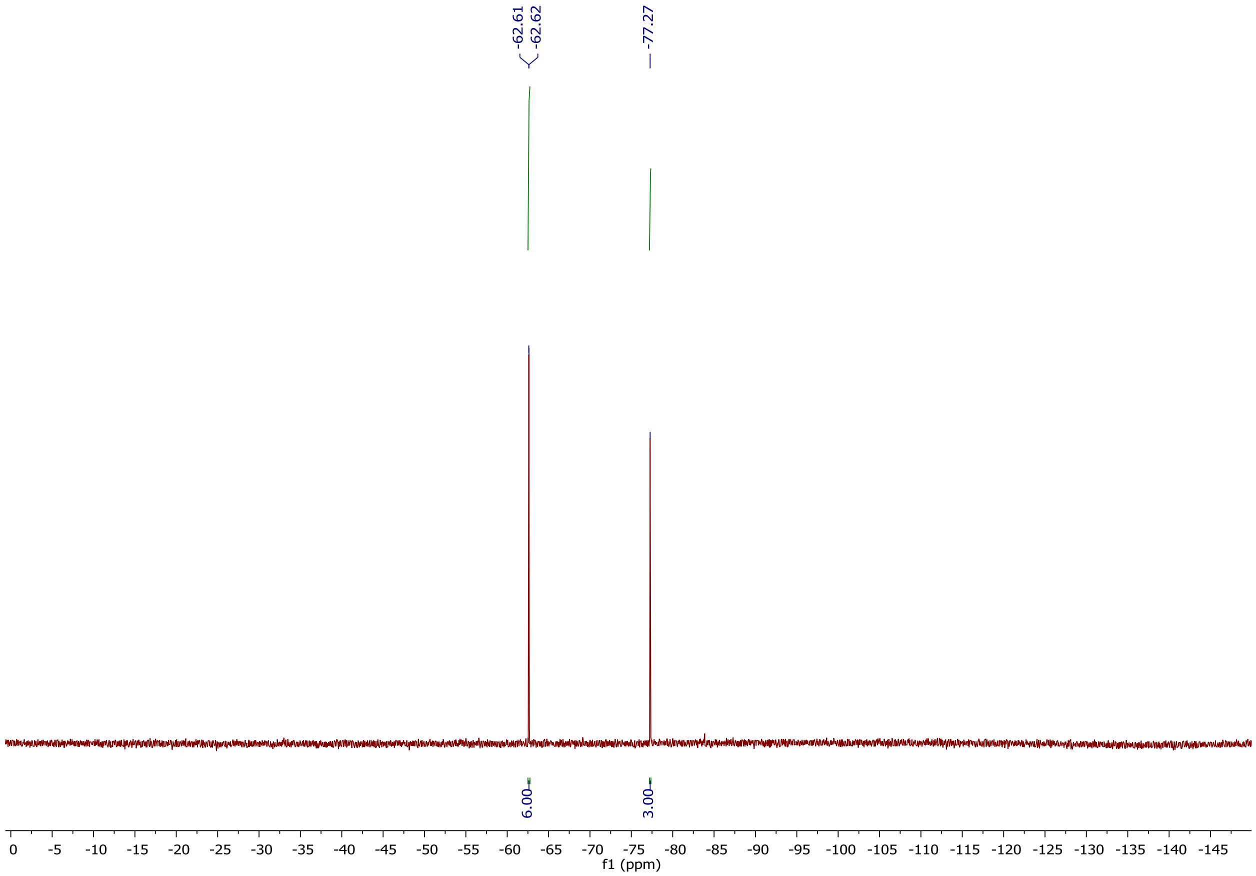

^{19}F spectrum of **4o** in $CDCl_3$

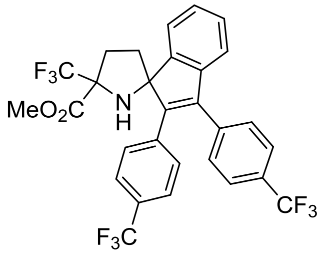

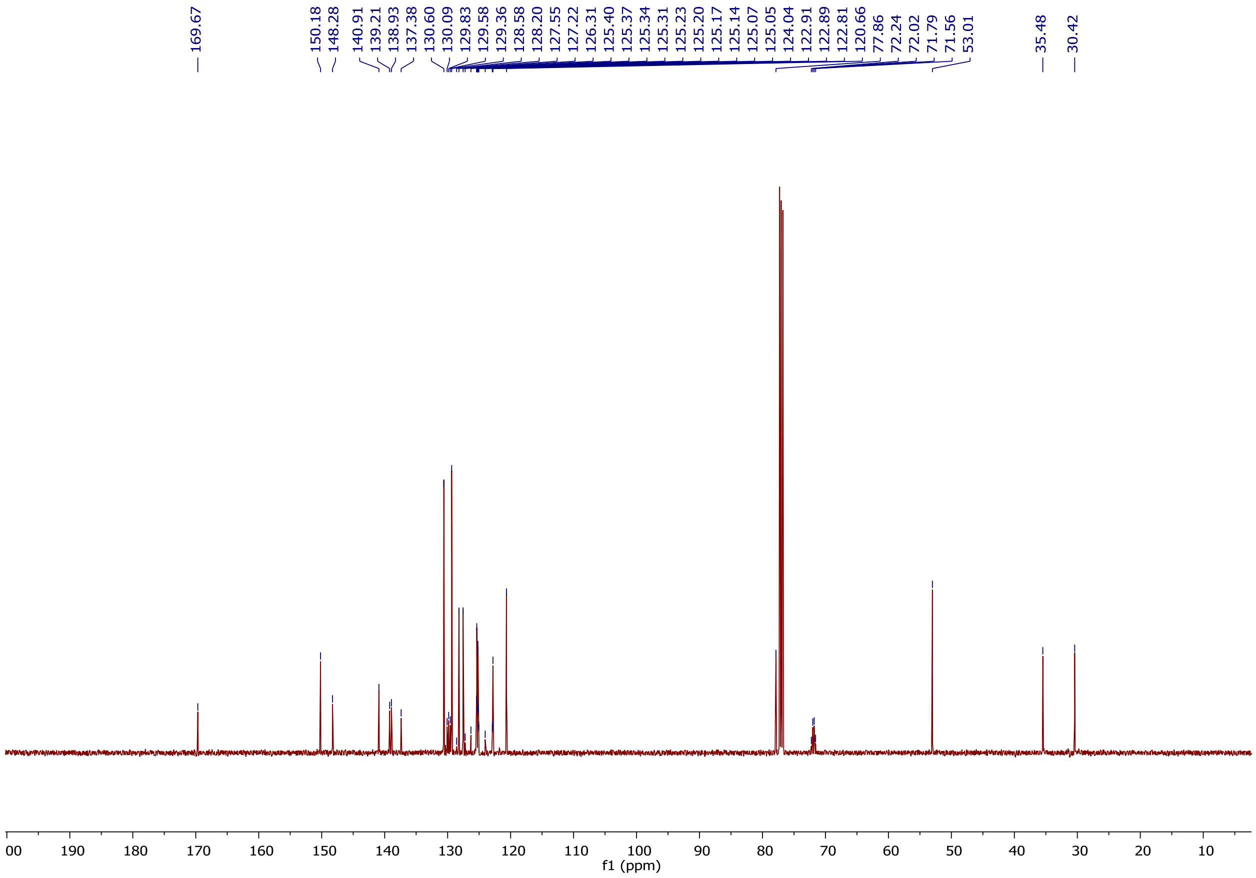

^{13}C spectrum of **4o** in $CDCl_3$

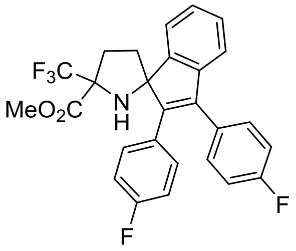

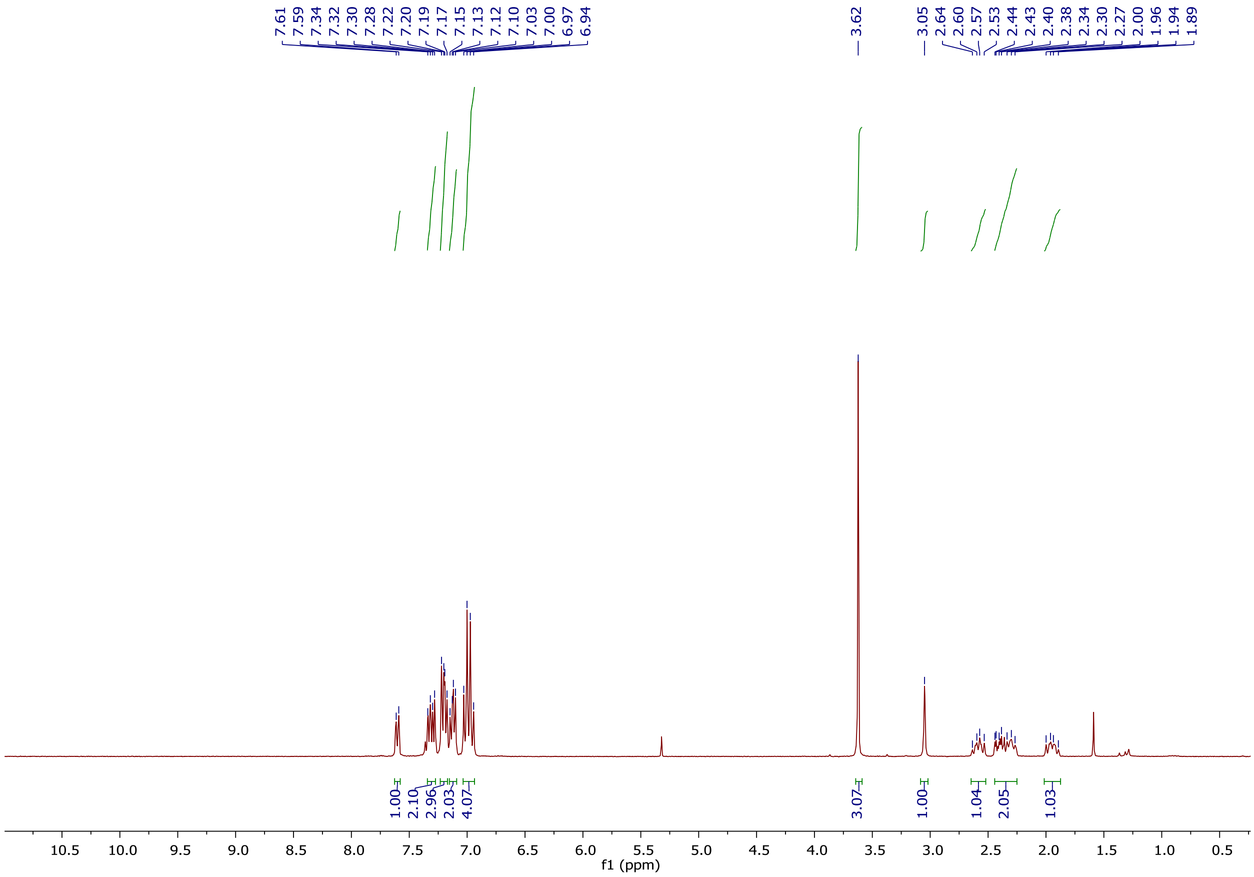

^{1}H spectrum of **4p** in $CDCl_3$

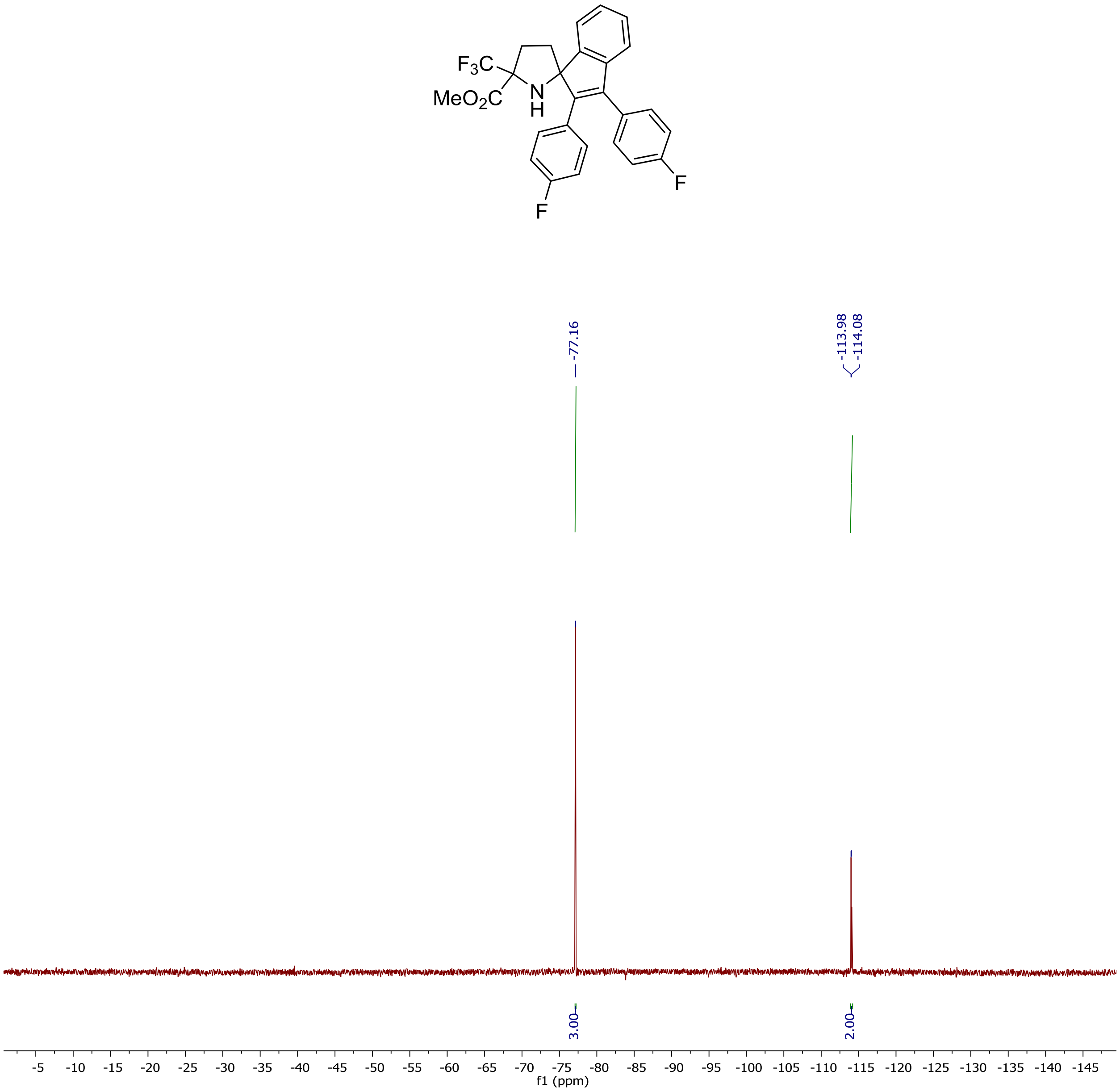

^{19}F spectrum of **4p** in $CDCl_3$

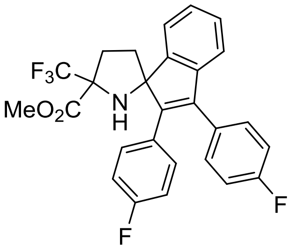

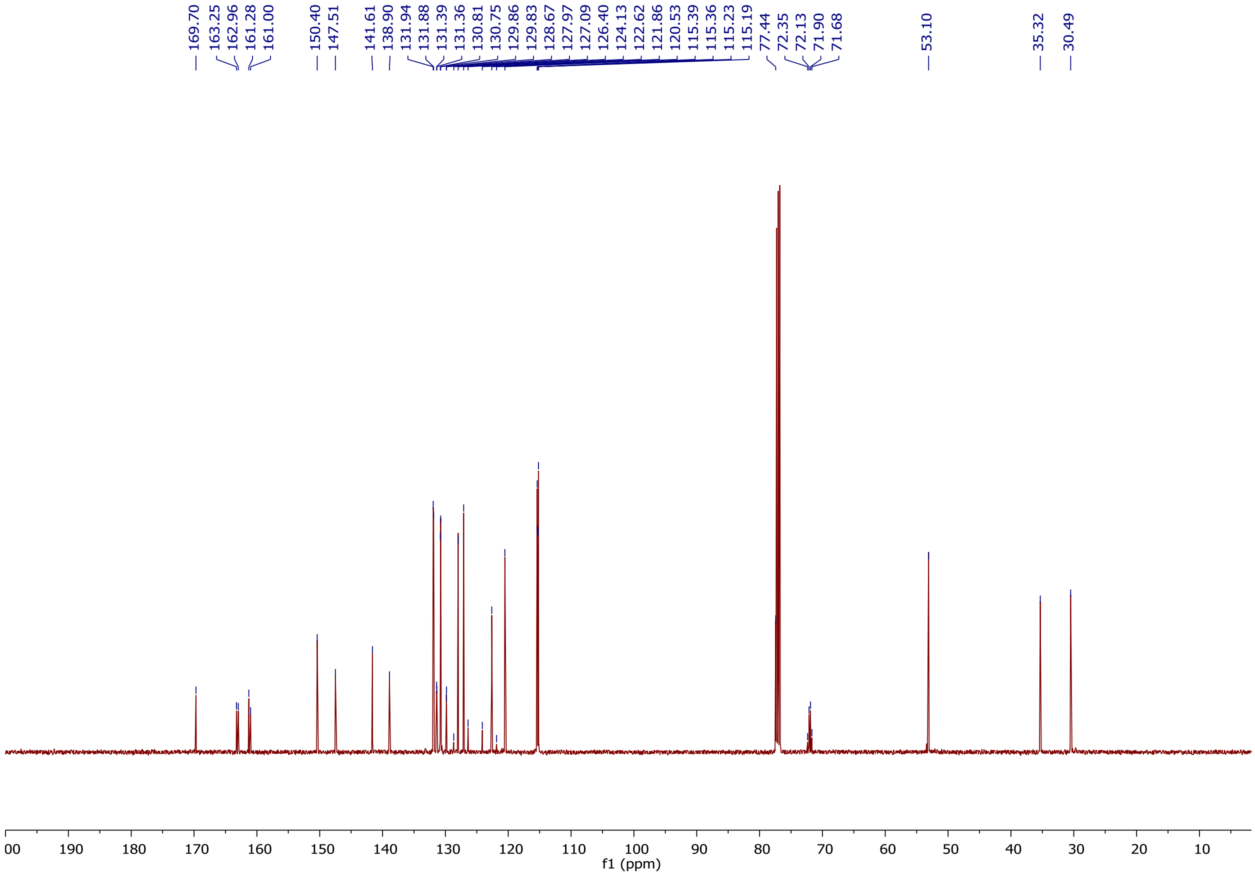

^{13}C spectrum of **4p** in $CDCl_3$

3. HPLC-HRMS data for 4a

High-resolution mass spectra were recorded on a Bruker Daltonics micrOTOF-Q II device (electrospray ionization). Measurements were carried out in positive ion mode under the following conditions: spray capillary voltage 4500 V; the mass scanning range m/ z 50–3000 Da; external calibration (Electrospray Calibrant Solution, Fluka); nebulizer pressure 04 bar; flow rate 3 μl·min−1 ; nebulizer gas—nitrogen (6.0 l·min−1); and interface temperature 180 °C. Samples were injected into the spray chamber of the mass spectrometer from an Agilent 1260 liquid chromatograph equipped with an Agilent Poroshell 120 EC-C18 column (3.0 × 50 mm; 2.7 μm); the flow rate was 0.2 ml min−1 ; the samples of compounds were loaded to an high-performance liquid chromatography (HPLC) chromatograph from acetonitrile solution and eluted in the following gradient of acetonitrile (A) in water: 0–6 min – 40%-85% A, 6–7.5 min – 85% A, 7.5–8 min – 85%-40% A, 8–10 min – 40% A.

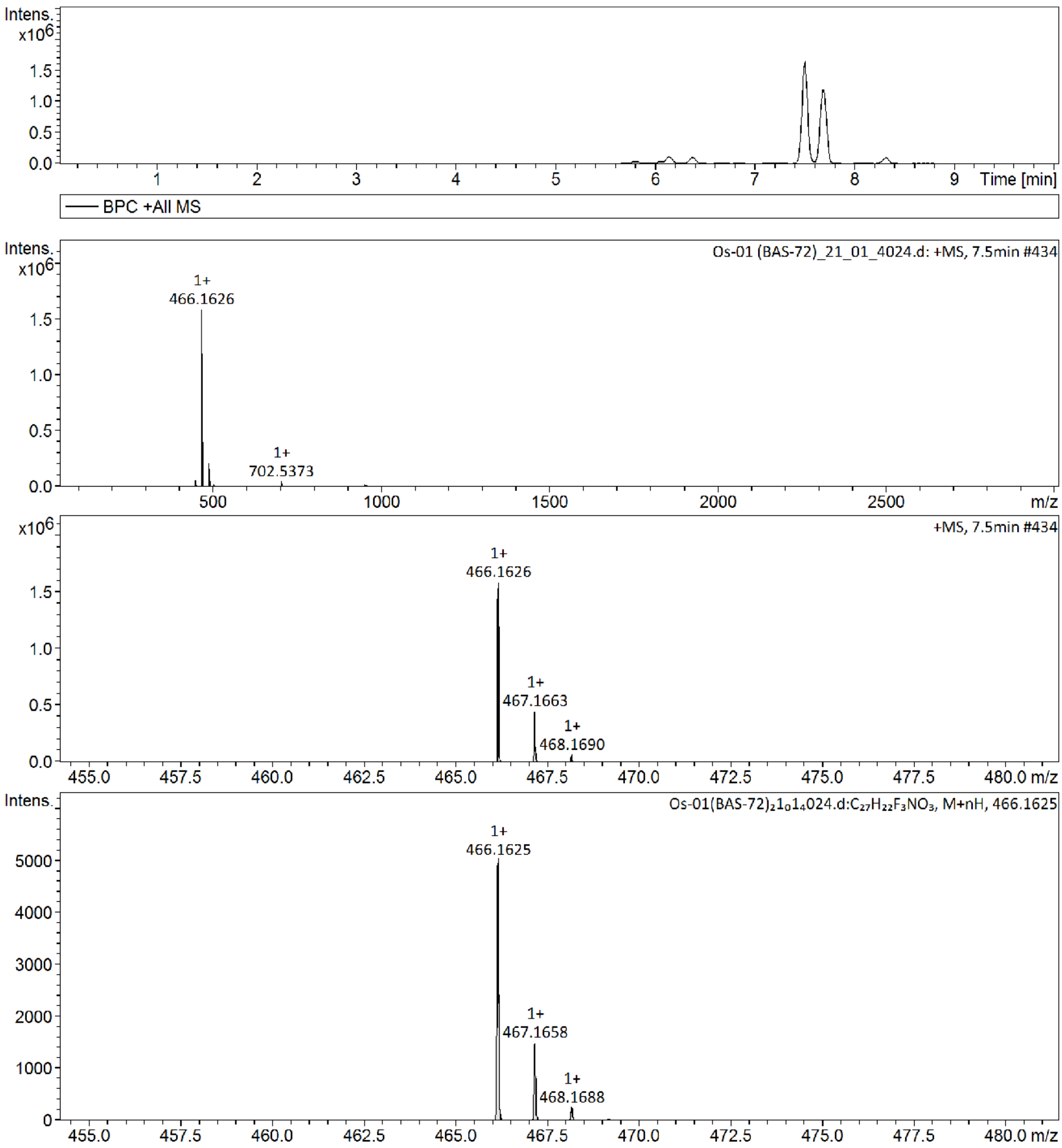
BPC +All MS
Os-01 (BAS-72)_21_01_4024.d: +MS, 7.5min #434
1+
466.1626
1+
702.5373
+MS, 7.5min #434
1+
466.1626
1+
467.1663
1+
468.1690
Os-01(BAS-72)21014024.d:C27H22F3NO3, M+nH, 466.1625
1+
466.1625
1+
467.1658
1+
468.1688
Intens.
x10^6
Time [min]
m/z
